# Antioxidant and Antibacterial Properties of Carbosilane Dendrimers Functionalized with Polyphenolic Moieties

**DOI:** 10.3390/pharmaceutics12080698

**Published:** 2020-07-24

**Authors:** Natalia Sanz del Olmo, Cornelia E. Peña González, Jose Daniel Rojas, Rafael Gómez, Paula Ortega, Alberto Escarpa, Francisco Javier de la Mata

**Affiliations:** 1 Department of Organic and Inorganic Chemistry, and Research Institute in Chemistry “Andrés M. del Río” (IQAR), 28871 Alcalá de Henares, Spain; n.sanzdelolmo@gmail.com (N.S.d.O.); cornelia.pena@uah.es (C.E.P.G.); rafael.gomez@uah.es (R.G.); 2 Networking Research Center on Bioengineering, Biomaterials and Nanomedicine (CIBER-BBN), Instituto Ramon y Cajal de Investigacion Sanitaria, IRYCIS, Colmenar Viejo Road, Km 9, 100, 28034 Madrid, Spain; 3 Department of Analytical Chemistry, Physical Chemistry and Chemical Engineering, University of Alcalá, Institute in Chemistry “Andrés M. del Río” (IQAR), 28871 Alcalá de Henares, Spain; j.daniel.rojas.t@gmail.com (J.D.R.); alberto.escarpa@uah.es (A.E.); 4 Faculty of Bioscience and Technology for Food, Agriculture and Environment University of Teramo, 64023 Teramo, Italy

**Keywords:** antioxidants, polyphenols, carbosilane dendrimers, antibacterial

## Abstract

A new family of polyphenolic carbosilane dendrimers functionalized with ferulic, caffeic, and gallic acids has been obtained through a straightforward amidation reaction. Their antioxidant activity has been studied by different techniques such as DPPH (2,2′-diphenyl-1-picrylhydrazyl) radical scavenging assay, FRAP assay (ferric reducing antioxidant power), and cyclic voltammetry. The antioxidant analysis showed that polyphenolic dendrimers exhibited higher activities than free polyphenols in all cases. The first-generation dendrimer decorated with gallic acid stood out as the best antioxidant compound, displaying a correlation between the number of hydroxyl groups in the polyphenol structure and the antioxidant activity of the compounds. Moreover, the antibacterial capacity of these new systems has been screened against Gram-positive (+) and Gram-negative (−) bacteria, and we observed that polyphenolic dendrimers functionalized with caffeic and gallic acids were capable of decreasing bacterial growth. In contrast, ferulic carbosilane dendrimers and free polyphenols showed no effect, establishing a correlation between antioxidant activity and antibacterial capacity. Finally, a viability assay in human skin fibroblasts cells (HFF-1) allowed for corroborating the nontoxicity of the polyphenolic dendrimers at their active antibacterial concentration.

## 1. Introduction

The accumulation of free radicals caused by oxidative stress has been associated with the aging process as well as several age-related conditions (i.e., cardiovascular diseases, cancer, and degenerative diseases) [1]. Free radicals produce mutations in biological macromolecules (mainly DNA, lipids, and proteins) that cause structural cell damage [2]. The harmful effects of these species can be controlled by several antioxidant protection systems, which include antioxidant agents that can be produced by the organism (endogenous) or obtained from food (exogenous). Within the essential antioxidant agents are the polyphenolic compounds that can be found in plants [3]. Polyphenols could modulate the activity of different enzymes, and consequently, interfere with signaling mechanisms and different cellular processes. This behavior can be attributed to their physicochemical characteristics, which allow them to participate in different redox cellular metabolic reactions. However, the concept of bioavailability is significant, since they are generally poorly absorbed in the gut, highly metabolized, or rapidly excreted [4]. To overcome this drawback, nanotechnology has become a promising alternative.

The development of different nanomaterials, such as nanoparticles [5,6], silica matrices [7], or dendrimers [8,9,10], as antioxidant delivery agents has received particular interest in recent years. Within these systems, dendrimers exhibit interesting characteristics that could allow them to be used as platforms to be functionalized with polyphenols. The presence of multiple branches in its structure allows for the introduction of a large number of polyphenolic groups in a single molecule, either by electrostatic interactions or covalent bonds, which could improve the antioxidant activity; moreover, their monodispersity make them ideal for biomedical uses. Nevertheless, only a few examples have been reported so far in the literature [8,9,10,11,12].

In 2016, our research group described a new family of polyphenolic carbosilane dendrimers functionalized with vanillin that showed promising antioxidant activity, as well as high potential as anticancer agents against an advanced prostate cancer cell line (PC-3) [9]. The high stability and very low hydrophilicity of the carbosilane skeleton might improve their interaction with membranes [13,14,15]. As a new goal, our research group focused its attention on three polyphenolic acids, ferulic (3-(4-hydroxy-3-methoxyphenyl)-2-propenoic acid), caffeic (3-3,4-dihydroxyphenyl)-2-propenoic acid), and gallic (3,4,5-trihydroxybenzoic acid), due to their antioxidant activity and potential for application in the biomedical field. Ferulic acid acts not only as a radical scavenger but also as an inhibitor of enzymes that catalyzes free radical production [16]; in addition, this compound has shown interesting anti-inflammatory effects [17]. In addition, caffeic acid has been seen to rise as a defense against oxidative stress in different diseases like renal dysfunction [18]. Finally, gallic acid has been reported as a promising anticancer and anti-inflammatory agent [19,20].

The main objective of this work is the functionalization of carbosilane dendrimers’ periphery with ferulic, caffeic, and gallic acids, and an evaluation of them as antioxidant and antibacterial agents. Different spectrophotometric assays such as DPPH (2,2′-diphenyl-1-picrylhydrazyl) and ferric reducing antioxidant power (FRAP) assay, as well as an electrochemical evaluation of their charge-transfer properties, allows us to determine its antioxidant activity and evaluate whether the number of polyphenols on the dendritic skeleton affects the antioxidant behavior. Furthermore, the antibacterial properties of the new polyphenolic carbosilane dendrimers are determined against *Staphylococcus aureus* (Gram-positive (+)) and *Escherichia coli* (Gram-negative (−)) and compared with free polyphenols. Furthermore, the cell viability of human skin fibroblasts after treatment with polyphenolic dendrimers is established.

## 2. Materials and Methods

### 2.1. Synthesis and Characterization of Polyphenolic Dendrimers (1–6)

All reactions have been carried out under an inert atmosphere and solvents of reactions have been bought in dry conditions. NMR experiments have been carried out on a Varian 500 Hz instrument. Chemical shifts (δ) are given in ppm. As a reference, deuterated methanol solvent (CD_3_OD) has been used. Assignment of resonances was done from HSQC, HMBC, and COSY NMR experiments. Elemental analyses were performed on a LECO CHNS-932 instrument. Mass spectra were obtained using the ESI-TOF technique from an Agilent 6210 TOF LC/MS instrument in MeOH, and ACN/H_2_O 0.1% formic acid as the mobile phase.

Spectra obtained through the different techniques for the new compounds described in this work are collected in the Supplementary Materials (Figures S1–S41).

#### 2.1.1. Synthesis of G_1_-[Si(CH_2_)_3_NH(CO)CH=CHCH_2_Ph(OH)(OCH_3_))]_4_ (**1**)

To achieve the synthesis of compound **1**, the activation of ferulic acid (179.0 mg, 0.922 mmol) with EDCI·HCl (176.7 mg, 0.922 mmol) and HOBt (124.6 mg, 0.922 mmol), using dry DMF as solvent for the reaction, was carried out in the first place. The mixture was stirred at room temperature for one hour. Afterwards, a DMF solution mixture of dendrimer functionalized with amine groups G_1_-[Si(CH_2_)_3_NH_2_]_4_ (127.0 mg, 0.192 mmol) and NEt_3_ (0.768 mmol) was added dropwise under stirring and at 0 °C. After five minutes in these conditions, the mixture was kept at 60 °C, overnight. The compound was purified by size exclusion chromatography in DMF, obtaining compound **1** as a brown solid (178.4 mg, 68%). ^1^H-NMR (CD_3_OD): δ (ppm) 0.00 (s, 24H, NHCH_2_CH_2_CH_2_Si(CH_3_)_2_), 0.51–0.67 (m, overlapping of signals, 24H, SiCH_2_CH_2_CH_2_Si and NHCH_2_CH_2_CH_2_Si(CH_3_)_2_), 1.39 (m, 8H, SiCH_2_CH_2_CH_2_Si ), 1.57 (m, 8H, NHCH_2_CH_2_CH_2_Si(CH_3_)_2_), 3.29 (m, 8H, NHCH_2_CH_2_CH_2_Si(CH_3_)_2_), 3.88 (s, 12H, OCH_3_), 6.48 (d, 4H, ^3^J_(H-H)_ = 15.7 Hz, PhCH=CH(CO)NH), 6.81 (dd, 4H, ^3^J_(H-H)_ = 8.2 Hz, ^5^J_(H-H)_ = 1.8 Hz, 1H_Ar_, *ortho*-OH), 7.04 (d, 4H, ^3^J_(H-H)_ = 8.2 Hz, 1H_Ar_, *para*-OCH_3_), 7.12 (d, 4H, ^5^J_(H-H)_ = 1.8 Hz, 1H_Ar_, *ortho*-OCH_3_), 7.47 (d, 4H, ^3^J_(H-H)_ = 15.7 Hz, PhCH=CH(CO)NH). ^13^C-NMR (CD_3_OD): δ (ppm) −3.1 ((CH_3_)_2_SiCH_2_CH_2_CH_2_NH), 13.6 ((CH_3_)_2_SiCH_2_CH_2_CH_2_NH), 18.6, 19.9, 21.1 (SiCH_2_CH_2_CH_2_Si), 25.2 ((CH_3_)_2_SiCH_2_CH_2_CH_2_NH), 43.9 ((CH_3_)_2_SiCH_2_CH_2_CH_2_NH), 56.4 (OCH_3_), 111.6 (CAr, *ortho*-OCH_3_), 116.5 (CAr, *ortho*-OH), 118.9 (CAr, PhCH=CH(CO)NH), 123.2, (CAr, *para*-OCH_3_), 128.3 (Cipso, *para*-OH), 142.0 (PhCH=CH(CO)NH), 149.3 (Cipso), 149.8 (Cipso), 169.1 (NH*C*=O). {^1^H-^15^N}-HMBC-NMR (CD_3_OD): δ (ppm) −259.4 (NHC=O). MS: [M+H]^+^ = 1366.7266 u (Calc. 1366.7264 u), [M+Na]^+^ = 1388.7061 u (Calc. 1388.7063 u). Elemental Analysis (%): Calc for C_72_H_112_N_4_O_12_Si_5_ (1366.13 g/mol). C, 63.30; H, 8.26; N, 4.10. Exp.: C, 62.9; H, 8.26; N, 5.05.

#### 2.1.2. Synthesis of G_1_-[Si(CH_2_)_3_NH(CO)CH=CHCH_2_Ph(OH)_2_)]_4_ (**2**)

Dendrimer **2** has been prepared through the same method as described for **1** by using the following reagents: caffeic acid (255.1 mg, 1.416 mmol), EDCI·HCl (271.4 mg, 1.416 mmol), HOBt (191.3 mg, 1.416 mmol), G_1_-[Si(CH_2_)_3_NH_2_]_2_ (195.3 mg, 0.295 mmol) and NEt_3_ (1.180 mmol). Compound **2** was obtained as a brown solid (231.9 mg, 61%). ^1^H-NMR (CD_3_OD): δ (ppm) −0.04 (s, 24H, NHCH_2_CH_2_CH_2_Si(CH_3_)_2_), 0.48–0.67 (m, overlapping of signals, 24H, SiCH_2_CH_2_CH_2_Si and NHCH_2_CH_2_CH_2_Si(CH_3_)_2_), 1.36 (m, 8H, SiCH_2_CH_2_CH_2_Si), 1.53 (m, 8H, NHCH_2_CH_2_CH_2_Si(CH_3_), 3.24 (m, 8H, NHCH_2_CH_2_CH_2_Si(CH_3_)_2_), 6.37 (d, 4H, ^3^J_(H-H)_ = 15.7 Hz, PhCH=CH(CO)NH), 6.75 (d, 4H, ^3^J_(H-H)_ = 8.2 Hz, 1H_Ar_, *meta*-CH=CH), 6.89 (dd, 4H, ^3^J_(H-H)_ = 8.2 Hz, ^5^J_(H-H)_ = 2.0 Hz, 1H_Ar_, *ortho*-CH=CH), 7.00 (d, 4H, ^5^J_(H-H)_ = 2.0 Hz, 1H_Ar_, *ortho*-CH=CH, *ortho*-OH), 7.38 (d, 4H, ^3^J_(H-H)_ = 15.7 Hz, PhCH=CH(CO)NH). ^13^C-NMR (CD_3_OD): δ (ppm) −3.1 ((CH_3_)_2_SiCH_2_CH_2_CH_2_NH), 13.6 ((CH_3_)_2_SiCH_2_CH_2_CH_2_NH), 18.5, 19.9, 21.0 (SiCH_2_CH_2_CH_2_Si), 25.2 ((CH_3_)_2_SiCH_2_CH_2_CH_2_NH), 43.9 ((CH_3_)_2_SiCH_2_CH_2_CH_2_NH), 115.1 (CAr, *ortho*-OH, *ortho*-CH=CH), 116.5 (CAr, *meta*-CH=CH), 118.5 (PhCH=CH(CO)NH), 122.1, (CAr, *ortho-CH=CH*), 128.3 (Cipso, *meta*-OH, *para*-OH), 142.1 (PhCH=CH(CO)NH), 146.7 (Cipso), 148.7 (Cipso), 169.2 (NH*C*=O). {^1^H-^15^N}-HMBC-NMR (CD_3_OD): δ (ppm) −259.5 (*N*HC=O). MS: [M+H]^+^ = 1310.6618 u (Calc. 1310.6670 u), [M+Na]^+^ = 1332.6431 u (Calc. 1332.6433 u). Elemental Analysis (%): Calc for C_68_H_104_N_4_O_12_Si_5_ (1310.02 g/mol). C, 62.35; H, 8.00; N, 4.28. Exp.: C, 60.23; H, 7.52; N, 4.50.

#### 2.1.3. Synthesis of G_1_-[Si(CH_2_)_3_NH(CO)Ph(OH)_3_]_4_ (**3**)

Dendrimer **3** has been prepared through the same method as described for **1** by using the following reagents: gallic acid (252.9 mg, 1.344 mmol), EDCI·HCl (257.6 mg, 1.344 mmol), HOBt (181.6 mg, 1.344 mmol), G_1_-[Si(CH_2_)_3_NH_2_]_4_ (185.0 mg, 0.280 mmol) and NEt_3_ (1.120 mmol). Compound **3** was obtained as a brown solid (248.9 mg, 70%). ^1^H-NMR (CD_3_OD): δ (ppm) 0.00 (s, 24H, NHCH_2_CH_2_CH_2_Si(CH_3_)_2_), 0.46–0.71 (m, overlapping of signals, 24H, SiCH_2_CH_2_CH_2_Si and NHCH_2_CH_2_CH_2_Si(CH_3_)_2_), 1.40 (m, 8H, SiCH_2_CH_2_CH_2_Si), 1.59 (m, 8H, NHCH_2_CH_2_CH_2_Si(CH_3_), 3.31 (m, 8H, NHCH_2_CH_2_CH_2_Si(CH_3_)_2_), 6.88 (s, 8H, H_Ar_), 7.07–7.32 (m, overlapping of signals, 16H, NH and Ph(OH)). ^13^C-NMR (CD_3_OD): δ (ppm) −3.1 ((CH_3_)_2_SiCH_2_CH_2_CH_2_NH), 13.6 ((CH_3_)_2_SiCH_2_CH_2_CH_2_NH), 18.6, 19.9, 21.1 (SiCH_2_CH_2_CH_2_Si), 25.2 ((CH_3_)_2_SiCH_2_CH_2_CH_2_NH), 44.3 ((CH_3_)_2_SiCH_2_CH_2_CH_2_NH), 107.8 (CAr, *ortho*-OH), 126.4 (Cipso, *para*-OH), 146.6 (Cipso), 170.5 (NHC=O). {^1^H-^15^N}-HMBC-NMR (CD_3_OD): δ (ppm) −267.0 (NHC=O). MS: [M+H]^+^ = 1269.5762 u (Calc. 1269.5740 u). Elemental Analysis (%): Calc for C_60_H_96_N_4_O_16_Si_5_ (1269.87 g/mol). C, 56.75; H, 7.62; N, 4.41. Exp.: C, 56.60; H, 7.38; N, 4.95.

#### 2.1.4. Synthesis of G_2_-[Si(CH_2_)_3_NH(CO)CH=CHCH_2_Ph(OH)(OCH_3_))]_8_ (**4**)

Dendrimer **4** has been prepared through the same method as described for **1** by using the following reagents: ferulic acid (259.5 mg, 1.336 mmol), EDCI·HCl (255.6 mg, 1.336 mmol), HOBt (180.6 mg, 1.366 mmol), G_2_-[Si(CH_2_)_3_NH_2_]_8_ (166.0 mg, 0.139 mmol) and NEt_3_ (1.112 mmol). Compound **4** was obtained as a brown solid (296.3 mg, 70%).^1^H-NMR (CD_3_OD): δ (ppm) −0.03 (s, 12H, CH_3_(CH_2_CH_2_CH_2_Si)_2_), 0.00 (s, 48H, -(CH_3_)_2_SiCH_2_CH_2_CH_2_NH), 0.48–0.70 (m, overlapping of signals, 64H, -SiCH_2_CH_2_CH_2_Si, CH_3_Si(CH_2_CH_2_CH_2_Si)_2_ and (CH_3_)_2_SiCH_2_CH_2_CH_2_NH), 1.30–1.47, (m, 24H, overlapping of signals, SiCH_2_CH_2_CH_2_Si and CH_3_Si(CH_2_CH_2_CH_2_Si)_2_), 1.52–1.62 (m, 16H, (CH_3_)_2_SiCH_2_CH_2_CH_2_NH), 3.29 (m, 16H, (CH_3_)_2_SiCH_2_CH_2_CH_2_NH), 3.86 (s, 24H, OCH_3_), 6.48 (d, 8H, ^3^J_(H-H)_ = 16.2 Hz, PhCH=CH(CO)NH), 6.80 (d, 8H, ^3^J_(H-H)_ = 7.9 Hz, 1H_Ar,_ *ortho*-OH), 7.02 (d, 8H, ^3^J_(H-H)_ = 7.9 Hz, 1H_Ar_, *para*-OCH_3_), 7.10 (s, 8H, 1H_Ar_, *ortho*-OCH_3_), 7.47 (d, 8H, ^3^J_(H-H)_ = 16.2 Hz, PhCH=CH(CO)NH). ^13^C-NMR (CD_3_OD): δ (ppm) −4.1 ((CH_3_)_2_SiCH_2_CH_2_CH_2_NH), −2.8 (CH_3_Si(CH_2_CH_2_CH_2_Si)_2_), 13.7 (-(CH_3_)_2_SiCH_2_CH_2_CH_2_NH), 19.8, 20.0, 20.1, 21.1 (SiCH_2_CH_2_CH_2_Si and -CH_3_Si(CH_2_CH_2_CH_2_Si)_2_), 25.3 (CH_3_)_2_SiCH_2_CH_2_CH_2_NH), 44.0 ((CH_3_)_2_SiCH_2_CH_2_CH_2_NH), 56.4 (-OCH_3_), 111.6 (CAr, *ortho*-OCH_3_), 116.5 (CAr, *ortho*-OH), 119.0 (CAr, PhCH=CH(CO)NH), 123.2, (CAr, *para*-OCH_3_), 128.3 (Cipso, *para*-OH), 142.0 (PhCH=CH(CO)NH), 149.2 (Cipso), 149.8 (Cipso), 169.0 (NHC=O). {^1^H-^15^N}-HMBC-NMR (CD_3_OD): δ (ppm) −259.6 (NHC=O). Elemental Analysis (%): Calc for C_160_H_260_N_8_O_24_Si_13_ (3044.98 g/mol). C, 63.11; H, 8.61; N, 3.68. Exp.: C, 64.56; H, 9.01; N, 3.96.

#### 2.1.5. Synthesis of G_2_-[Si(CH_2_)_3_NH(CO)CH=CHCH_2_Ph(OH)_2_)]_8_ (**5**)

Dendrimer **5** has been prepared through the same method as described for **1** by using the following reagents: caffeic acid (300.1 mg, 1.666 mmol), EDCI·HCl (318.7 mg, 1.666 mmol), HOBt (225.1 mg, 1.666 mmol), G_2_-[Si(CH_2_)_3_NH_2_]_8_ (207.0 mg, 0.174 mmol) and NEt_3_ (1.392 mmol). Compound **5** was obtained as a brown solid (218.6 mg, 65%). ^1^H-NMR (CD_3_OD): δ (ppm) −0.04 (s, 12H, CH_3_(CH_2_CH_2_CH_2_Si)_2_), 0.00 (s, 48H, -(CH_3_)_2_SiCH_2_CH_2_CH_2_NH), 0.47–0.69 (m, overlapping of signals, 64H, -SiCH_2_CH_2_CH_2_Si, CH_3_Si(CH_2_CH_2_CH_2_Si)_2_ and (CH_3_)_2_SiCH_2_CH_2_CH_2_NH), 1.34–1.46, (m, 24H, overlapping of signals, SiCH_2_CH_2_CH_2_Si and CH_3_Si(CH_2_CH_2_CH_2_Si)_2_), 1.49–1.62 (m, 16H, (CH_3_)_2_SiCH_2_CH_2_CH_2_NH), 3.27 (m, 16H, (CH_3_)_2_SiCH_2_CH_2_CH_2_NH), 6.40 (d, 8H, ^3^J_(H-H)_ = 15.7 Hz, PhCH=CH(CO)NH), 6.77 (d, 8H, ^3^J_(H-H)_ = 8.1 Hz, 1H_Ar_, *meta*-CH=CH), 6.91 (d, 8H, ^3^J_(H-H)_ = 8.1 Hz, 1H_Ar_, *ortho*-CH=CH), 7.03 (s, 8H, 1H_Ar,_ *ortho*-CH=CH, *ortho*-OH), 7.41 (d, 8H, ^3^J_(H-H)_ = 15.7 Hz, PhCH=CH(CO)NH). ^13^C-NMR (CD_3_OD): δ (ppm) −4.2 ((CH_3_)_2_SiCH_2_CH_2_CH_2_NH), −2.9 (CH_3_Si(CH_2_CH_2_CH_2_Si)_2_), 13.7 (-(CH_3_)_2_SiCH_2_CH_2_CH_2_NH), 19.8, 20.0, 20.1, 21.1 (SiCH_2_CH_2_CH_2_Si and -CH_3_Si(CH_2_CH_2_CH_2_Si)_2_), 25.2 (CH_3_)_2_SiCH_2_CH_2_CH_2_NH), 44.0 ((CH_3_)_2_SiCH_2_CH_2_CH_2_NH), 115.2 (CAr, *ortho*-OH, *ortho*-CH=CH), 116.5 (CAr, *meta*-CH=CH), 118.6 (PhCH=CH(CO)NH), 122.1, (CAr, *ortho*-CH=CH), 128.4 (Cipso, *meta*-OH, *para*-OH), 142.1 (PhCH=CH(CO)NH), 146.7 (Cipso), 148.7 (Cipso), 169.2 (NHC=O). {^1^H-^15^N}-HMBC-NMR (CD_3_OD): δ (ppm) −259.6 (NHC=O). Elemental Analysis (%): Calc for C_152_H_244_N_8_O_24_Si_13_ (2932.76 g/mol). C, 62.25; H, 8.39; N, 3.82. Exp.: C, 60.60; H, 8.49; N, 4.89.

#### 2.1.6. Synthesis of G_2_-[Si(CH_2_)_3_NH(CO)Ph(OH)_3_]_8_ (**6**)

Dendrimer **6** has been prepared through the same method as described for **1** by using the following reagents: gallic acid (385.4 mg, 2.049 mmol), EDCI·HCl (391.9 mg, 2.049 mmol), HOBt (272.7 mg, 2.049 mmol), G_2_-[Si(CH_2_)_3_NH_2_]_8_ (352.0 mg, 0.213 mmol) and NEt_3_ (1.704 mmol). Compound **6** was obtained as a brown solid (455.7 mg, 75%). ^1^H-NMR (CD_3_OD): δ (ppm) −0.07 (s, 12H, CH_3_(CH_2_CH_2_CH_2_Si)_2_), −0.03 (s, 48H, -(CH_3_)_2_SiCH_2_CH_2_CH_2_NH), 0.45–0.68 (m, overlapping of signals, 64H, -SiCH_2_CH_2_CH_2_Si, CH_3_Si(CH_2_CH_2_CH_2_Si)_2_ and (CH_3_)_2_SiCH_2_CH_2_CH_2_NH), 1.31–1.44, (m, 24H, overlapping of signals, SiCH_2_CH_2_CH_2_Si and CH_3_Si(CH_2_CH_2_CH_2_Si)_2_), 1.51–1.65 (m, 16H, (CH_3_)_2_SiCH_2_CH_2_CH_2_NH), 3.27 (m, 16H, (CH_3_)_2_SiCH_2_CH_2_CH_2_NH), 6.85 (s, 16H), 7.03 (broad s, overlapping of signals, 32H, NH and Ph(OH)). ^13^C-NMR (CD_3_OD): δ (ppm). −4.3 ((CH_3_)_2_SiCH_2_CH_2_CH_2_NH), −2.9 (CH_3_Si(CH_2_CH_2_CH_2_Si)_2_), 13.7 ((CH_3_)_2_SiCH_2_CH_2_CH_2_NH), 19.8, 19.9, 20.1, 20.2 (SiCH_2_CH_2_CH_2_Si and -CH_3_Si(CH_2_CH_2_CH_2_Si)_2_), 25.3 ((CH_3_)_2_SiCH_2_CH_2_CH_2_NH), 44.3 ((CH_3_)_2_SiCH_2_CH_2_CH_2_NH), 107.8 (CAr, *ortho*-OH), 126.4 (Cipso, *para*-OH), 146.6 (Cipso, *ortho*-OH), 170.4 (NHC=O). {^1^H-^15^N}-HMBC-NMR (CD_3_OD): δ (ppm) −266.6 (NHC=O). Elemental Analysis (%): Calc for C_136_H_228_N_8_O_32_Si_13_ (2852.45 g/mol). C, 57.27; H, 8.06; N, 3.93. Exp.: C, 56.77; H, 8.98; N, 4.79.

### 2.2. Spectrophotometric Studies of the Antioxidant Capacity

#### 2.2.1. DPPH Free Radical-Scavenging Activity

This method involves the radical concentration reduction by the antioxidant agent, which entails absorbance reduction and increasing compound concentration. When antioxidants are mixed with DPPH solution, the color turns from purple to yellow, with the maximum absorption at 530 nm. The ability of dendritic polyphenols to scavenge the DPPH radical was evaluated. An aliquot of 100 µL of different stock sample concentrations in the range of 0.01 and 100 µM in methanol was added to a volume of 900 µL of a DPPH stock solution (0.12 mM) in a DMSO/H_2_O (1:1) mixture. The mixture was shaken, kept in the dark at room temperature, and recorded at 530 nm every 5 min up to 30 min. The blank was constituted by methanol instead of the sample. 

#### 2.2.2. FRAP Assay

The FRAP method is based on the reduction at an acidic pH of tripyridyltriazine (Fe^III^‒TPTZ) complex to the ferrous (Fe^II^) form, developing an intense blue color with maximum absorption at 593 nm [21]. The ability of polyphenolic dendrimers to reduce Fe(III) to Fe(II) was evaluated. An aliquot of 20 µL of different stock sample concentrations in the range of 0.01 and 100 µM in methanol was added to a volume of 980 µL of a FRAP stock solution in acetate buffer. The mixture was shaken, kept in the dark at room temperature, and recorded at 593 nm every 5 min up to 30 min. The blank constituted by methanol instead of the sample.

Preparation of the FRAP stock. Three solutions of TPTZ (10 mM solution in 40 mM of hydrochloric acid), FeCl_3_ (20 mM solution in ACS water), and acetate buffer (20 mM in 100 mL of ACS water, pH 3.6) were mixed in a 1:1:10 ratio.

#### 2.2.3. Analytical Evaluation: Estimation of the IC_50_ or EC_50_ and Trolox Equivalent Antioxidant Capacity (TEAC)

The IC_50_ of DPPH scavenging activity and EC_50_ of FRAP capacities were calculated following similar protocols. Firstly, for each concentration, it was necessary to calculate the DPPH remnant, as well as the Fe(II) that formed, through the following equations:

$$1.\;{\mathrm{DPPH}}_{\mathrm{REM}}\left(\%\right)=\left(\frac{\mathrm{As}}{\mathrm{Ac}}\right)\times 100\;\;\;\;\;\;2.\;\mathrm{Fe}(\mathrm{II}{)}_{\mathrm{FOR}}\left(\%\right)=\left(\frac{\mathrm{As}-\mathrm{Ac}}{\mathrm{Ac}}\right)\times 100$$

where REM = remnant, FOR = formed, As = Sample absorbance, and Ac = Control absorbance. The graphical representation of the percentage of the remnant or formed reagent versus the −log[X] (X = sample concentration) gives the concentration that produces 50% antioxidant activity. This determination was carried out through GraphPad Prism 8. The results are expressed as mean ± SEM and are representative of at least three independent experiments.

TEAC: For the standard Trolox, the linear regression was carried out in the same way as the samples, with a range of concentrations between 5 and 40 μM for DPPH and FRAP (see Figure S42A). The concentration at which each compound reached maximum activity was calculated through the intersection point with the line of the equation obtained from the data before and after the steady state (see Figure S42B). The interpolation of the absorbance where the maximum activity was reached in the Trolox standard calibration curve gave us the corresponding Trolox concentration. Afterward, to express the results as micromole of Trolox per micromole of compound or polyphenol, the Trolox concentration, obtained through interpolation, was divided by the dendrimer or polyphenol concentration corresponding to the maximum activity. The results are expressed as mean ± SEM and are representative of at least three independent experiments.

### 2.3. Electrochemical Measurements

Electrochemical measurements were carried out on an Autolab PGSTAT 12 potentiostat from Metrohm (Utrecht, The Netherlands), employing a glassy carbon (GC) disk working electrode. The GC electrode was polished after each measurement using a 0.1 and 0.05 µm alumina/water slurry until a shiny mirror-like finish was achieved, and washed with ultrapure water. Electrodes were modified with 1 mM solutions of each compound (L, G_1,_ and G_2_) in MeOH; 5 µL drops were drop-cast in the electrode and allowed to dry before the measurements. Electrochemical measurements were performed in a 20 mM acetate buffer solution (pH 3.6), employing an Ag|AgCl|KCl (3M) reference electrode and a platinum wire as a counterelectrode.

### 2.4. Antibacterial Activity

In vitro antibacterial analysis of dendritic polyphenols was performed following the international standard method ISO 20776-1:2006 [22]. The antibacterial activity was assayed in two different bacteria, *Escherichia coli* (CECT 515) and *Staphylococcus aureus* (CECT 240), both obtained from the Spanish Type Culture Collection in lyophilized form. The biocide solutions were prepared following the EUCAST (European Committee for Antimicrobial Susceptibility Testing) protocol for hydrophobic compounds. For this purpose, stock solutions in DMSO of each compound were made and then, subsequent dilutions in Mueller-Hinton agar (Scharlau, ref. 02–136) were developed to obtain the testing concentrations (from 0.0312 ppm to 16 ppm) with 1% of DMSO. The assay was carried out in 96-well plates, with two different wells for each concentration and with different controls (biocide, inoculum, culture medium, and DMSO). The bacteria were inoculated at a concentration of 10^5^ CFU/mL in wells. After 24 h of incubation with biocides at 37 °C, the increase of turbidity at 625 nm was determined using an Ultra Microplate reader (BIO-TECK Instruments, model ELx808). MIC_50_ and MIC_80_ values were the concentrations of the compounds with an optical density of 50% and 20% compared to nontreated bacteria.

### 2.5. Cell Viability

HFF-1 cells were grown routinely in DMEM (Dulbecco’s modified Eagle’s medium, Sigma Aldrich, St. Louis, MO, USA, ref. D6429) supplemented with 10% fetal bovine serum (FBS, Sigma Aldrich, ref. F7524) and 1% penicillin/streptomycin/amphotericin B (Sigma Aldrich, ref. A5955) at 37 °C and 5% CO_2_. Cells were seeded in 96-well plates (Nunclon Delta Surface, Thermo Fischer Scientific, Waltham, MA, USA; 8 × 10^3^ cells per well) and grown in a complete DMEM medium for 24 h. Cell viability was determined by MTT assay (MTT, 3-(4,5-dimethyl-2-thiazolyl)-2,5-diphenyl-2H-tetrazolium bromide). A stock compound solution was prepared in DMSO. Then, dilutions in cell culture were made to obtain the final concentrations with 1% of DMSO per well. Cells were treated with the MIC_50_ and MIC_80_ concentrations obtained for *S. aureus*, then incubated at 37 °C with 5% CO_2_ for 24 h. After incubation, MTT was added at a concentration of 5 mg/mL and plates were incubated for 3 h. Finally, the culture medium was removed and the purple formazan crystals formed by the mitochondrial dehydrogenase and reductase activity of vital cells were dissolved in DMSO. The optical density, directly proportional to the number of surviving cells, was quantified at 570/630 nm in a spectrophotometer BioTEK 800 (Izasa). The assay was carried out in triplicate, with eight different wells for each concentration and with different controls (culture medium and DMSO).

## 3. Results and Discussion

### 3.1. Synthesis and Characterization of Polyphenolic Dendrimers

**Scheme 1:**
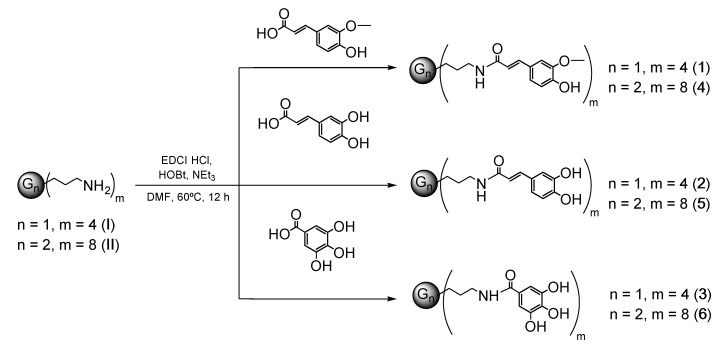
Synthesis of polyphenolic dendrimers (**1**–**6**).

**Figure 1:**
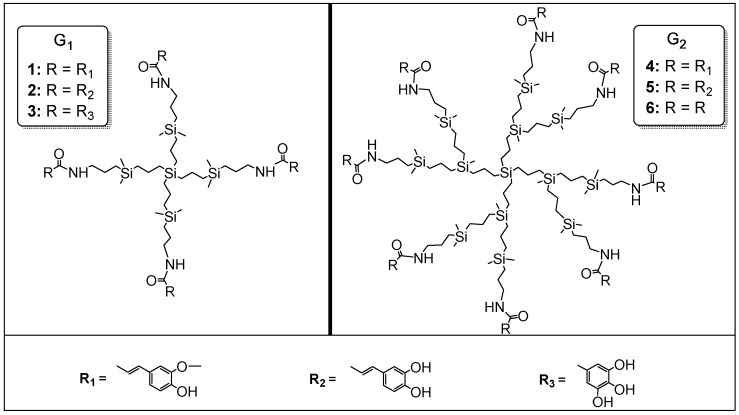
Proposed structures for polyphenolic carbosilane dendrimers (**1**–**6**).

Three families of polyphenolic dendritic compounds G_n_-[Si(CH_2_)_3_NHC(O)R]_m_ (R_1_ = ferulic: n = 1, m = 4,(**1**), n = 2, m = 8 (**4**); R_2_ = caffeic: n = 1, m = 4,(**2**), n = 2, m = 8 (**5**); R_3_ = gallic: n = 1, m = 4,(**3**), n = 2, m = 8 (**6**)) (Figure 1) were prepared using carbosilane dendrimers with amino groups on the periphery G_n_-[Si(CH_2_)_3_NH_2_]_m_ (n = 1, m = 4 **(I)**, n = 2, m = 8 **(II)**) as precursors [23]. The presence of carboxylic groups in selected polyphenols allows for easy attachment via a covalent bond to the amino groups localized at the surface of the dendrimers through a straightforward amidation reaction. The synthetic procedure is shown in Scheme 1. The reaction between the dendritic precursors with amine groups and different acids was carried out using N-(3-Dimethylaminopropyl)-N′-ethylcarbodiimide hydrochloride (EDCI·HCl) and 1-Hydroxybenzotriazole (HOBt) as coupling reagents [24]. After purification by size exclusion chromatography, polyphenolic carbosilane dendrimers were obtained as brown solids in moderate yields.

**Figure 2:**
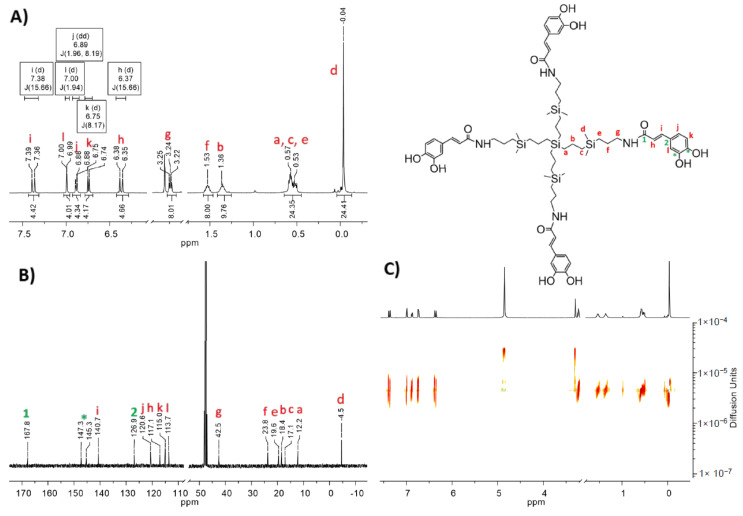
NMR spectra in CD_3_OD of compound G_1_-[Si(CH_2_)_3_NH(CO)CH=CHCH_2_Ph(OH)_2_)]_4_ (**2**): (**A**) ^1^H-NMR, (**B**) ^13^C-NMR, and (**C**) ^1^H-DOSY-2D-NMR (d, doublet; dd, doublet of doublets).

The ^1^H-NMR, ^13^C-NMR, ^1^H-DOSY-2D-NMR spectra confirmed the formation of new polyphenolic dendritic families. The NMR spectra of the first-generation polyphenolic dendrimer functionalized with caffeic acid (**2**) are reported in Figure 2 as an example.

^1^H-NMR shows standard signals for all polyphenolic dendrimers (**1**–**6**) corresponding to the carbosilane skeleton in the range from 0 to 1.60 ppm, as well as a signal located around 3.30 ppm due to the protons of the methylene group being bonded to the amide nitrogen. For the ferulic and caffeic derivatives, two signals around 6.40 and 7.50 ppm assigned to the protons of alkene fragment and a set of signals corresponding to the aromatic protons approximately at 6.80, 7.00, and 7.10 ppm were observed, respectively. In the case of ferulic counterparts, an additional signal attributed to the methoxy group appears at 3.90 ppm. Furthermore, the gallic carbosilane dendrimers show a signal corresponding to the aromatic protons at 6.90 ppm and a set of signals in the range of 7.20‒7.30 ppm corresponding to the protons present in the amide and hydroxyl groups. Moreover, the ^13^C-NMR spectra exhibit a group of signals placed between −5.0 and 26.0 ppm, corresponding to the carbosilane backbone, and the signal of the carbon present in the methylene group bounded to the amide at 44.0 ppm.

Moreover, all polyphenolic dendrimers show a set of aromatic carbons signals between 107.0 and 150.0 ppm, corresponding to the carbon of the carbonyl group at 170.0 ppm. In the case of the ferulic and caffeic derivatives, two signals corresponding to the alkene fragment appear at around 119.0 and 142.0 ppm. In the case of the ferulic derivate, a signal corresponding to the carbon of the methoxyl group appears at 56.4 ppm. In the {^1^H-^15^N}-HMBC-NMR, a signal attributed to the nitrogen of the amide group was observed at −259.0 ppm for the ferulic and caffeic dendrimers and −266.0 ppm for the gallic counterparts.

^1^H-DOSY-2D-NMR experiments were performed, allowing us to confirm the presence and purity of the dendritic polyphenols. The ESI spectra confirmed the proposed structure for the first-generation dendrimers, showing that the surface of dendrimers is fully substituted with the polyphenolic unit. Unfortunately, the molecular peak for the dendrimers of the second generation with eight surface groups was not observed.

Finally, stability assays were performed at different pH values using ^1^H-NMR, and we observed that the compounds were stable for 24 h under physiological (pH = 7.4) and acid (pH = 3.6) conditions.

### 3.2. Antioxidant Capacity Evaluation

In the literature, there are several proposals for the antioxidant mechanisms by which polyphenols decrease ROS levels both in vitro and in vivo [25,26]. Generally, once the radicals are formed, polyphenols can trap and dissipate the free electrons of the radicals such as ^•^OH, O_2_^•−^, NO^•^, or OONO^−^, preventing the formation of more reactive ROS harmful to the cells. However, changes in the polyphenol structure can produce disturbances in their antioxidant activity. The anchoring of selected polyphenols to the carbosilane skeleton modifies the polyphenol structure, necessitating the antioxidant evaluation of the new compounds.

Due to the high complexity of the antioxidant activity mechanisms, it is necessary to use more than one chemical method [27]. In this study, three different techniques have been employed: (i) a DPPH radical scavenging assay based on single-electron transference (SET) and hydrogen atom transference (HAT), (ii) a FRAP assay, which involves the use of a metal complex and is based only on an electron transfer reaction (SET) [28], and (iii) electrochemical assays of cyclic voltammetry (CV).

**Figure 3:**
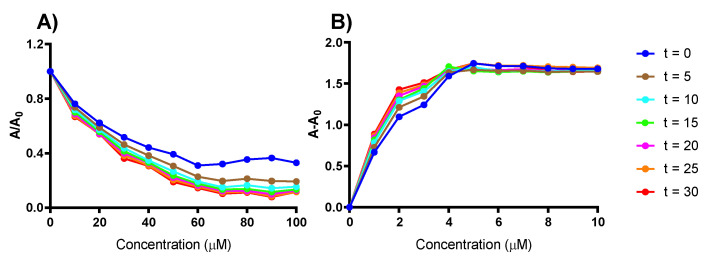
Study of the antioxidant activities in a wide range of concentrations and times (t). (**A**) Free ferulic acid, evaluated by DPPH assay; (**B**) compound G_1_-[Si(CH_2_)_3_NH(CO)Ph(OH)_3_]_4_ (3), evaluated by FRAP assay.

***Spectrophotometric studies of the antioxidant capacity.*** In both the DPPH and FRAP experiments, different dendrimer concentrations have been tested until a steady state was reached. Additionally, different reaction times were used; at 30 min the maximum antioxidant activity in each concentration was reached for all compounds (Figure 3).

**Figure 4:**
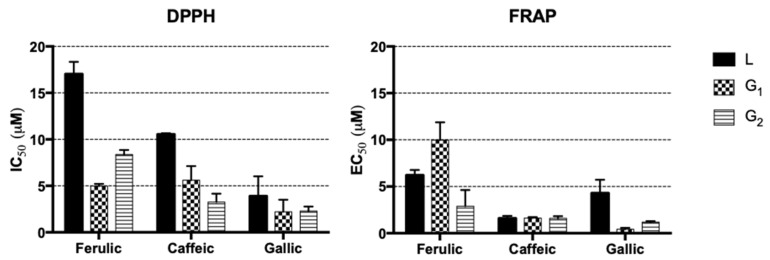
Antioxidant activity of polyphenolic dendrimers (**1**–**6**). Mean values ± SEM (standard error of the mean), representative of at least three independent experiments, are shown.

The interpretation of the results involved the evaluation of the IC_50_ (concentration of antioxidant dendrimer that can scavenge 50% of free radical DPPH) and EC_50_ (concentration of antioxidant dendrimer that can increase 50% of FRAP capacity), as well as the Trolox equivalent antioxidant capacity (TEAC). The lowest value of IC_50_ or EC_50_ means the highest antioxidant activity. According to the data obtained from the DPPH assay, it is possible to establish a correlation between the number of hydroxyl groups and the antioxidant activity, with the gallic counterparts being the most active, probably due to the presence of two electron-donating hydroxyl substituents in ortho position to the phenolic group. The better activity observed for the caffeic dendrimers in comparison with the ferulic counterparts could be attributed to the hydroxyl groups in the ortho position in the catechol ring, which, according to the literature, decrease the O‒H bond dissociation enthalpy and, as a consequence, the rate of H-atom transfer to peroxyl radicals increases [29]. Moreover, the multivalence of dendrimers seems to improve the antioxidant activity compared to free polyphenols (L). However, the first-generation dendrimer seems to be sufficient, since doubling the number of polyphenolic groups in the second generation does not significantly improve the results (Figure 4), probably due to the steric hindrance [28]. Compared with similar dendrimers with a polyether skeleton functionalized with a precursor of ferulic acid (vanillin), it is possible to observe that the radical scavenging found for these systems with eight functional groups (an IC_50_ value of around 5 µM in DPPH assay) showed the same radical scavenging activity as compound **2** with half the number of polyphenolic groups [12].

Surprisingly, the antioxidant behavior in the case of free polyphenols (L) obtained by FRAP is quite different and caffeic acid stands out as the best antioxidant agent (Figure 4). Nevertheless, regarding polyphenolic dendrimers, it is only possible to observe that the first-generation dendrimer functionalized with gallic acid improves the antioxidant activity compared to free polyphenols (L). However, in several cases, the FRAP method does not show a correlation with other antioxidant techniques [28].

**Figure 5:**
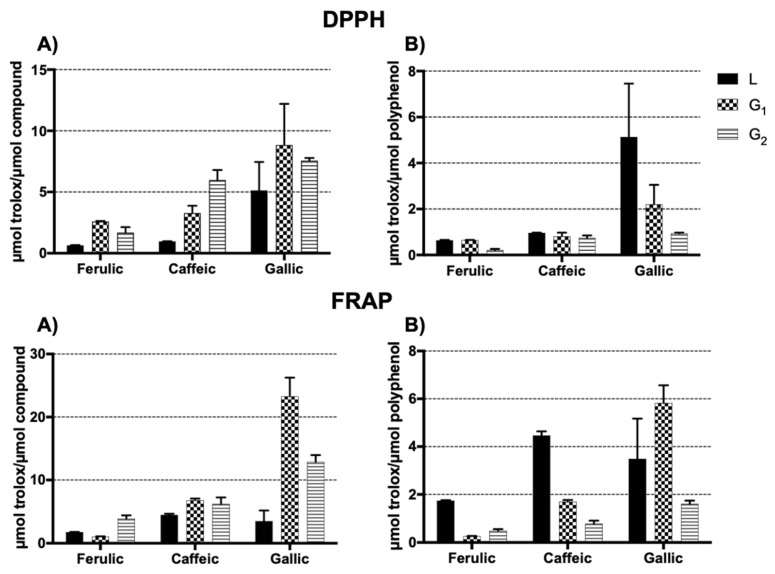
Results of antioxidant activity of compound **1**–**6**, expressed in Trolox units for DPPH and FRAP assays: (**A**) micromole of Trolox per micromole of the compound; or (**B**) micromole of polyphenol. Mean values ± SEM, representative of at least three independent experiments, are shown.

In the interest of having a different but complementary interpretation of the results, the TEAC was determined. Results were expressed by (i) micromole of Trolox per micromole of compound (Figure 5A) or (ii) per micromole of polyphenol present in the dendrimer (Figure 5B), to track the correlation between the number of polyphenols in the skeleton and the antioxidant activity. Regarding the study focused on dendritic molecules, it is possible to observe the same tendency of antioxidant activity concerning the number of hydroxyl groups present in polyphenol moieties, as previously observed for IC_50_ and EC_50_. Once again, gallic acid compounds are the most promising ones (Figure 5A). However, taking into account the number of polyphenolic groups, the results depend on the antioxidant assay. In the case of DPPH, the binding of polyphenol to the dendritic structure did not improve the antioxidant activity of free polyphenols. However, although this behavior seems to be similar for ferulic and caffeic in the case of FRAP assays, different results were obtained for gallic acid, where a first-generation dendrimer functionalized with gallic acid was the most active (Figure 5B).

***Electrochemical characterization of the antioxidant capacity:*** The electrochemical behavior of the synthesized polyphenolic dendrimers (**1**–**6**), together with the free polyphenol (L), has been investigated using cyclic voltammetry (CV). CV is a powerful electrochemical technique that can be employed to study the ability of a molecule to undergo electron transfer and to estimate the antioxidant activity through the peak potential (E_p_), intensity (i_p_), and reversibility (ΔE_p_). A lowering in the E_p_ or ΔE_p_ and an increase in the i_p_ are indicative of higher antioxidant activity [30,31].

**Figure 6:**
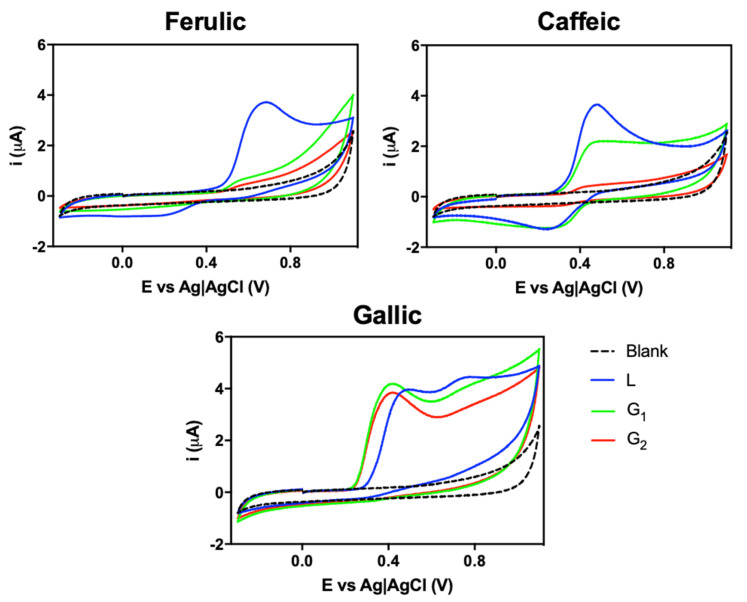
Cyclic voltammograms of polyphenols and their dendritic derivatives (**1**–**6**). For more experimental details, see Section 2.3.

Figure 6 shows the cyclic voltammograms of the different polyphenolic dendrimers (1‒6) compared with the blank signal and free polyphenol (L), while Table 1 collects the data extracted from the voltammograms. Interestingly, three different behaviors from these polyphenols and dendrimers derivatives were noticed.

For ferulic acid, a decrease in the E_p_ with an increase in the dendrimer generation were observed (73 mV for G_2_ compared to L), together with a dramatic decrease in the i_p_. Being an irreversible electrochemical reaction, ΔE_p_ could not be evaluated. In the case of caffeic acid, the difference between the anodic and cathodic peak (ΔE_p_) was appreciably decreased (by 85 mV for G_2_ compared to L). This decrease was more pronounced when the dendrimer generation increased. This fact was indicative of a faster electronic transfer between the redox site of the dendrimer (polyphenol derivative) and the electrode surface [32]. The decreasing trend in i_p_ for ferulic and caffeic acids with the increase in dendrimer generation can be explained by the lower availability of the electroactive groups due to steric effects that hinder the electronic transfer. This result is in agreement with works using dendrimers modified with ferrocene derivatives as an electroactive moiety [33,34]. Gallic acid shows a decrease in the E_p_ (68 mV for G_2_ compared to L), demonstrating a higher antioxidant capacity again in the dendritic compounds versus the free polyphenol. The general trend observed in terms of the decrease in E_p_ can be explained by the electron-donating effect of the dendritic core substituent in the aromatic group, facilitating the electronic transfer [35]. Interestingly, the first- and second-generation caffeic acid dendrimer derivatives do not show remarkable differences with the free polyphenol in terms of i_p_. In contrast to the ferulic and caffeic acid derivatives, the gallic acid dendritic derivatives maintained a comparable peak intensity, together with a significant decrease in E_p_. These data confirm the improved antioxidant activity of gallic acid dendritic derivatives versus the free polyphenol, proving that they are the most promising compounds from the previous antioxidant assays.

### 3.3. Antibacterial Activity and Viability Assays in HFF-1 Cells

As mentioned, polyphenols exhibit, in addition to excellent antioxidant properties, antibacterial activity. These properties could determine a cosmetic application of new polyphenolic dendritic derivates by their positive effects on the skin, preventing microbial infections. The antibacterial activity of polyphenolic compounds was determined in *Staphylococcus aureus* and *Escherichia coli* strains as models of Gram-positive (+) and Gram-negative (−) bacteria, respectively. The minimum inhibitory concentration required to inhibit 50% (MIC_50_) and 80% (MIC_80_) of the bacterial growth was determined for the dendritic compounds (**1**–**6**) as well as for the free polyphenol (L); all data are collected in Table 2. Due to the predominantly hydrophobic nature of the generated dendritic systems, **1**–**6**, it was only possible to measure concentrations up to 16 mg/L. Free polyphenols were measured at the same concentration range to determine the effect on the use of dendrimers as anchorage platforms of polyphenols as antibacterial agents. In the case of *S. aureus*, the results showed that first-generation dendrimers functionalized with caffeic and gallic acids were the most active, and the gallic derivate stands out as the most promising with a MIC_50_ of 4 ppm. Despite the presence of the double polyphenolic groups in second-generation dendrimers, only the gallic counterpart presents activity, but at higher concentrations than first-generation ones. This behavior has been manifested in other dendritic systems previously obtained in our research group [36]. In the case of ferulic derivatives as well as free polyphenols, no activity has been observed. Regarding *E. coli*, again derivate **4** (G_1_—Gallic) stood out with an MIC_50_ value of 16 ppm—it was the only one to present significant activity.

Because of the obtained results, it is verified that the anchorage of polyphenols on the skeleton provides a bacteriostatic effect. This behavior could be related to the antioxidant activity of the polyphenolic dendrimers, observing that an increase in the antioxidant activity leads to an improvement in the antibacterial capacity.

Finally, in the pursuit of determining the toxicity of the dendritic polyphenols at the concentration that they showed antibacterial activity, viability assays in healthy cells was performed. For this purpose, Neonatal Human Foreskin fibroblasts (HFF-1) were exposed to the established antibacterial concentrations for each system (MIC_80_ and MIC_50_). We observed that cell viability was not reduced after treatment with the polyphenolic dendrimers (Table 2). These preliminary results open the door for future applications in the cosmetic field.

## 4. Conclusions

A new family of carbosilane dendrimers functionalized with ferulic, caffeic, and gallic acids has been obtained through a simple and easy protocol based on amidation reactions. The antioxidant activity of these polyphenolic dendrimers was determined. We observed that the use of the dendritic systems as anchorage platforms for polyphenolic compounds improved their antioxidant capacity, highlighting the first-generation dendrimer functionalized with gallic acid.

Moreover, antibacterial activity assays showed that first-generation dendrimers functionalized with caffeic and gallic acids were able to inhibit the bacterial growth of *S. aureus*, while free acids did not. Furthermore, the gallic carbosilane dendrimer of the first generation was able to inhibit *E. coli* growth, standing out once again as the most active.

Furthermore, it is worth stressing that the viability of healthy HFF-1 cells was not affected by treatment with polyphenolic dendrimers. All the obtained results point to the potential for their future application as antioxidant compounds with antibacterial capacity in the cosmetics field.

## Figures and Tables

**Table 1:** Peak data extracted from the CVs of Figure 6. Error expressed as a standard deviation of three independent measurements.

Polyphenols	N° Polyphenols	Compound	E_p_ (V)	I_p_ (µA)	ΔE_p_ (V)
Ferulic	1	L	0.686 ± 0.009	3.70 ± 0.08	-
	4	G_1_ (**1**)	0.637 ± 0.002	0.75 ± 0.06	-
	8	G_2_ (**4**)	0.613 ± 0.008	0.56 ± 0.02	-
Caffeic	1	L	0.263 ± 0.005	2.91 ± 0.07	0.205 ± 0.002
	4	G_1_ (**2**)	0.276 ± 0.009	1.39 ± 0.04	0.188 ± 0.006
	8	G_2_ (**5**)	0.302 ± 0.010	0.143 ± 0.09	0.120 ± 0.009
Gallic	1	L	0.477 ± 0.001	3.87 ± 0.08	-
	4	G_1_ (**3**)	0.410 ± 0.008	4.12 ± 0.07	-
	8	G_2_ (**6**)	0.409 ± 0.008	3.40 ± 0.40	-

**Table 2:** Bacteriostatic minimum inhibitory concentration (MIC) in *S. aureus* and *E. coli* and percentage of HFF-1 cells’ viability obtained by treatment with **1**–**6**.

Polyphenols	N° Polyphenols	Compound	*S. aureus*		*E. coli*	HFF-1	
			MIC_80_ [ppm]	MIC_50_ [ppm]	MIC_50_ [ppm]	[ppm]	% Viability
**Ferulic**	1	L	>16	>16	>16	-	-
	4	G_1_ (**1**)	>16	>16	>16	16	95.4 ± 6.6
	8	G_2_ (**4**)	>16	>16	>16	16	92.9 ± 7.4
**Caffeic**	1	L	>16	>16	>16	-	-
	4	G_1_ (**2**)	16	8	>16	16	100.0 ± 0.0
						8	98.8 ± 1.7
	8	G_2_ (**5**)	>16	>16	>16	16	100.0 ± 0.0
**Gallic**	1	L	>16	>16	>16	-	-
	4	G_1_ (**3**)	8	4	16	8	99.1 ± 1.0
						4	84.8 ± 7.5
	8	G_2_ (**6**)	16	16	>16	16	100.0 ± 0.0

## Supplementary Materials

The following are available online at https://www.mdpi.com/1999-4923/12/8/698/s1, Figure S1. Mass Spectrometry (ESI-TOF) of dendritic polyphenol (1). Figure S2. ^1^H-NMR (500 MHz, CD_3_OD) of dendritic polyphenol (1). Figure S3. ^13^C-NMR (500 MHz, CD_3_OD) of dendritic polyphenol (1). Figure S4. {^1^H-^15^N}-HMBC-NMR (500 MHz, CD_3_OD) of dendritic polyphenol (1). Figure S5. ^1^H-DOSY-2D-NMR (500 MHz, CD_3_OD) of dendritic polyphenol (1). Figure S6. {^1^H-^1^H}-COSY-2D-NMR (500 MHz, CD_3_OD) of dendritic polyphenol (1). Figure S7. {^1^H-^13^C}-HSQC-2D-NMR (500 MHz, CD_3_OD) of dendritic polyphenol (1). Figure S8. {^1^H-^13^C}-HMBC-2D-NMR (500 MHz, CD_3_OD) of dendritic polyphenol (1). Figure S9. Mass Spectrometry (ESI-TOF) of dendritic polyphenol (2). Figure S10. ^1^H-NMR (500 MHz, CD_3_OD) of dendritic polyphenol (2). Figure S11. ^13^C-NMR (500 MHz, CD_3_OD) of dendritic polyphenol (2). Figure S12. {^1^H-^15^N}-HMBC-NMR (500 MHz, CD_3_OD) of dendritic polyphenol (2). Figure S13. ^1^H-DOSY-2D-NMR (500 MHz, CD_3_OD) of dendritic polyphenol (2). Figure S14. {^1^H-^1^H}-COSY-2D-NMR (500 MHz, CD_3_OD) of dendritic polyphenol (2). Figure S15. {^1^H-^13^C}-HSQC-2D-NMR (500 MHz, CD_3_OD) of dendritic polyphenol (2). Figure S16. Mass Spectrometry (ESI-TOF) of dendritic polyphenol (3). Figure S17. ^1^H-NMR (500 MHz, CD_3_OD) of dendritic polyphenol (3). Figure S18. ^13^C-NMR (500 MHz, CD_3_OD) of dendritic polyphenol (3). Figure S19. {^1^H-^15^N}-HMBC-NMR (500 MHz, CD_3_OD) of dendritic polyphenol (3). Figure S20. ^1^H-DOSY-2D-NMR (500 MHz, CD_3_OD) of dendritic polyphenol (3). Figure S21. {^1^H-^1^H}-COSY-2D-NMR (500 MHz, CD_3_OD) of dendritic polyphenol (3). Figure S22. {^1^H-^13^C}-HSQC-2D-NMR (500 MHz, CD_3_OD) of dendritic polyphenol (3). Figure S23. ^1^H-NMR (500 MHz, CD_3_OD) of dendritic polyphenol (4). Figure S24. ^13^C-NMR (500 MHz, CD_3_OD) of dendritic polyphenol (4). Figure S25. {^1^H-^15^N}-HMBC-NMR (500 MHz, CD_3_OD) of dendritic polyphenol (4). Figure S26. ^1^H-DOSY-2D-NMR (500 MHz, CD_3_OD) of dendritic polyphenol (4). Figure S27. {^1^H-^1^H}-COSY-2D-NMR (500 MHz, CD_3_OD) of dendritic polyphenol (4). Figure S28. {^1^H-^13^C}-HSQC-2D-NMR (500 MHz, CD_3_OD) of dendritic polyphenol (4). Figure S29. {^1^H-^13^C}-HMBC-2D-NMR (500 MHz, CD_3_OD) of dendritic polyphenol (4). Figure S30. ^1^H-NMR (500 MHz, CD_3_OD) of dendritic polyphenol (5). Figure S31. ^13^C-NMR (500 MHz, CD_3_OD) of dendritic polyphenol (5). Figure S32. {^1^H-^15^N}-HMBC-NMR (500 MHz, CD_3_OD) of dendritic polyphenol (5). Figure S33. ^1^H-DOSY-2D-NMR (500 MHz, CD_3_OD) of dendritic polyphenol (5). Figure S34. {^1^H-^13^C}-HSQC-2D-NMR (500 MHz, CD_3_OD) of dendritic polyphenol (5). Figure S35. {^1^H-^13^C}-HMBC-2D-NMR (500 MHz, CD_3_OD) of dendritic polyphenol (5). Figure S36. ^1^H-NMR (500 MHz, CD_3_OD) of dendritic polyphenol (6). Figure S37. ^13^C-NMR (500 MHz, CD_3_OD) of dendritic polyphenol (6). Figure S38. {^1^H-^15^N}-HMBC-NMR (500 MHz, CD_3_OD) of dendritic polyphenol (6). Figure S39. ^1^H-DOSY-2D-NMR (500 MHz, CD_3_OD) of dendritic polyphenol (6). Figure S40. {^1^H-^1^H}-COSY-2D-NMR (500 MHz, CD_3_OD) of dendritic polyphenol (6). Figure S41. {^1^H-^13^C}-HSQC-2D-NMR (500 MHz, CD_3_OD) of dendritic polyphenol (6). Figure S42. (A) A representative calibration curve of inhibition of DPPH by Trolox standards. Representative results of at least three independent experiments are shown. (B) Graphics with equations line for compound G_1_-[Si(CH_2_)_3_NH(CO)Ph(OH)_3_]_4_ (3).
