# Erratum: Sanz del Olmo, N.; et al. Antioxidant and Antibacterial Properties of Carbosilane Dendrimers Functionalized with Polyphenolic Moieties. *Pharmaceutics* 2020, *12*, 698

**DOI:** 10.3390/pharmaceutics13010121

**Published:** 2021-01-19

**Authors:** Natalia Sanz del Olmo, Cornelia E. Peña González, Daniel Rojas, Rafael Gómez, Paula Ortega, Alberto Escarpa, Francisco Javier de la Mata

**Affiliations:** 1 Department of Organic and Inorganic Chemistry, and Research Institute in Chemistry “Andrés M. del Río” (IQAR), 28871 Alcalá de Henares, Spain; n.sanzdelolmo@gmail.com (N.S.d.O.); cornelia.pena@uah.es (C.E.P.G.); rafael.gomez@uah.es (R.G.); 2 Networking Research Center on Bioengineering, Biomaterials and Nanomedicine (CIBER-BBN), 28029 Madrid, Spain; 3 Instituto Ramon y Cajal de Investigacion Sanitaria, IRYCIS, Colmenar Viejo Road, Km 9, 100, 28034 Madrid, Spain; j.daniel.rojas.t@gmail.com (D.R.); alberto.escarpa@uah.es (A.E.); 4 Department of Analytical Chemistry, Physical Chemistry and Chemical Engineering, University of Alcalá, 28805 Madrid, Spain; 5 Faculty of Bioscience and Technology for Food, Agriculture and Environment University of Teramo, 64100 Teramo, Italy; 6 Institute in Chemistry “Andrés M. del Río” (IQAR), 28871 Alcalá de Henares, Spain

Due to an error during production, in Section 2.1, letters that were in italics refer to the H or C atom that corresponds to the chemical displacement in NMR have not been fully displayed. The corrected part should be:

## 2. Materials and Methods

### 2.1. Synthesis and Characterization of Polyphenolic Dendrimers (**1**–**6**)

All reactions were carried out under an inert atmosphere and solvents of reactions were bought in dry conditions. NMR experiments were carried out on a Varian 500 Hz instrument. Chemical shifts (δ) are given in ppm. As a reference, deuterated methanol solvent (CD_3_OD) was used. Assignment of resonances was done from HSQC, HMBC, and COSY NMR experiments. Elemental analyses were performed on a LECO CHNS-932 instrument. Mass spectra were obtained using the ESI-TOF technique from an Agilent 6210 TOF LC/MS instrument in MeOH, and ACN/H_2_O 0.1% formic acid as the mobile phase.

Spectra obtained through the different techniques for the new compounds described in this work are collected in the Supplementary Materials (Figures S1–S42).

#### 2.1.1. Synthesis of G_1_-[Si(CH_2_)_3_NH(CO)CH=CHCH_2_Ph(OH)(OCH_3_))]_4_ (**1**)

To achieve the synthesis of compound **1**, the activation of ferulic acid (179.0 mg, 0.922 mmol) with EDCI·HCl (176.7 mg, 0.922 mmol) and HOBt (124.6 mg, 0.922 mmol), using dry DMF as solvent for the reaction, was carried out in the first place. The mixture was stirred at room temperature for one hour. Afterwards, a DMF solution mixture of dendrimer functionalized with amine groups G_1_-[Si(CH_2_)_3_NH_2_]_4_ (127.0 mg, 0.192 mmol) and NEt_3_ (0.768 mmol) was added dropwise under stirring and at 0 °C. After five minutes in these conditions, the mixture was kept at 60 °C, overnight. The compound was purified by size exclusion chromatography in DMF, obtaining compound **1** as a brown solid (178.4 mg, 68%). ^1^H-NMR (CD_3_OD): δ (ppm) 0.00 (s, 24H, NHCH_2_CH_2_CH_2_Si(C*H*_3_)_2_), 0.51–0.67 (m, overlapping of signals, 24H, SiC*H*_2_CH_2_C*H*_2_Si and NHCH_2_CH_2_C*H*_2_Si(CH_3_)_2_), 1.39 (m, 8H, SiCH_2_C*H*_2_CH_2_Si), 1.57 (m, 8H, NHCH_2_C*H*_2_CH_2_Si(CH_3_)_2_), 3.29 (m, 8H, NHC*H*_2_CH_2_CH_2_Si(CH_3_)_2_), 3.88 (s, 12H, OC*H*_3_), 6.48 (d, 4H, ^3^J_(H-H)_ = 15.7 Hz, PhCH=C*H*(CO)NH), 6.81 (dd, 4H, ^3^J_(H-H)_ = 8.2 Hz, ^5^J_(H-H)_ = 1.8 Hz, 1H_Ar_, *ortho-OH*), 7.04 (d, 4H, ^3^J_(H-H)_ = 8.2 Hz, 1H_Ar_, *para-OCH_3_*), 7.12 (d, 4H, ^5^J_(H-H)_ = 1.8 Hz, 1H_Ar_, *ortho-OCH_3_*), 7.47 (d, 4H, ^3^J_(H-H)_ = 15.7 Hz, PhC*H*=CH(CO)NH). ^13^C-NMR (CD_3_OD): δ (ppm) −3.1 ((*C*H_3_)_2_SiCH_2_CH_2_CH_2_NH), 13.6 ((CH_3_)_2_Si*C*H_2_CH_2_CH_2_NH), 18.6, 19.9, 21.1 (Si*C*H_2_*C*H_2_*C*H_2_Si), 25.2 ((CH_3_)_2_SiCH_2_*C*H_2_CH_2_NH), 43.9 ((CH_3_)_2_SiCH_2_CH_2_*C*H_2_NH), 56.4 (OCH_3_), 111.6 (CAr, *ortho-OCH_3_*), 116.5 (CAr, *ortho-OH*), 118.9 (CAr, PhCH=*C*H(CO)NH), 123.2, (CAr, *para-OCH_3_*), 128.3 (Cipso, *para-OH*), 142.0 (Ph*C*H=CH(CO)NH*)*, 149.3 (Cipso), 149.8 (Cipso), 169.1 (NH*C*=O). {^1^H-^15^N}-HMBC-NMR (CD_3_OD): δ (ppm) −259.4 (*N*HC=O). MS: [M + H]^+^ = 1366.7266 u (Calc. 1366.7264 u), [M + Na]^+^ = 1388.7061 u (Calc. 1388.7063 u). Elemental Analysis (%): Calc for C_72_H_112_N_4_O_12_Si_5_ (1366.13 g/mol). C, 63.30; H, 8.26; N, 4.10. Exp.: C, 62.9; H, 8.26; N, 5.05.

#### 2.1.2. Synthesis of G_1_-[Si(CH_2_)_3_NH(CO)CH=CHCH_2_Ph(OH)_2_)]_4_ (**2**)

Dendrimer **2** was prepared through the same method as described for **1** by using the following reagents: caffeic acid (255.1 mg, 1.416 mmol), EDCI·HCl (271.4 mg, 1.416 mmol), HOBt (191.3 mg, 1.416 mmol), G_1_-[Si(CH_2_)_3_NH_2_]_2_ (195.3 mg, 0.295 mmol) and NEt_3_ (1.180 mmol). Compound **2** was obtained as a brown solid (231.9 mg, 61%). ^1^H-NMR (CD_3_OD): δ (ppm) −0.04 (s, 24H, NHCH_2_CH_2_CH_2_Si(C*H*_3_)_2_), 0.48–0.67 (m, overlapping of signals, 24H, SiC*H*_2_CH_2_C*H*_2_Si and NHCH_2_CH_2_C*H*_2_Si(CH_3_)_2_), 1.36 (m, 8H, SiCH_2_C*H*_2_CH_2_Si), 1.53 (m, 8H, NHCH_2_C*H*_2_CH_2_Si(CH_3_), 3.24 (m, 8H, NHC*H*_2_CH_2_CH_2_Si(CH_3_)_2_), 6.37 (d, 4H, ^3^J_(H-H)_ = 15.7 Hz, PhCH=C*H*(CO)NH), 6.75 (d, 4H, ^3^J_(H-H)_ = 8.2 Hz, 1H_Ar_, *meta-CH=CH*), 6.89 (dd, 4H, ^3^J_(H-H)_ = 8.2 Hz, ^5^J_(H-H)_ = 2.0 Hz, 1H_Ar_, *ortho-CH=CH*), 7.00 (d, 4H, ^5^J_(H-H)_ = 2.0 Hz, 1H_Ar_*, ortho-CH=CH, ortho-OH*), 7.38 (d, 4H, ^3^J_(H-H)_ = 15.7 Hz, PhC*H*=CH(CO)NH). ^13^C-NMR (CD_3_OD): δ (ppm) −3.1 ((*C*H_3_)_2_SiCH_2_CH_2_CH_2_NH), 13.6 ((CH_3_)_2_Si*C*H_2_CH_2_CH_2_NH), 18.5, 19.9, 21.0 (Si*C*H_2_*C*H_2_*C*H_2_Si), 25.2 ((CH_3_)_2_SiCH_2_*C*H_2_CH_2_NH), 43.9 ((CH_3_)_2_SiCH_2_CH_2_*C*H_2_NH), 115.1 (CAr, *ortho-OH, ortho-CH=CH*), 116.5 (CAr, *meta-CH=CH*), 118.5 (PhCH=*C*H(CO)NH), 122.1, (CAr, *ortho-CH=CH*), 128.3 (Cipso, *meta-OH, para-OH*), 142.1 (Ph*C*H=CH(CO)NH*)*, 146.7 (Cipso), 148.7 (Cipso), 169.2 (NH*C*=O). {^1^H-^15^N}-HMBC-NMR (CD_3_OD): δ (ppm) −259.5 (*N*HC=O). MS: [M + H]^+^ = 1310.6618 u (Calc. 1310.6670 u), [M + Na]^+^ = 1332.6431 u (Calc. 1332.6433 u). Elemental Analysis (%): Calc for C_68_H_104_N_4_O_12_Si_5_ (1310.02 g/mol). C, 62.35; H, 8.00; N, 4.28. Exp.: C, 60.23; H, 7.52; N, 4.50.

#### 2.1.3. Synthesis of G_1_-[Si(CH_2_)_3_NH(CO)Ph(OH)_3_]_4_ (**3**)

Dendrimer **3** was prepared through the same method as described for **1** by using the following reagents: gallic acid (252.9 mg, 1.344 mmol), EDCI·HCl (257.6 mg, 1.344 mmol), HOBt (181.6 mg, 1.344 mmol), G_1_-[Si(CH_2_)_3_NH_2_]_4_ (185.0 mg, 0.280 mmol) and NEt_3_ (1.120 mmol). Compound **3** was obtained as a brown solid (248.9 mg, 70%). ^1^H-NMR (CD_3_OD): δ (ppm) 0.00 (s, 24H, NHCH_2_CH_2_CH_2_Si(C*H*_3_)_2_), 0.46–0.71 (m, overlapping of signals, 24H, SiC*H*_2_CH_2_C*H*_2_Si and NHCH_2_CH_2_C*H*_2_Si(CH_3_)_2_), 1.40 (m, 8H, SiCH_2_C*H*_2_CH_2_Si), 1.59 (m, 8H, NHCH_2_C*H*_2_CH_2_Si(CH_3_), 3.31 (m, 8H, NHC*H*_2_CH_2_CH_2_Si(CH_3_)_2_), 6.88 (s, 8H, H_Ar_), 7.07–7.32 (m, overlapping of signals, 16H, NH and Ph(OH)). ^13^C-NMR (CD_3_OD): δ (ppm) −3.1 ((*C*H_3_)_2_SiCH_2_CH_2_CH_2_NH), 13.6 ((CH_3_)_2_Si*C*H_2_CH_2_CH_2_NH), 18.6, 19.9, 21.1 (Si*C*H_2_*C*H_2_*C*H_2_Si), 25.2 ((CH_3_)_2_SiCH_2_*C*H_2_CH_2_NH), 44.3 ((CH_3_)_2_SiCH_2_CH_2_*C*H_2_NH), 107.8 (CAr, *ortho-OH*), 126.4 (Cipso, *para-OH*), 146.6 (Cipso), 170.5 (NH*C*=O). {^1^H-^15^N}-HMBC-NMR (CD_3_OD): δ (ppm) −267.0 (*N*HC=O). MS: [M + H]^+^ = 1269.5762 u (Calc. 1269.5740 u). Elemental Analysis (%): Calc for C_60_H_96_N_4_O_16_Si_5_ (1269.87 g/mol). C, 56.75; H, 7.62; N, 4.41. Exp.: C, 56.60; H, 7.38; N, 4.95.

#### 2.1.4. Synthesis of G_2_-[Si(CH_2_)_3_NH(CO)CH=CHCH_2_Ph(OH)(OCH_3_))]_8_ (**4**)

Dendrimer **4** was prepared through the same method as described for **1** by using the following reagents: ferulic acid (259.5 mg, 1.336 mmol), EDCI·HCl (255.6 mg, 1.336 mmol), HOBt (180.6 mg, 1.366 mmol), G_2_-[Si(CH_2_)_3_NH_2_]_8_ (166.0 mg, 0.139 mmol) and NEt_3_ (1.112 mmol). Compound **4** was obtained as a brown solid (296.3 mg, 70%). ^1^H-NMR (CD_3_OD): δ (ppm) −0.03 (s, 12H, C*H*_3_(CH_2_CH_2_CH_2_Si)_2_), 0.00 (s, 48H, -(C*H*_3_)_2_SiCH_2_CH_2_CH_2_NH), 0.48–0.70 (m, overlapping of signals, 64H, -SiC*H*_2_CH_2_C*H*_2_Si, CH_3_Si(C*H*_2_CH_2_C*H*_2_Si)_2_ and (CH_3_)_2_SiC*H*_2_CH_2_CH_2_NH), 1.30–1.47, (m, 24H, overlapping of signals, SiCH_2_C*H*_2_CH_2_Si and CH_3_Si(CH_2_C*H*_2_CH_2_Si)_2_), 1.52–1.62 (m, 16H, (CH_3_)_2_SiCH_2_C*H*_2_CH_2_NH), 3.29 (m, 16H, (CH_3_)_2_SiCH_2_CH_2_C*H*_2_NH), 3.86 (s, 24H, OCH_3_), 6.48 (d, 8H, ^3^J_(H-H)_ = 16.2 Hz, PhCH=C*H*(CO)NH), 6.80 (d, 8H, ^3^J_(H-H)_ = 7.9 Hz, 1H_Ar,_ *ortho*-OH), 7.02 (d, 8H, ^3^J_(H-H)_ = 7.9 Hz, 1H_Ar_, *para*-OCH_3_), 7.10 (s, 8H, 1H_Ar_, *ortho*-OCH_3_), 7.47 (d, 8H, ^3^J_(H-H)_ = 16.2 Hz, PhC*H*=CH(CO)NH). ^13^C-NMR (CD_3_OD): δ (ppm) −4.1 ((*C*H_3_)_2_SiCH_2_CH_2_CH_2_NH), −2.8 (*C*H_3_Si(CH_2_CH_2_CH_2_Si)_2_), 13.7 (-(CH_3_)_2_Si*C*H_2_CH_2_CH_2_NH), 19.8, 20.0, 20.1, 21.1 (Si*C*H_2_*C*H_2_*C*H_2_Si and -CH_3_Si(*C*H_2_*C*H_2_*C*H_2_Si)_2_), 25.3 (CH_3_)_2_SiCH_2_*C*H_2_CH_2_NH), 44.0 ((CH_3_)_2_SiCH_2_CH_2_*C*H_2_NH), 56.4 (-O*C*H_3_), 111.6 (CAr, *ortho-OCH_3_*), 116.5 (CAr, *ortho-OH)*, 119.0 (CAr, PhCH=*C*H(CO)NH), 123.2, (CAr, *para-OCH_3_*), 128.3 (Cipso, *para-OH*), 142.0 (Ph*C*H=CH(CO)NH*)*, 149.2 (Cipso), 149.8 (Cipso), 169.0 (NH*C*=O). {^1^H-^15^N}-HMBC-NMR (CD_3_OD): δ (ppm) −259.6 (*N*HC=O). Elemental Analysis (%): Calc for C_160_H_260_N_8_O_24_Si_13_ (3044.98 g/mol). C, 63.11; H, 8.61; N, 3.68. Exp.: C, 64.56; H, 9.01; N, 3.96.

#### 2.1.5. Synthesis of G_2_-[Si(CH_2_)_3_NH(CO)CH=CHCH_2_Ph(OH)_2_)]_8_ (**5**)

Dendrimer **5** was prepared through the same method as described for **1** by using the following reagents: caffeic acid (300.1 mg, 1.666 mmol), EDCI·HCl (318.7 mg, 1.666 mmol), HOBt (225.1 mg, 1.666 mmol), G_2_-[Si(CH_2_)_3_NH_2_]_8_ (207.0 mg, 0.174 mmol) and NEt_3_ (1.392 mmol). Compound **5** was obtained as a brown solid (218.6 mg, 65%). ^1^H-NMR (CD_3_OD): δ (ppm) −0.04 (s, 12H, C*H*_3_(CH_2_CH_2_CH_2_Si)_2_), 0.00 (s, 48H, -(C*H*_3_)_2_SiCH_2_CH_2_CH_2_NH), 0.47–0.69 (m, overlapping of signals, 64H, -SiC*H*_2_CH_2_C*H*_2_Si, CH_3_Si(C*H*_2_CH_2_C*H*_2_Si)_2_ and (CH_3_)_2_SiC*H*_2_CH_2_CH_2_NH), 1.34–1.46, (m, 24H, overlapping of signals, SiCH_2_C*H*_2_CH_2_Si and CH_3_Si(CH_2_C*H*_2_CH_2_Si)_2_), 1.49–1.62 (m, 16H, (CH_3_)_2_SiCH_2_C*H*_2_CH_2_NH), 3.27 (m, 16H, (CH_3_)_2_SiCH_2_CH_2_C*H*_2_NH), 6.40 (d, 8H, ^3^J_(H-H)_ = 15.7 Hz, PhCH=C*H*(CO)NH), 6.77 (d, 8H, ^3^J_(H-H)_ = 8.1 Hz, 1H_Ar_, *meta*-CH=CH), 6.91 (d, 8H, ^3^J_(H-H)_ = 8.1 Hz, 1H_Ar_, *ortho*-CH=CH), 7.03 (s, 8H, 1H_Ar,_ *ortho-*CH=CH*, ortho-*OH), 7.41 (d, 8H, ^3^J_(H-H)_ = 15.7 Hz, PhC*H*=CH(CO)NH). ^13^C-NMR (CD_3_OD): δ (ppm) −4.2 ((*C*H_3_)_2_SiCH_2_CH_2_CH_2_NH), −2.9 (*C*H_3_Si(CH_2_CH_2_CH_2_Si)_2_), 13.7 (-(CH_3_)_2_Si*C*H_2_CH_2_CH_2_NH), 19.8, 20.0, 20.1, 21.1 (Si*C*H_2_*C*H_2_*C*H_2_Si and -CH_3_Si(*C*H_2_*C*H_2_*C*H_2_Si)_2_), 25.2 (CH_3_)_2_SiCH_2_*C*H_2_CH_2_NH), 44.0 ((CH_3_)_2_SiCH_2_CH_2_*C*H_2_NH), 115.2 (CAr, *ortho-OH, ortho-CH=CH*), 116.5 (CAr, *meta-CH=CH*), 118.6 (PhCH=*C*H(CO)NH), 122.1, (CAr, *ortho-CH=CH*), 128.4 (Cipso, *meta-OH, para-OH*), 142.1 (Ph*C*H=CH(CO)NH*)*, 146.7 (Cipso), 148.7 (Cipso), 169.2 (NH*C*=O). {^1^H-^15^N}-HMBC-NMR (CD_3_OD): δ (ppm) −259.6 (*N*HC=O). Elemental Analysis (%): Calc for C_152_H_244_N_8_O_24_Si_13_ (2932.76 g/mol). C, 62.25; H, 8.39; N, 3.82. Exp.: C, 60.60; H, 8.49; N, 4.89.

#### 2.1.6. Synthesis of G_2_-[Si(CH_2_)_3_NH(CO)Ph(OH)_3_]_8_ (**6**)

Dendrimer **6** wasprepared through the same method as described for **1** by using the following reagents: gallic acid (385.4 mg, 2.049 mmol), EDCI·HCl (391.9 mg, 2.049 mmol), HOBt (272.7 mg, 2.049 mmol), G_2_-[Si(CH_2_)_3_NH_2_]_8_ (352.0 mg, 0.213 mmol) and NEt_3_ (1.704 mmol). Compound **6** was obtained as a brown solid (455.7 mg, 75%). ^1^H-NMR (CD_3_OD): δ (ppm) −0.07 (s, 12H, C*H*_3_(CH_2_CH_2_CH_2_Si)_2_), −0.03 (s, 48H, -(C*H*_3_)_2_SiCH_2_CH_2_CH_2_NH), 0.45–0.68 (m, overlapping of signals, 64H, -SiC*H*_2_CH_2_C*H*_2_Si, CH_3_Si(C*H*_2_CH_2_C*H*_2_Si)_2_ and (CH_3_)_2_SiC*H*_2_CH_2_CH_2_NH), 1.31–1.44, (m, 24H, overlapping of signals, SiCH_2_C*H*_2_CH_2_Si and CH_3_Si(CH_2_C*H*_2_CH_2_Si)_2_), 1.51–1.65 (m, 16H, (CH_3_)_2_SiCH_2_C*H*_2_CH_2_NH), 3.27 (m, 16H, (CH_3_)_2_SiCH_2_CH_2_C*H*_2_NH), 6.85 (s, 16H), 7.03 (broad s, overlapping of signals, 32H, NH and Ph(OH)). ^13^C-NMR (CD_3_OD): δ (ppm). −4.3 ((*C*H_3_)_2_SiCH_2_CH_2_CH_2_NH), −2.9 (*C*H_3_Si(CH_2_CH_2_CH_2_Si)_2_), 13.7 ((CH_3_)_2_Si*C*H_2_CH_2_CH_2_NH), 19.8, 19.9, 20.1, 20.2 (Si*C*H_2_*C*H_2_*C*H_2_Si and -CH_3_Si(*C*H_2_*C*H_2_*C*H_2_Si)_2_), 25.3 ((CH_3_)_2_SiCH_2_*C*H_2_CH_2_NH), 44.3 ((CH_3_)_2_SiCH_2_CH_2_*C*H_2_NH), 107.8 (CAr, *ortho-OH*), 126.4 (Cipso, *para-OH*), 146.6 (Cipso, *ortho-OH*), 170.4 (NH*C*=O). {^1^H-^15^N}-HMBC-NMR (CD_3_OD): δ (ppm) −266.6 (NHC=O). Elemental Analysis (%): Calc for C_136_H_228_N_8_O_32_Si_13_ (2852.45 g/mol). C, 57.27; H, 8.06; N, 3.93. Exp.: C, 56.77; H, 8.98; N, 4.79.

The article will be updated and the original [1] will remain on the webpage.

## Data Availability

The data presented in this study are available in Supplementary Materials.

## Supplementary Materials

The following are available online at https://www.mdpi.com/1999-4923/13/1/121/s1, Figure S1. Mass Spectrometry (ESI-TOF) of dendritic polyphenol (**1**). Figure S2. 1H-NMR (500 MHz, CD3OD) of dendritic polyphenol (**1**). Figure S3. 13C-NMR (500 MHz, CD3OD) of dendritic polyphenol (**1**). Figure S4. {1H-15N}-HMBC-NMR (500 MHz, CD3OD) of dendritic polyphenol (**1**). Figure S5. 1H-DOSY-2D-NMR (500 MHz, CD3OD) of dendritic polyphenol (**1**). Figure S6. {1H-1H}-COSY-2D-NMR (500 MHz, CD3OD) of dendritic polyphenol (**1**). Figure S7. {1H-13C}-HSQC-2D-NMR (500 MHz, CD3OD) of dendritic polyphenol (**1**). Figure S8. {1H-13C}-HMBC-2D-NMR (500 MHz, CD3OD) of dendritic polyphenol (**1**). Figure S9. Mass Spectrometry (ESI-TOF) of dendritic polyphenol (**2**). Figure S10. 1H-NMR (500 MHz, CD3OD) of dendritic polyphenol (**2**). Figure S11. 13C-NMR (500 MHz, CD3OD) of dendritic polyphenol (**2**). Figure S12. {1H-15N}-HMBC-NMR (500 MHz, CD3OD) of dendritic polyphenol (**2**). Figure S13. 1H-DOSY-2D-NMR (500 MHz, CD3OD) of dendritic polyphenol (**2**). Figure S14. {1H-1H}-COSY-2D-NMR (500 MHz, CD3OD) of dendritic polyphenol (**2**). Figure S15. {1H-13C}-HSQC-2D-NMR (500 MHz, CD3OD) of dendritic polyphenol (**2**). Figure S16. Mass Spectrometry (ESI-TOF) of dendritic polyphenol (**3**). Figure S17. 1H-NMR (500 MHz, CD3OD) of dendritic polyphenol (**3**). Figure S18. 13C-NMR (500 MHz, CD3OD) of dendritic polyphenol (**3**). Figure S19. {1H-15N}-HMBC-NMR (500 MHz, CD3OD) of dendritic polyphenol (**3**). Figure S20. 1H-DOSY-2D-NMR (500 MHz, CD3OD) of dendritic polyphenol (**3**). Figure S21. {1H-1H}-COSY-2D-NMR (500 MHz, CD3OD) of dendritic polyphenol (**3**). Figure S22. {1H-13C}-HSQC-2D-NMR (500 MHz, CD3OD) of dendritic polyphenol (**3**). Figure S23. 1H-NMR (500 MHz, CD3OD) of dendritic polyphenol (**4**). Figure S24. 13C-NMR (500 MHz, CD3OD) of dendritic polyphenol (**4**). Figure S25. {1H-15N}-HMBC-NMR (500 MHz, CD3OD) of dendritic polyphenol (**4**). Figure S26. 1H-DOSY-2D-NMR (500 MHz, CD3OD) of dendritic polyphenol (**4**). Figure S27. {1H-1H}-COSY-2D-NMR (500 MHz, CD3OD) of dendritic polyphenol (**4**). Figure S28. {1H-13C}-HSQC-2D-NMR (500 MHz, CD3OD) of dendritic polyphenol (**4**). Figure S29. {1H-13C}-HMBC-2D-NMR (500 MHz, CD3OD) of dendritic polyphenol (**4**). Figure S30. 1H-NMR (500 MHz, CD3OD) of dendritic polyphenol (**5**). Figure S31. 13C-NMR (500 MHz, CD3OD) of dendritic polyphenol (**5**). Figure S32. {1H-15N}-HMBC-NMR (500 MHz, CD3OD) of dendritic polyphenol (**5**). Figure S33. 1H-DOSY-2D-NMR (500 MHz, CD3OD) of dendritic polyphenol (**5**). Figure S34. {1H-13C}-HSQC-2D-NMR (500 MHz, CD3OD) of dendritic polyphenol (**5**). Figure S35. {1H-13C}-HMBC-2D-NMR (500 MHz, CD3OD) of dendritic polyphenol (**5**). Figure S36. 1H-NMR (500 MHz, CD3OD) of dendritic polyphenol (**6**). Figure S37. 13C-NMR (500 MHz, CD3OD) of dendritic polyphenol (**6**). Figure S38. {1H-15N}-HMBC-NMR (500 MHz, CD3OD) of dendritic polyphenol (**6**). Figure S39. 1H-DOSY-2D-NMR (500 MHz, CD3OD) of dendritic polyphenol (**6**). Figure S40. {1H-1H}-COSY-2D-NMR (500 MHz, CD3OD) of dendritic polyphenol (**6**). Figure S41. {1H-13C}-HSQC-2D-NMR (500 MHz, CD3OD) of dendritic polyphenol (**6**). Figure S42. (A) A representative calibration curve of inhibition of DPPH by Trolox standards. Representative results of at least three independent experiments are shown. (B) Graphics with equations line for compound G1-[Si(CH2)3NH(CO)Ph(OH)3]4 (**3**).
