# Supplementary material for: Antioxidant and Antibacterial Properties of Carbosilane Dendrimers Functionalized with Polyphenolic Moieties
Source: Pharmaceutics. 2020 Jul 24;12(8):698. doi: 10.3390/pharmaceutics12080698 (PMC7464503; doi:10.3390/pharmaceutics12080698)
Supplement: Supplementary file 1 [file pharmaceutics-12-00698-s001.pdf]

pharmaceutics

Supplementary Materials: Antioxidant and Antibacterial Properties of Carbosilane Dendrimers Functionalised with Polyphenolic Moieties

Natalia Sanz del Olmo, Cornelia E. Peña González, Jose Daniel Rojas, Rafael Gómez, Paula Ortega, Alberto Escarpa and Francisco Javier de la Mata

Table of contents

Figure S1. Mass Spectrometry (ESI-TOF) of dendritic polyphenol **(1)**

Figure S2. ^{1}H-NMR (500 MHz, CD_3OD) of dendritic polyphenol **(1)**

Figure S3. ^{13}C-NMR (500 MHz, CD_3OD) of dendritic polyphenol **(1)**

Figure S4. {^{1}H-^{15}N}-HMBC-NMR (500 MHz, CD_3OD) of dendritic polyphenol **(1)**

Figure S5. ^{1}H-DOSY-2D-NMR (500 MHz, CD_3OD) of dendritic polyphenol **(1)**

Figure S6. {^{1}H-^{1}H}-COSY-2D-NMR (500 MHz, CD_3OD) of dendritic polyphenol **(1)**

Figure S7. {^{1}H-^{13}C}-HSQC-2D-NMR (500 MHz, CD_3OD) of dendritic polyphenol **(1)**

Figure S8. {^{1}H-^{13}C}-HMBC-2D-NMR (500 MHz, CD_3OD) of dendritic polyphenol **(1)**

Figure S9. Mass Spectrometry (ESI-TOF) of dendritic polyphenol **(2)**

Figure S10. ^{1}H-NMR (500 MHz, CD_3OD) of dendritic polyphenol **(2)**

Figure S11. ^{13}C-NMR (500 MHz, CD_3OD) of dendritic polyphenol **(2)**

Figure S12. {^{1}H-^{15}N}-HMBC-NMR (500 MHz, CD_3OD) of dendritic polyphenol **(2)**

Figure S13. ^{1}H-DOSY-2D-NMR (500 MHz, CD_3OD) of dendritic polyphenol **(2)**

Figure S14. {^{1}H-^{1}H}-COSY-2D-NMR (500 MHz, CD_3OD) of dendritic polyphenol **(2)**

Figure S15. {^{1}H-^{13}C}-HSQC-2D-NMR (500 MHz, CD_3OD) of dendritic polyphenol **(2)**

Figure S16. Mass Spectrometry (ESI-TOF) of dendritic polyphenol **(3)**

Figure S17. ^{1}H-NMR (500 MHz, CD_3OD) of dendritic polyphenol **(3)**

Figure S18. ^{13}C-NMR (500 MHz, CD_3OD) of dendritic polyphenol **(3)**

Figure S19. {^{1}H-^{15}N}-HMBC-NMR (500 MHz, CD_3OD) of dendritic polyphenol **(3)**

Figure S20. ^{1}H-DOSY-2D-NMR (500 MHz, CD_3OD) of dendritic polyphenol **(3)**

Figure S21. {^{1}H-^{1}H}-COSY-2D-NMR (500 MHz, CD_3OD) of dendritic polyphenol **(3)**

Figure S22. {^{1}H-^{13}C}-HSQC-2D-NMR (500 MHz, CD_3OD) of dendritic polyphenol **(3)**

Figure S23. ^{1}H-NMR (500 MHz, CD_3OD) of dendritic polyphenol **(4)**

Figure S24. ^{13}C-NMR (500 MHz, CD_3OD) of dendritic polyphenol **(4)**

Figure S25. {^{1}H-^{15}N}-HMBC-NMR (500 MHz, CD_3OD) of dendritic polyphenol **(4)**

Figure S26. ^{1}H-DOSY-2D-NMR (500 MHz, CD_3OD) of dendritic polyphenol **(4)**

Figure S27. {^{1}H-^{1}H}-COSY-2D-NMR (500 MHz, CD_3OD) of dendritic polyphenol **(4)**

Figure S28. {^{1}H-^{13}C}-HSQC-2D-NMR (500 MHz, CD_3OD) of dendritic polyphenol **(4)**

Figure S29. {^{1}H-^{13}C}-HMBC-2D-NMR (500 MHz, CD_3OD) of dendritic polyphenol **(4)**

Figure S30. ^{1}H-NMR (500 MHz, CD_3OD) of dendritic polyphenol **(5)**

Figure S31. ^{13}C-NMR (500 MHz, CD_3OD) of dendritic polyphenol **(5)**

Figure S32. {^{1}H-^{15}N}-HMBC-NMR (500 MHz, CD_3OD) of dendritic polyphenol **(5)**

Figure S33. ^{1}H-DOSY-2D-NMR (500 MHz, CD_3OD) of dendritic polyphenol **(5)**

Figure S34. {^{1}H-^{13}C}-HSQC-2D-NMR (500 MHz, CD_3OD) of dendritic polyphenol **(5)**

Figure S35. {^{1}H-^{13}C}-HMBC-2D-NMR (500 MHz, CD_3OD) of dendritic polyphenol **(5)**

Figure S36. ^{1}H-NMR (500 MHz, CD_3OD) of dendritic polyphenol **(6)**

Figure S37. ^{13}C-NMR (500 MHz, CD_3OD) of dendritic polyphenol **(6)**

Figure S38. {^{1}H-^{15}N}-HMBC-NMR (500 MHz, CD_3OD) of dendritic polyphenol **(6)**

Figure S39. ^{1}H-DOSY-2D-NMR (500 MHz, CD_3OD) of dendritic polyphenol **(6)**

Figure S40. {^{1}H-^{1}H}-COSY-2D-NMR (500 MHz, CD_3OD) of dendritic polyphenol **(6)**

Figure S41. {^{1}H-^{13}C}-HSQC-2D-NMR (500 MHz, CD_3OD) of dendritic polyphenol **(6)**

Figure S42. A) A representative calibration curve of inhibition of DPPH by Trolox standards. Representative results of at least three independent experiments are shown. B) Graphics with equations line for compound G_1-$[Si(CH_2)_3NH(CO)Ph(OH)_3]_4$ **(3)**.

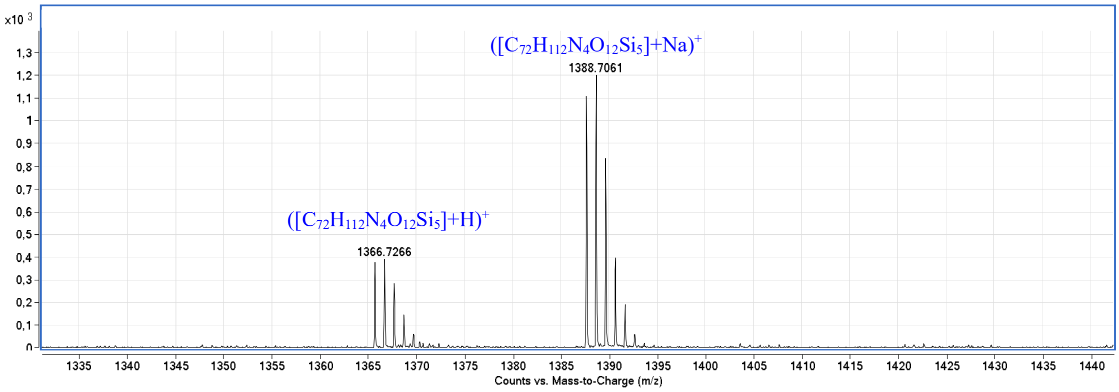

Figure S1. Mass Spectrometry (ESI-TOF) of dendritic polyphenol **(1)**

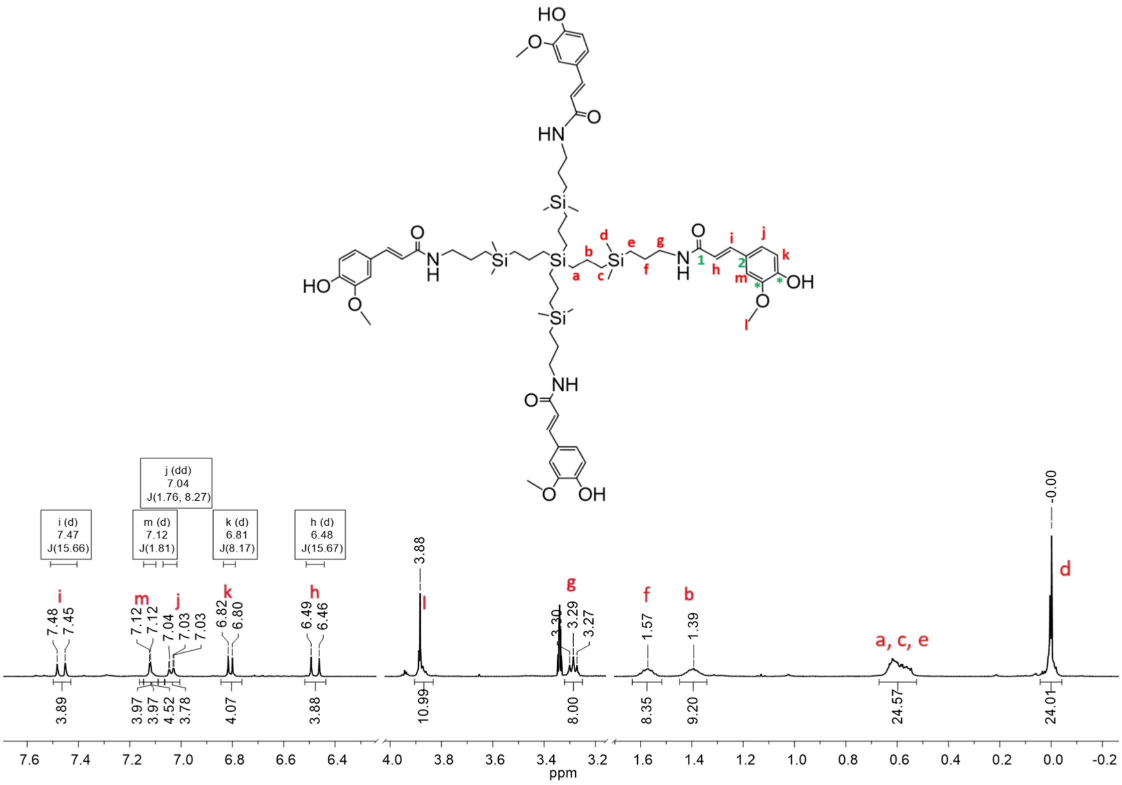

Figure S2. ^{1}H-NMR (500 MHz, CD_3OD) of dendritic polyphenol **(1)**

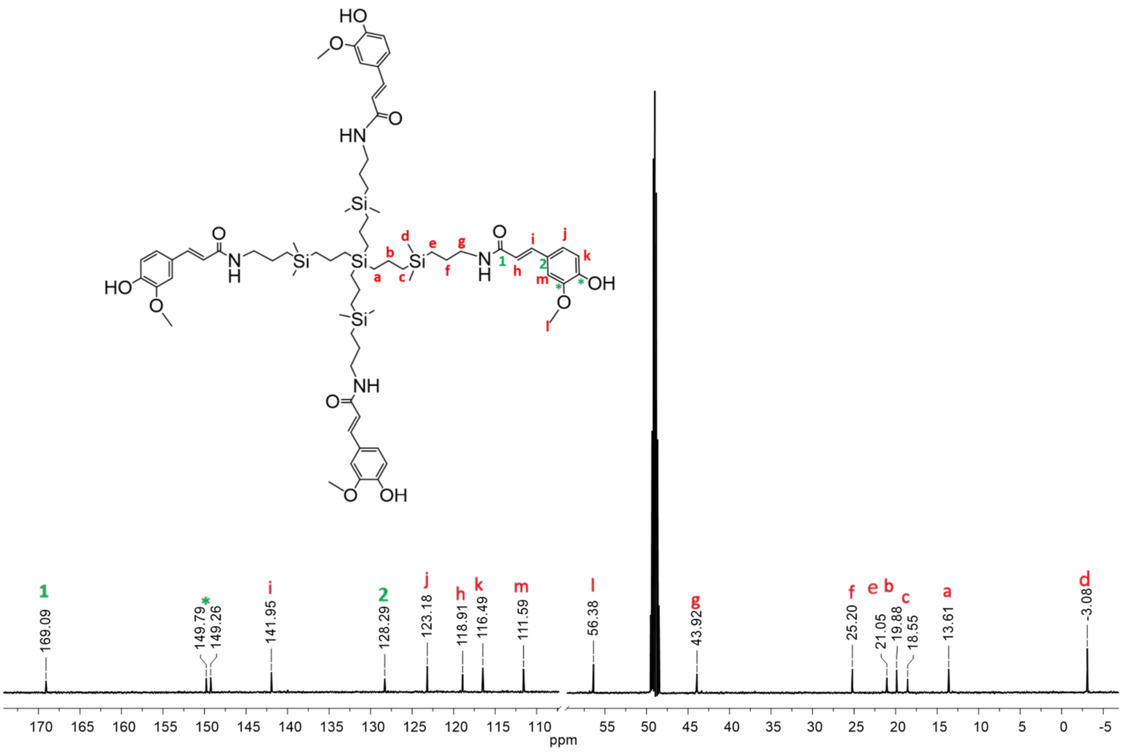

Figure S3. ^{13}C-NMR (500 MHz, CD_3OD) of dendritic polyphenol **(1)**

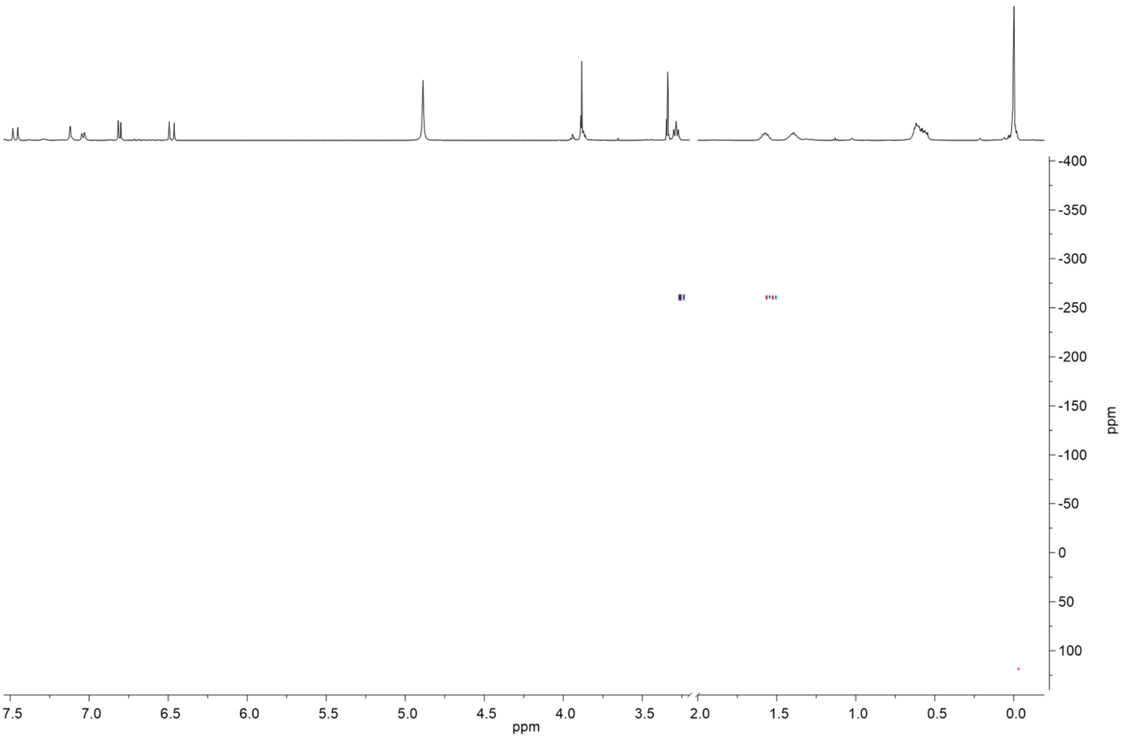

Figure S4. {^{1}H-^{15}N}-HMBC-NMR (500 MHz, CD_3OD) of dendritic polyphenol **(1)**

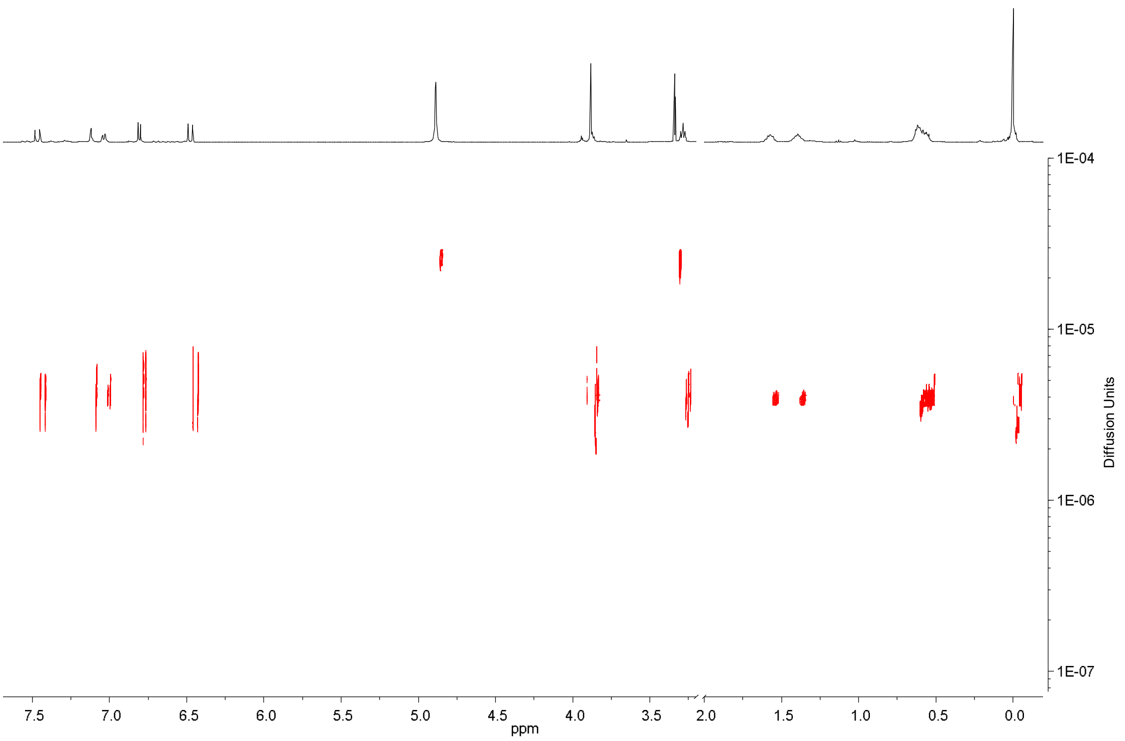

Figure S5. ^{1}H-DOSY-2D-NMR (500 MHz, CD_3OD) of dendritic polyphenol **(1)**

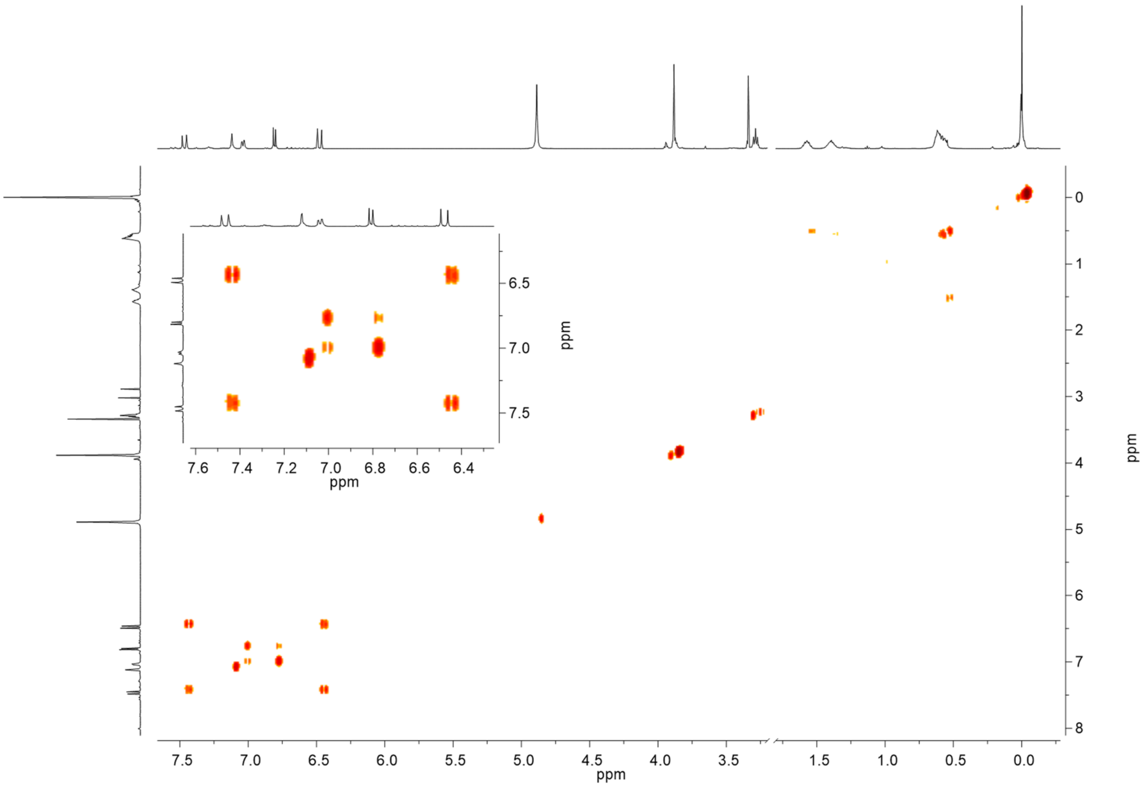

Figure S6. {^{1}H-^{1}H}-COSY-2D-NMR (500 MHz, CD_3OD) of dendritic polyphenol **(1)**

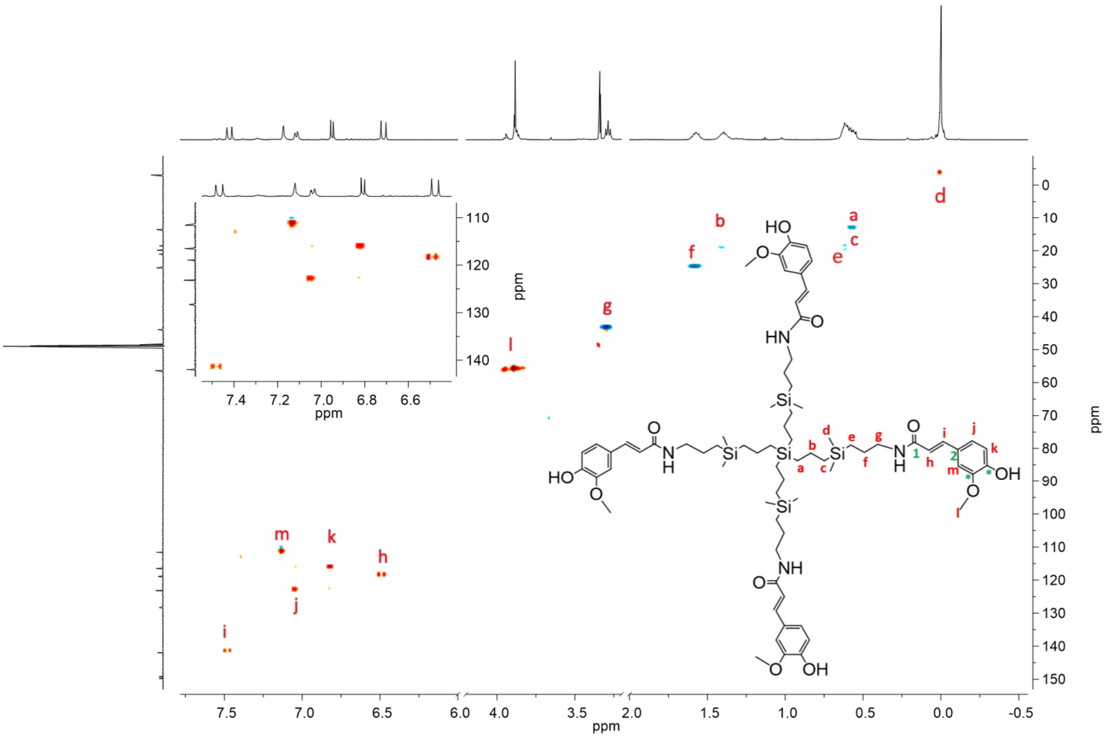

Figure S7. {^{1}H-^{13}C}-HSQC-2D-NMR (500 MHz, CD_3OD) of dendritic polyphenol **(1)**

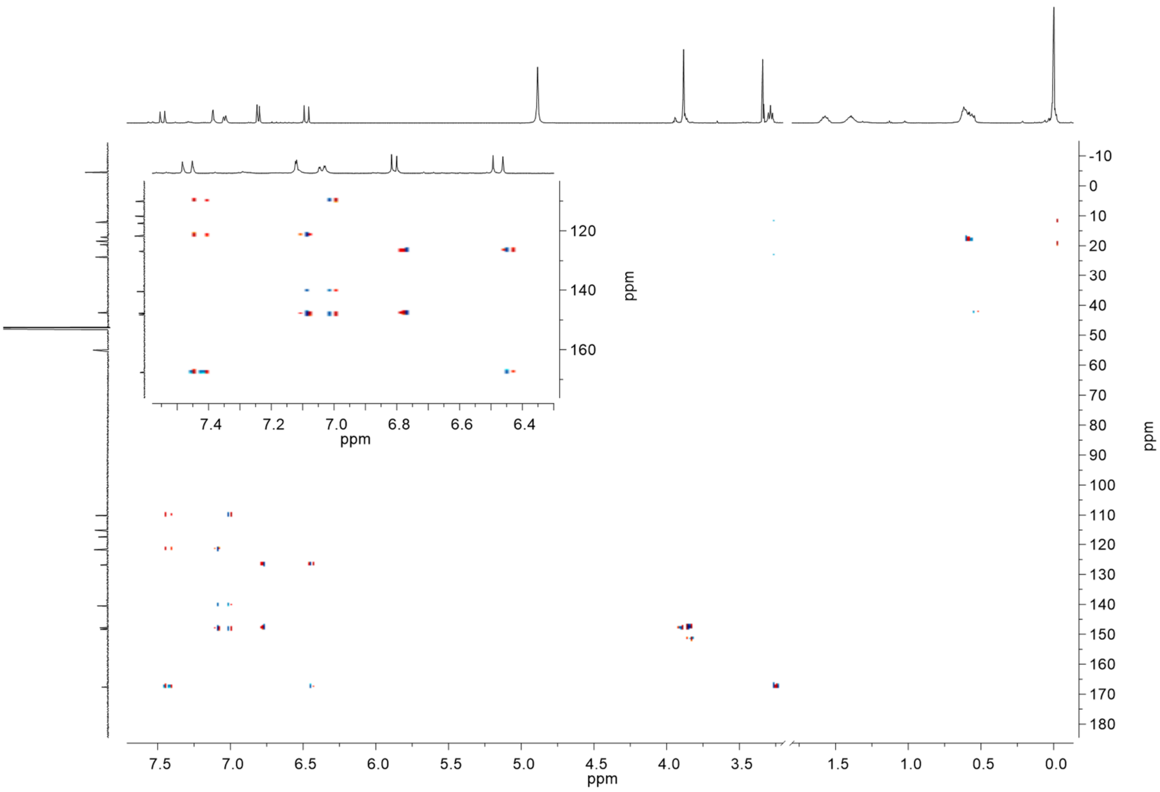

Figure S8. {^{1}H-^{13}C}-HMBC-2D-NMR (500 MHz, CD_3OD) of dendritic polyphenol **(1)**

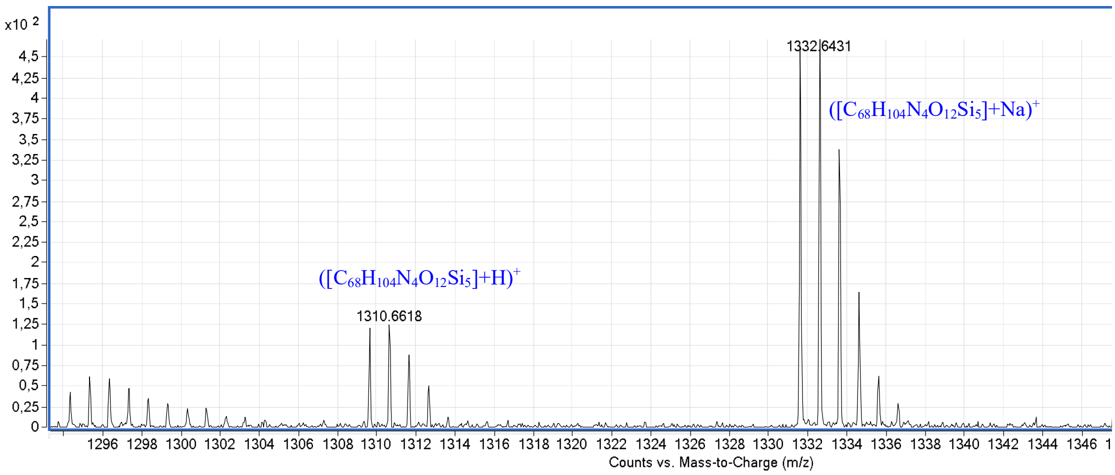

Figure S9. Mass Spectrometry (ESI-TOF) of dendritic polyphenol **(2)**

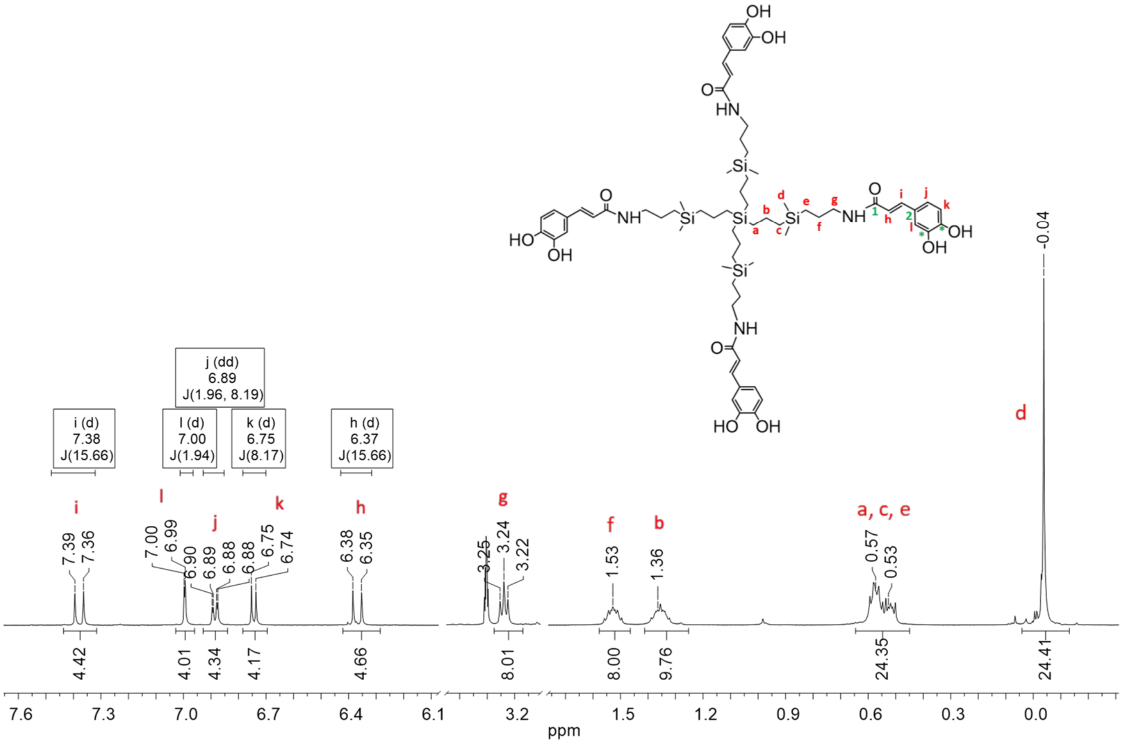

Figure S10. ^{1}H-NMR (500 MHz, CD_3OD) of dendritic polyphenol **(2)**

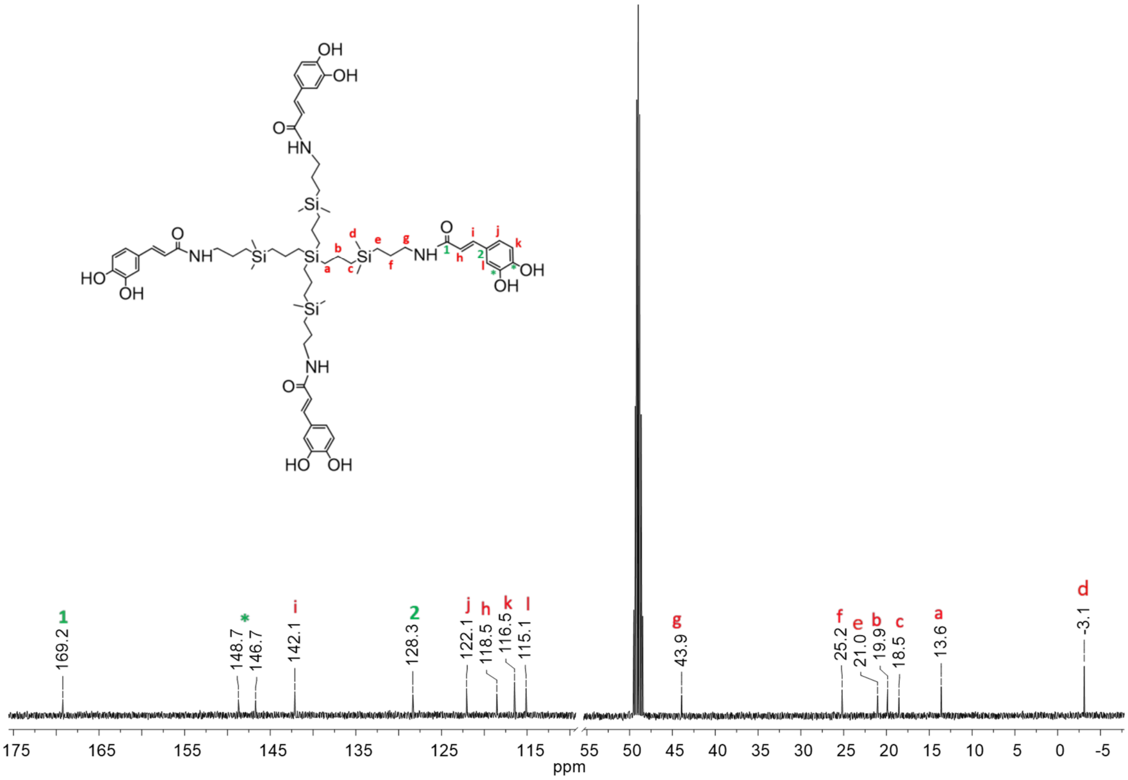

Figure S11. ^{13}C-NMR (500 MHz, CD_3OD) of dendritic polyphenol **(2)**

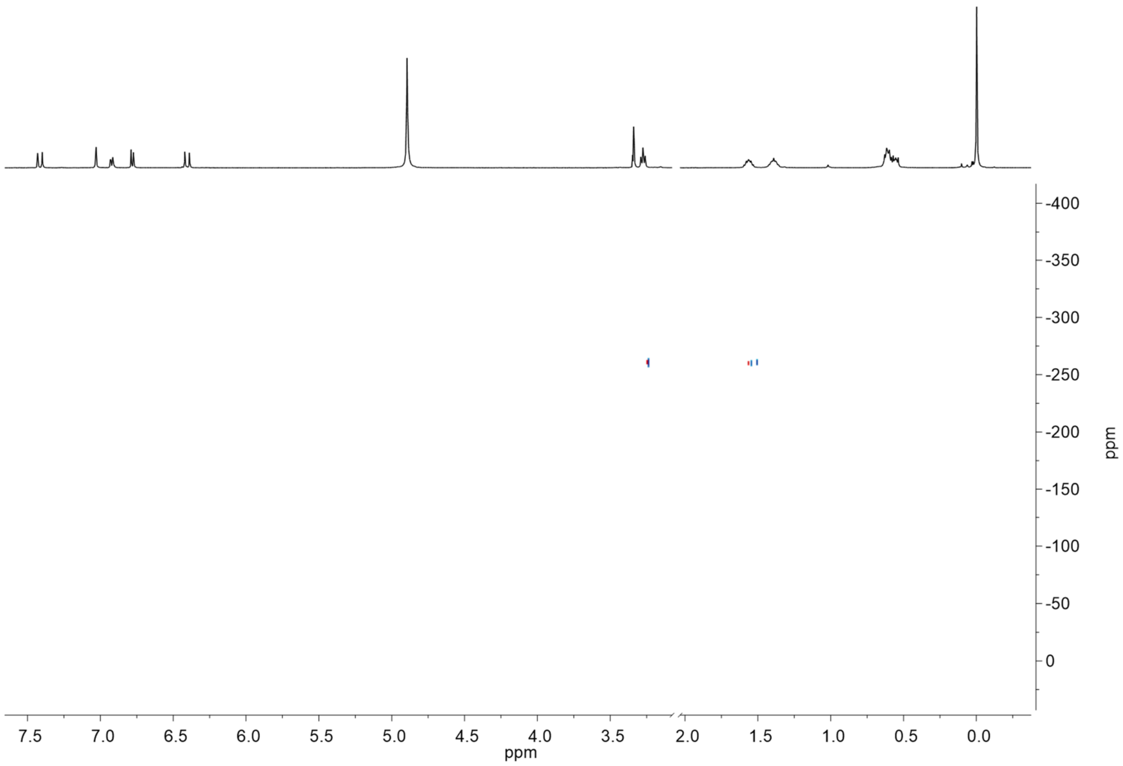

Figure S12. {^{1}H-^{15}N}-HMBC-NMR (500 MHz, CD_3OD) of dendritic polyphenol **(2)**

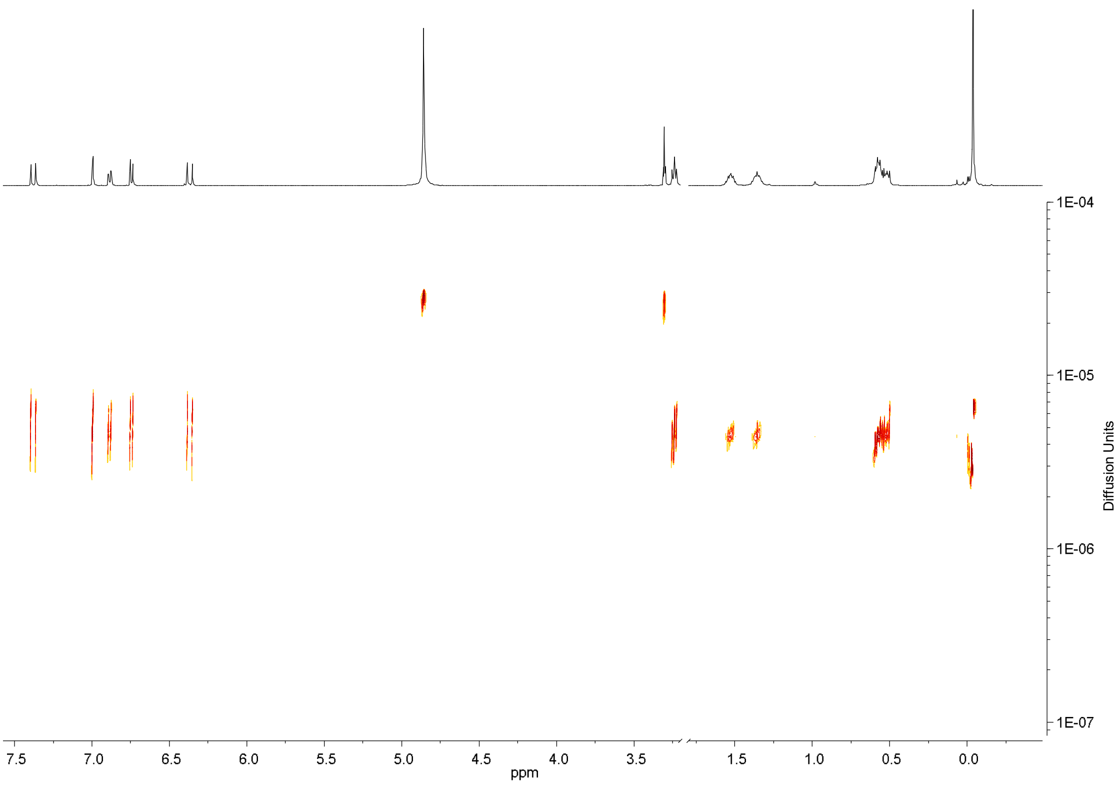

Figure S13. ^{1}H-DOSY-2D-NMR (500 MHz, CD_3OD) of dendritic polyphenol **(2)**

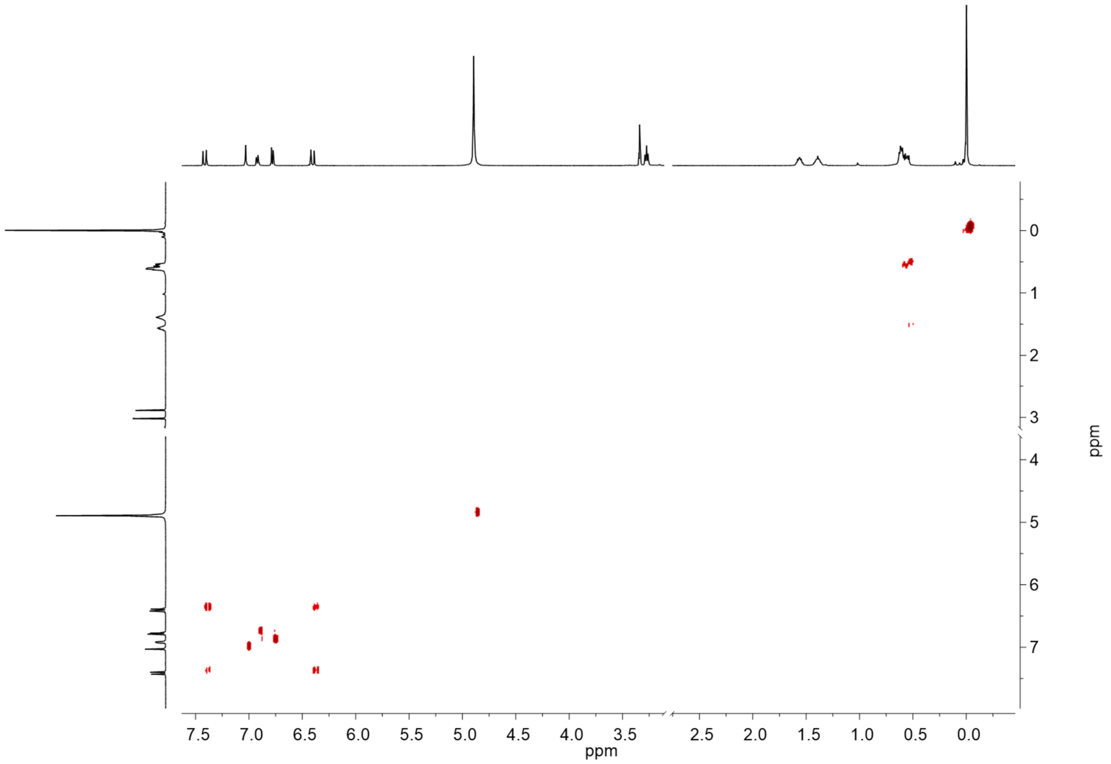

Figure S14. {^{1}H-^{1}H}-COSY-2D-NMR (500 MHz, CD_3OD) of dendritic polyphenol **(2)**

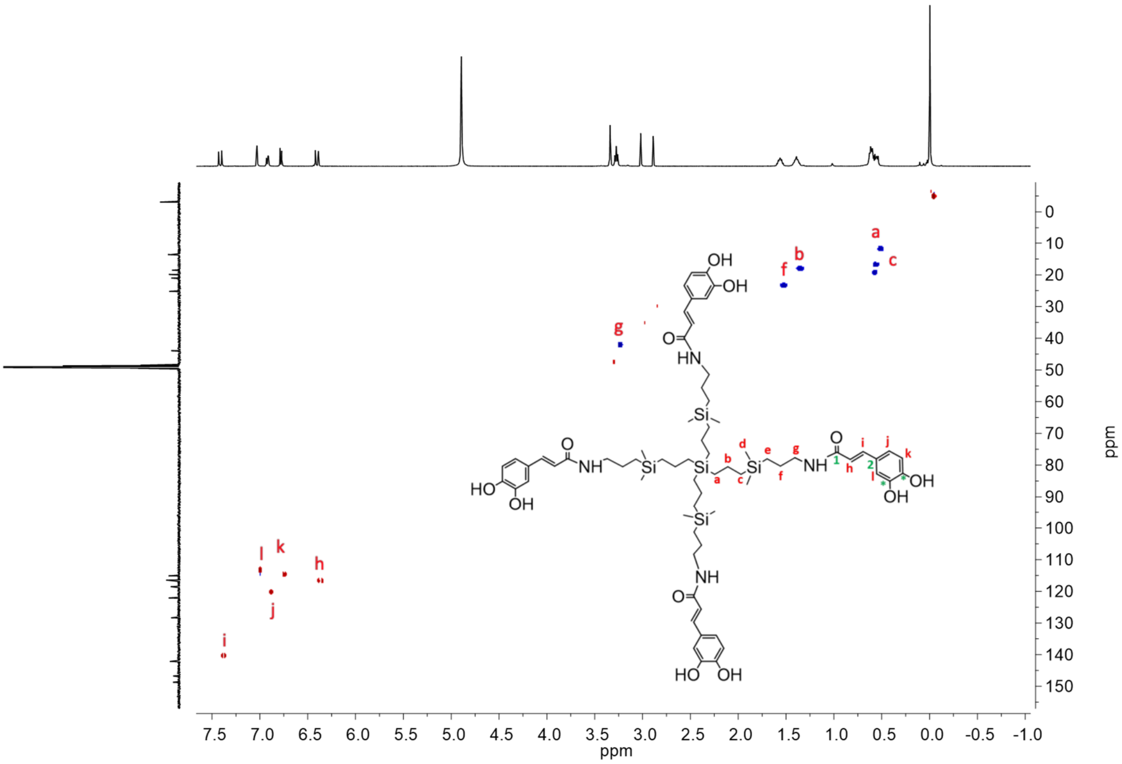

Figure S15. {^{1}H-^{13}C}-HSQC-2D-NMR (500 MHz, CD_3OD) of dendritic polyphenol **(2).**

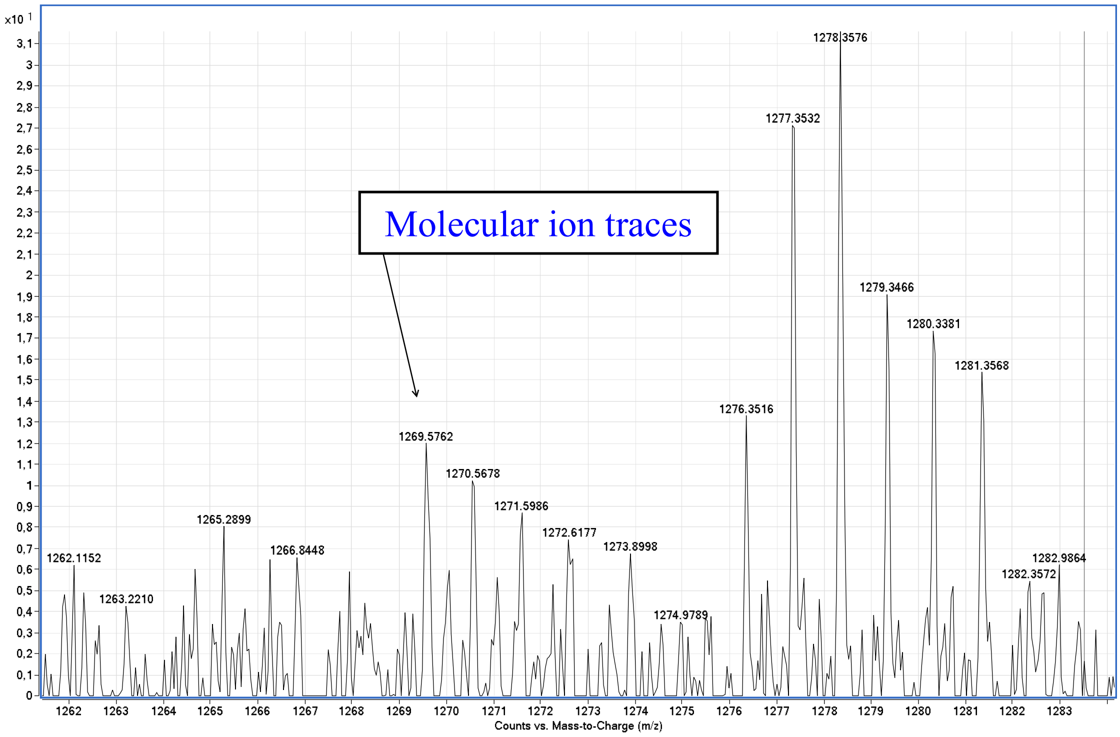

Figure S16. Mass Spectrometry (ESI-TOF) of dendritic polyphenol **(3)**

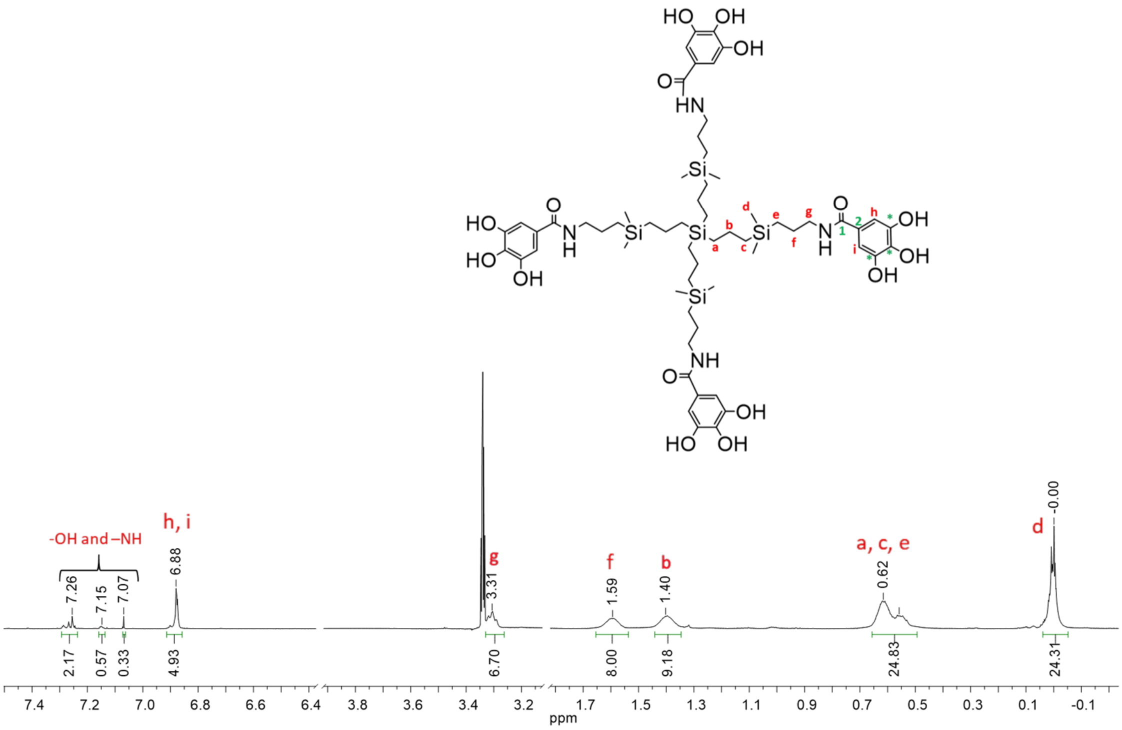

Figure S17. ^{1}H-NMR (500 MHz, CD_3OD) of dendritic polyphenol **(3)**

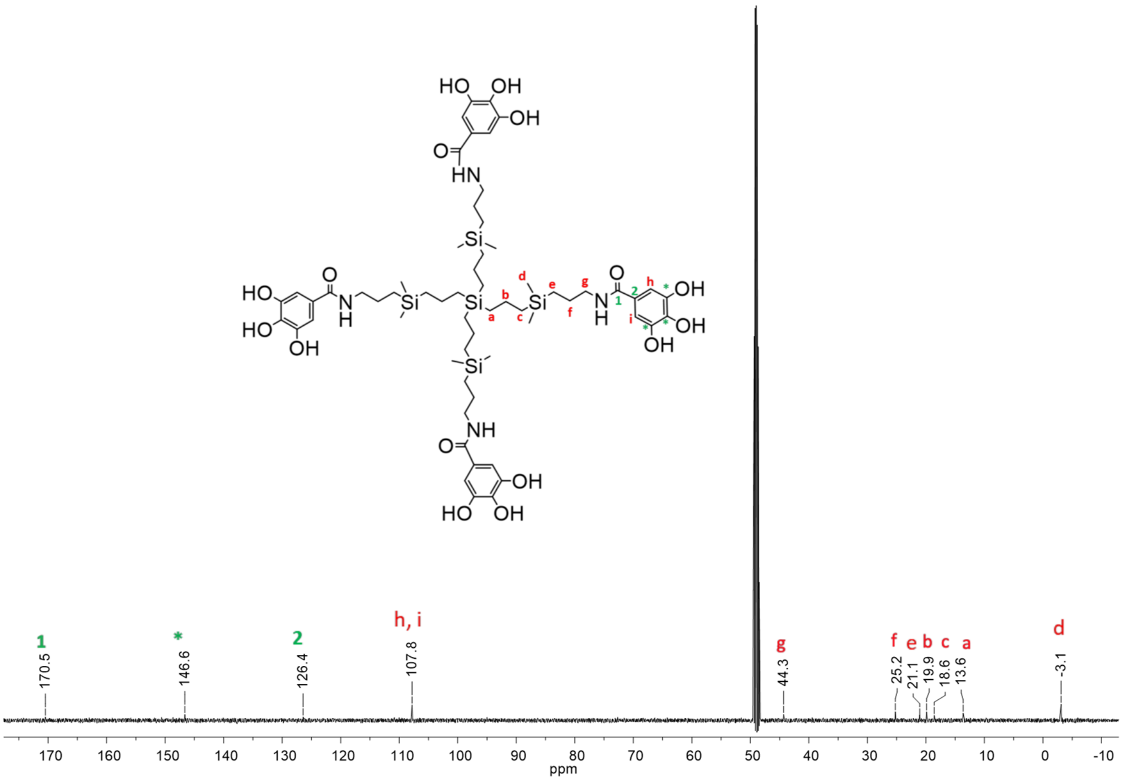

Figure S18. ^{13}C-NMR (500 MHz, CD_3OD) of dendritic polyphenol **(3)**

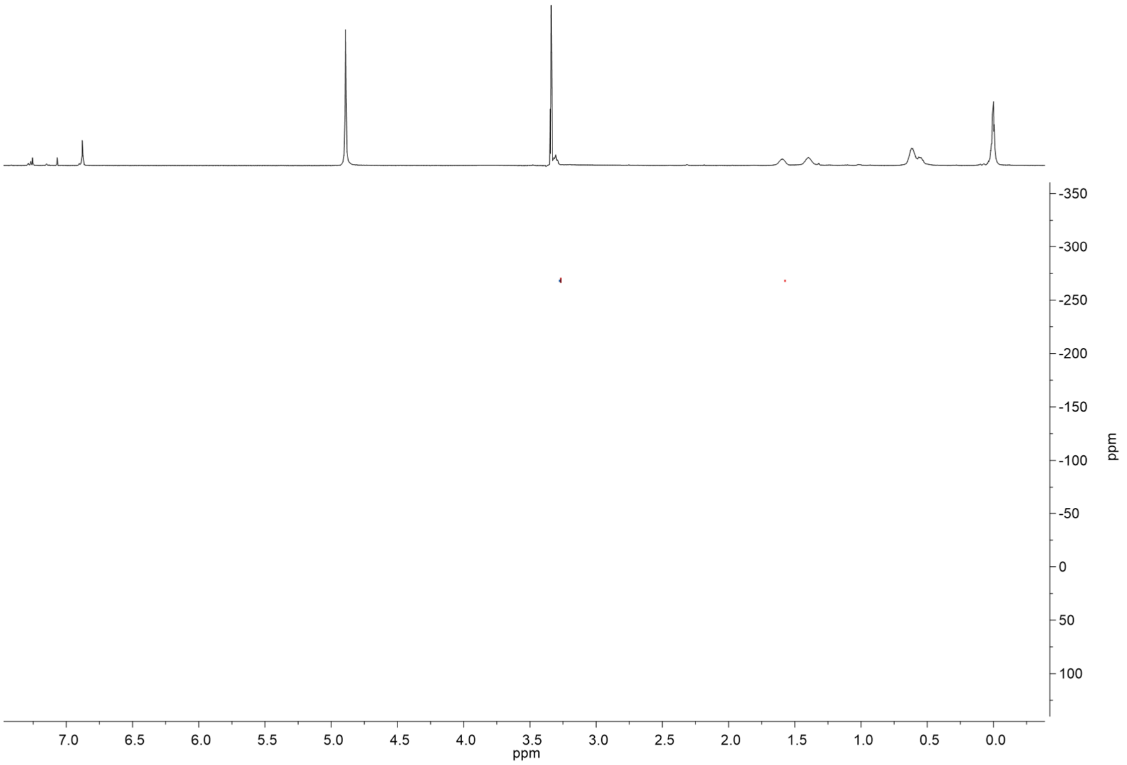

Figure S19. {^{1}H-^{15}N}-HMBC-NMR (500 MHz, CD_3OD) of dendritic polyphenol **(3)**

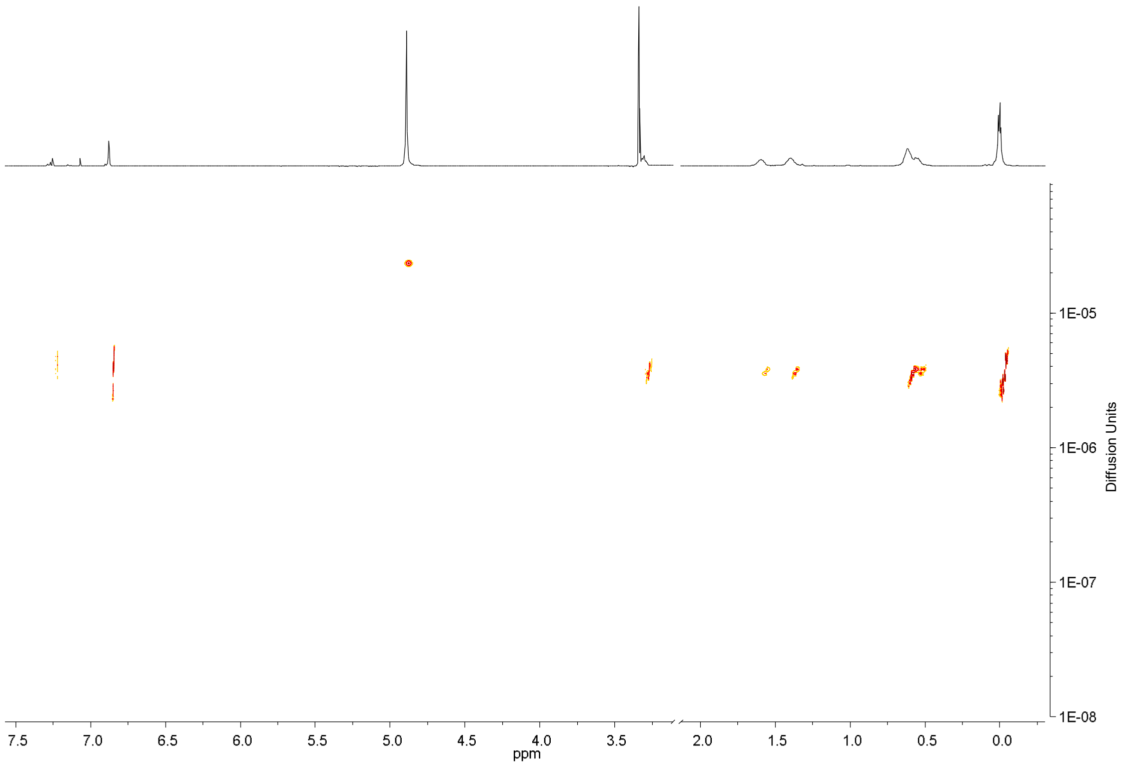

Figure S20. ^{1}H-DOSY-2D-NMR (500 MHz, CD_3OD) of dendritic polyphenol **(3)**

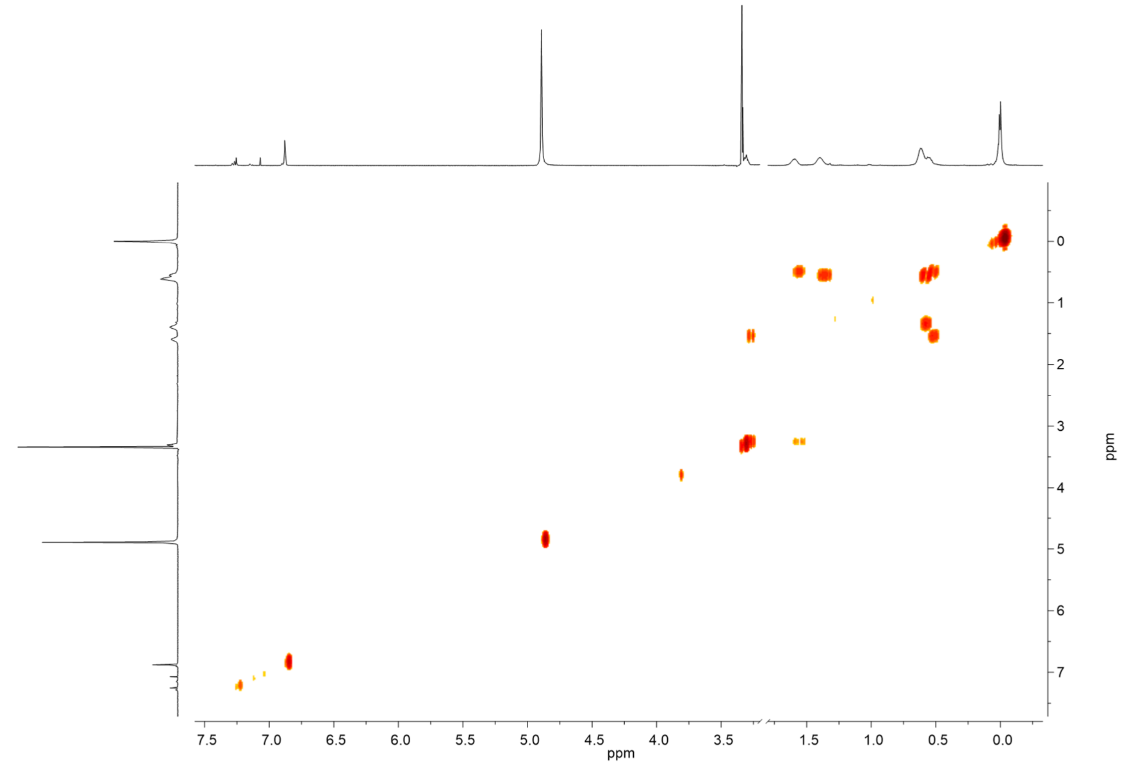

Figure S21. {¹H-¹H}-COSY-2D-NMR (500 MHz, CD_3OD) of dendritic polyphenol **(3)**

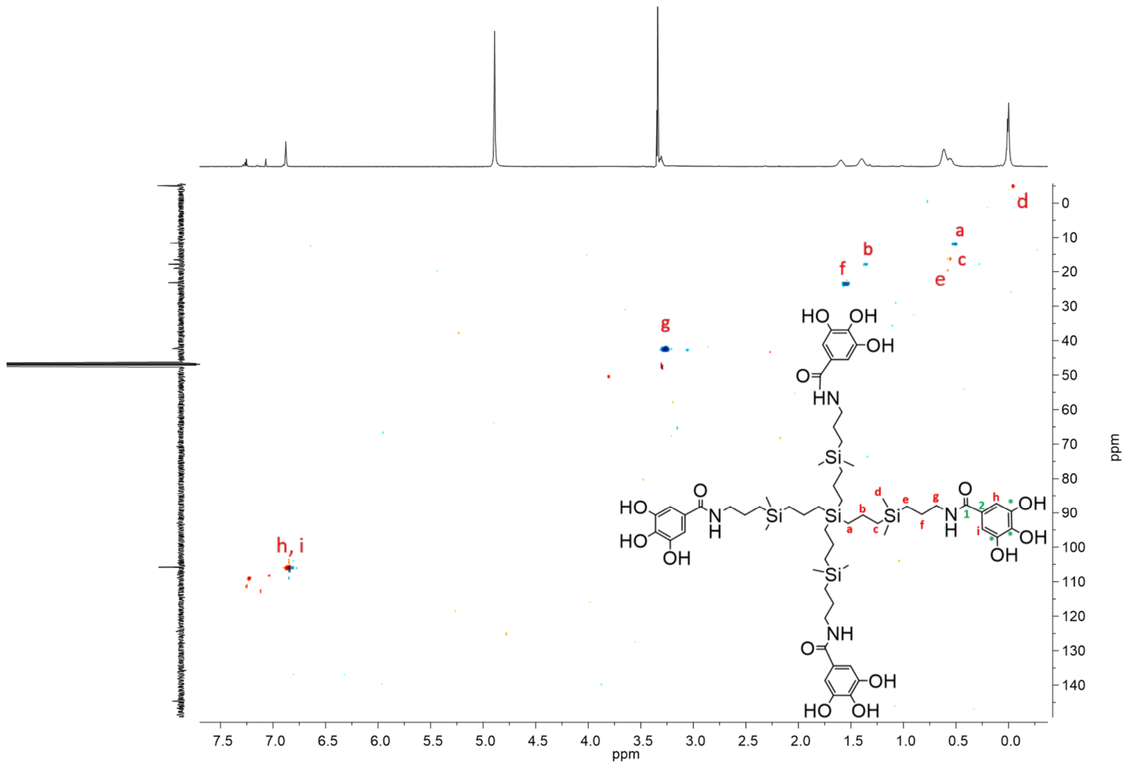

Figure S22. {¹H-¹³C}-HSQC-2D-NMR (500 MHz, CD_3OD) of dendritic polyphenol **(3)**

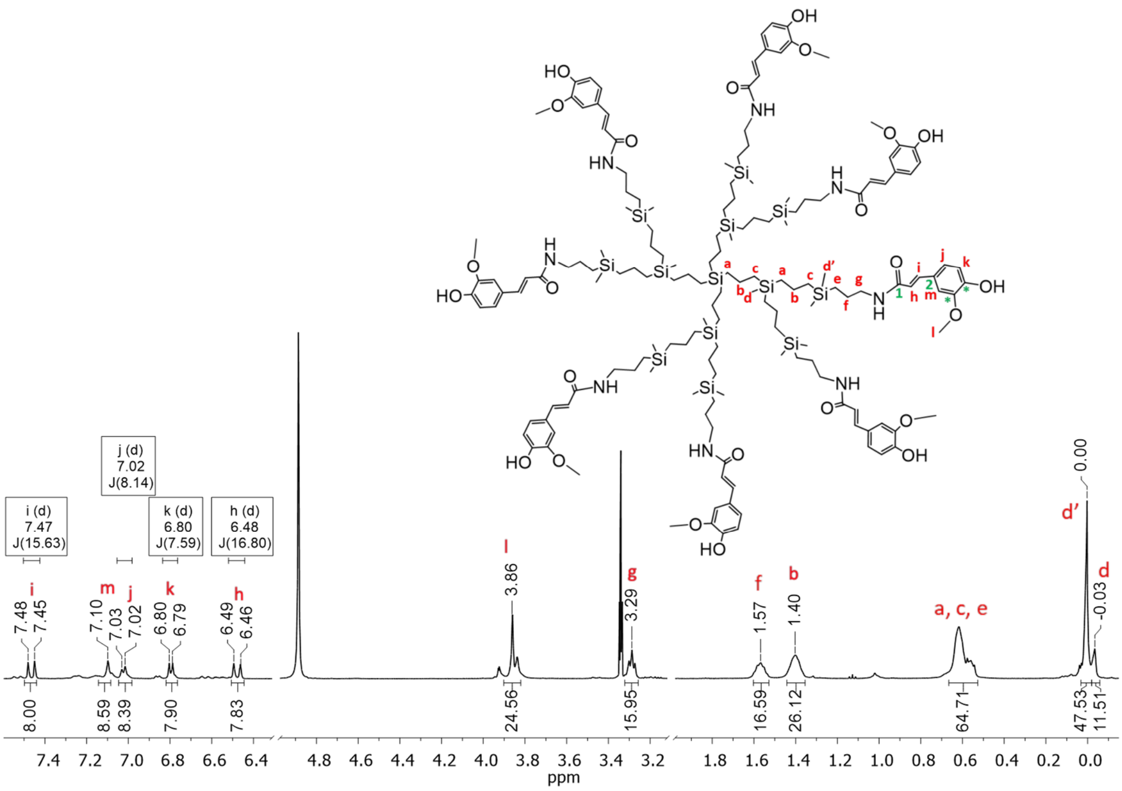

Figure S23. ^{1}H-NMR (500 MHz, CD_3OD) of dendritic polyphenol **(4)**

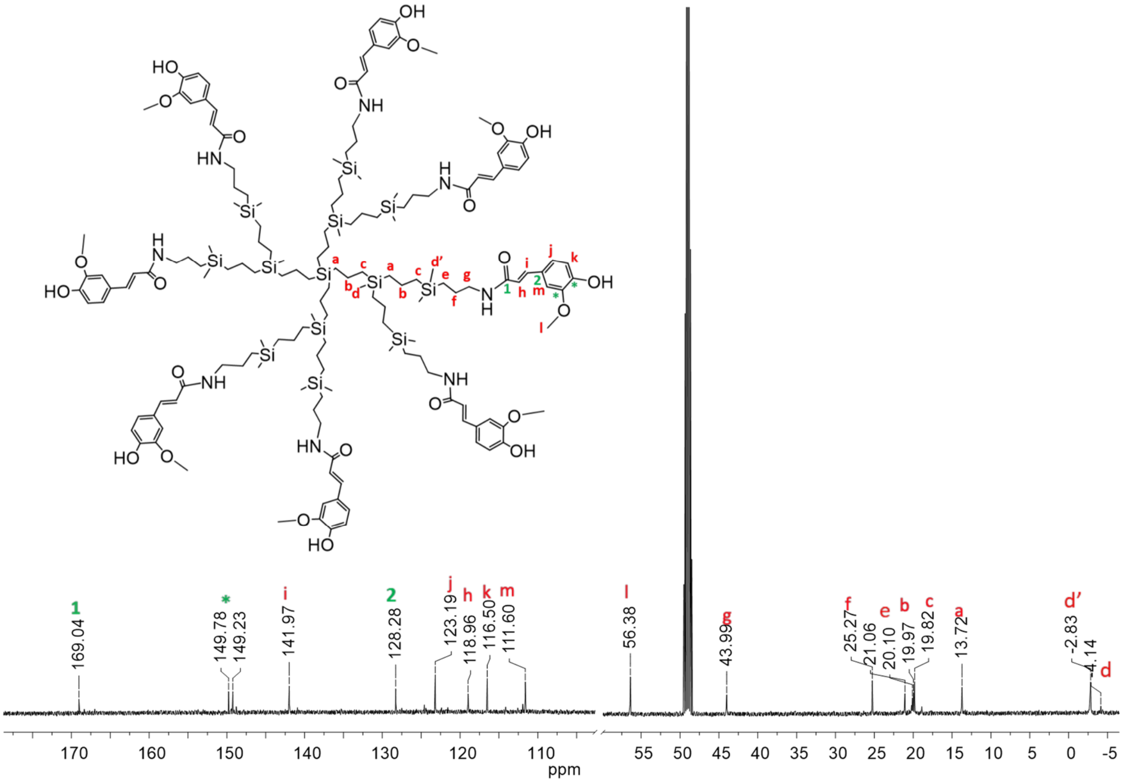

Figure S24. ^{13}C-NMR (500 MHz, CD_3OD) of dendritic polyphenol **(4)**

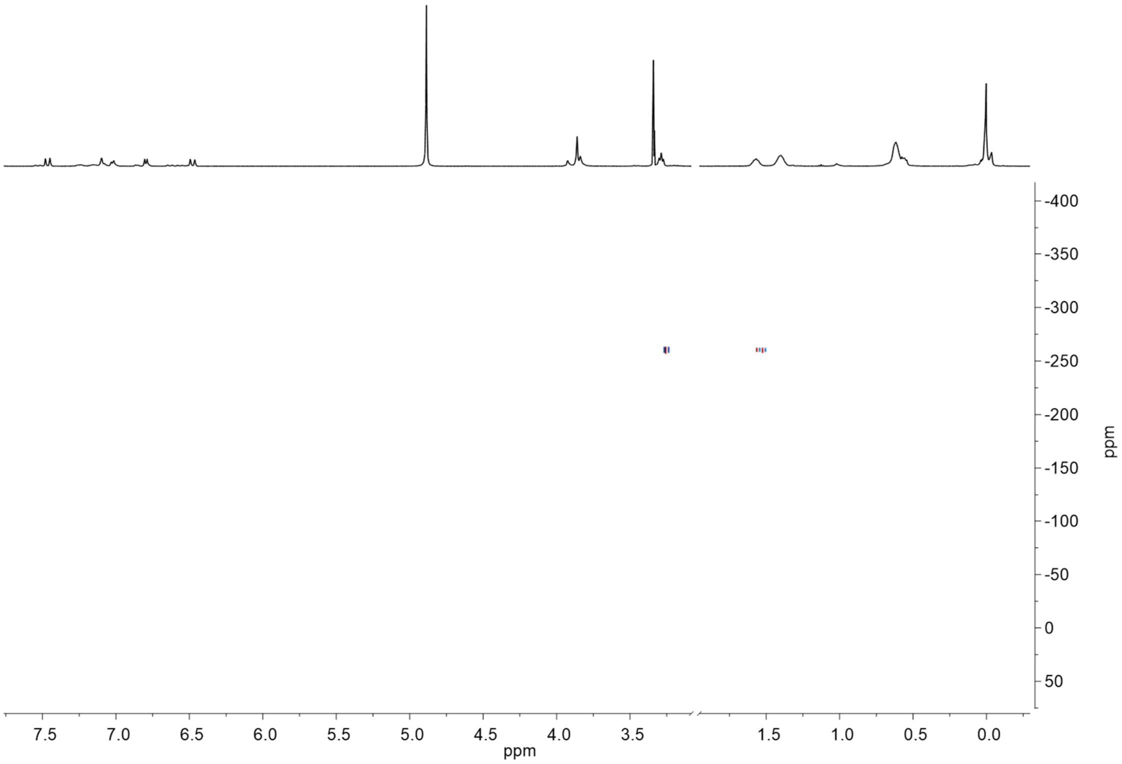

Figure S25. {^{1}H-^{15}N}-HMBC-NMR (500 MHz, CD_3OD) of dendritic polyphenol **(4)**

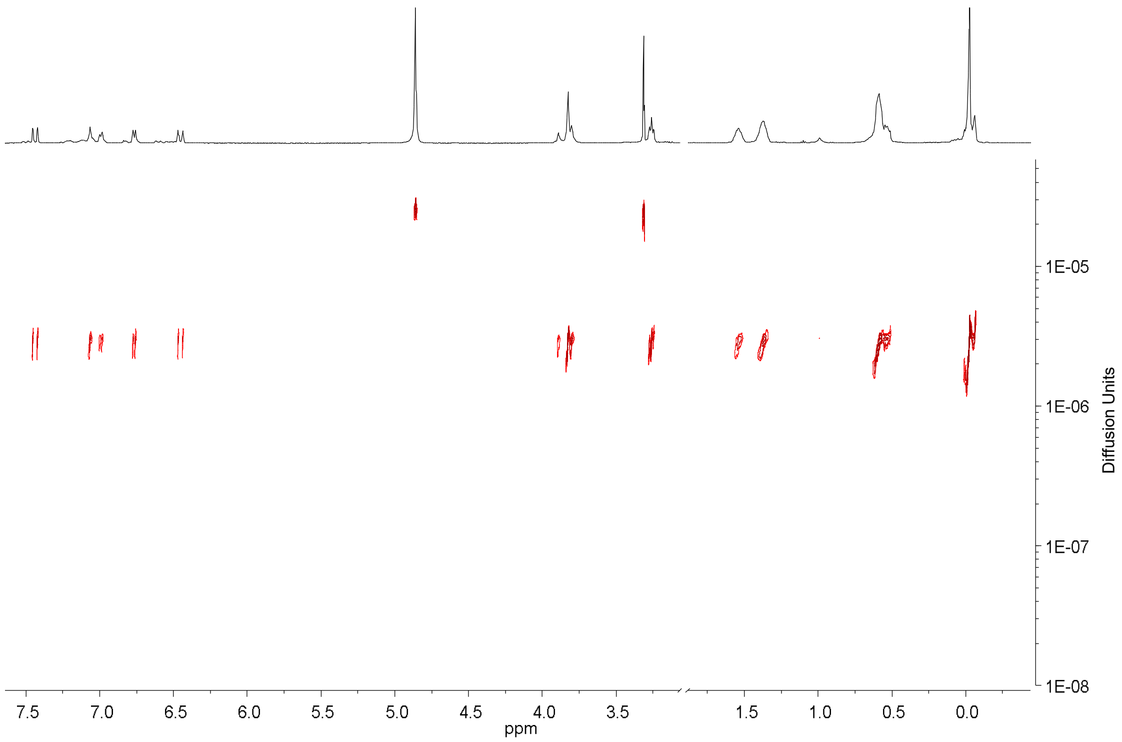

Figure S26. ^{1}H-DOSY-2D-NMR (500 MHz, CD_3OD) of dendritic polyphenol **(4)**

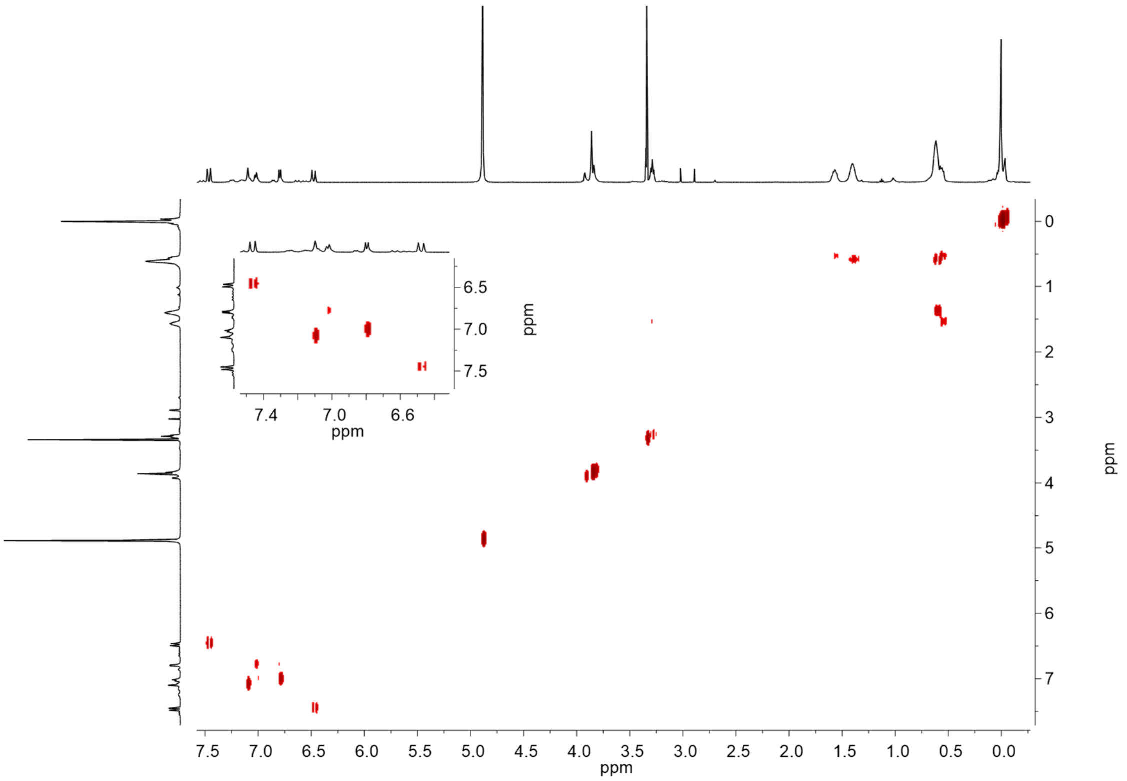

Figure S27. {^1H-^1H}-COSY-2D-NMR (500 MHz, CD_3OD) of dendritic polyphenol **(4)**

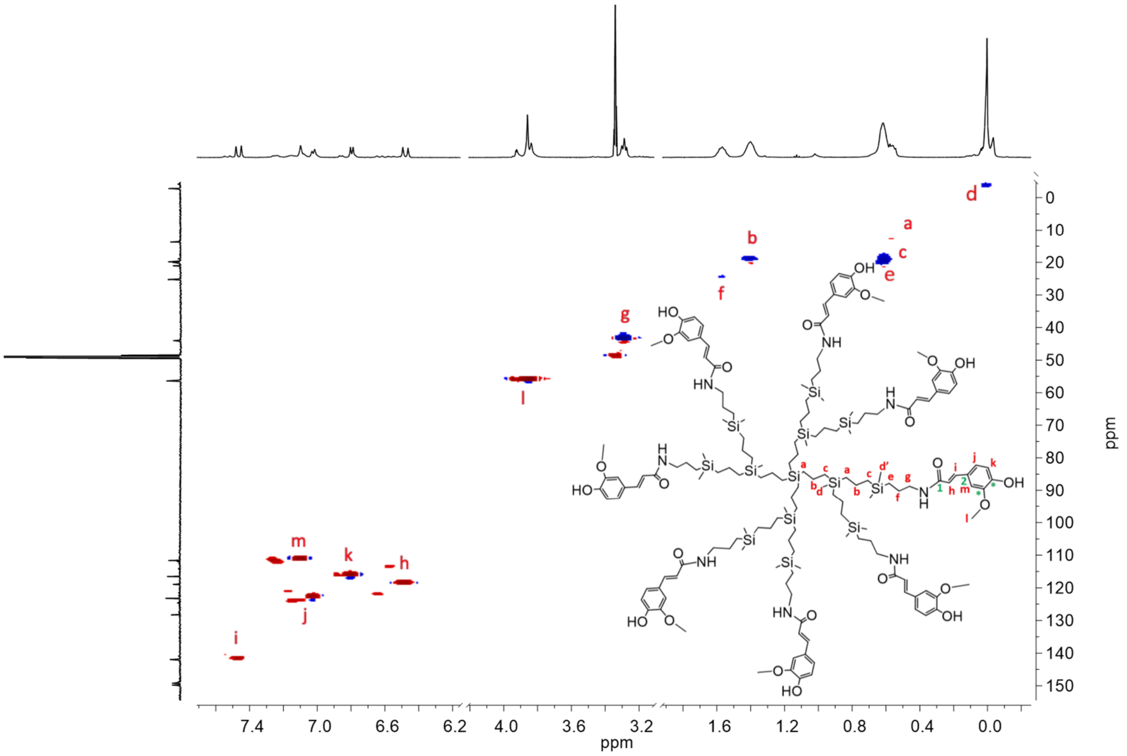

Figure S28. {^1H-^{13}C}-HSQC-2D-NMR (500 MHz, CD_3OD) of dendritic polyphenol **(4)**

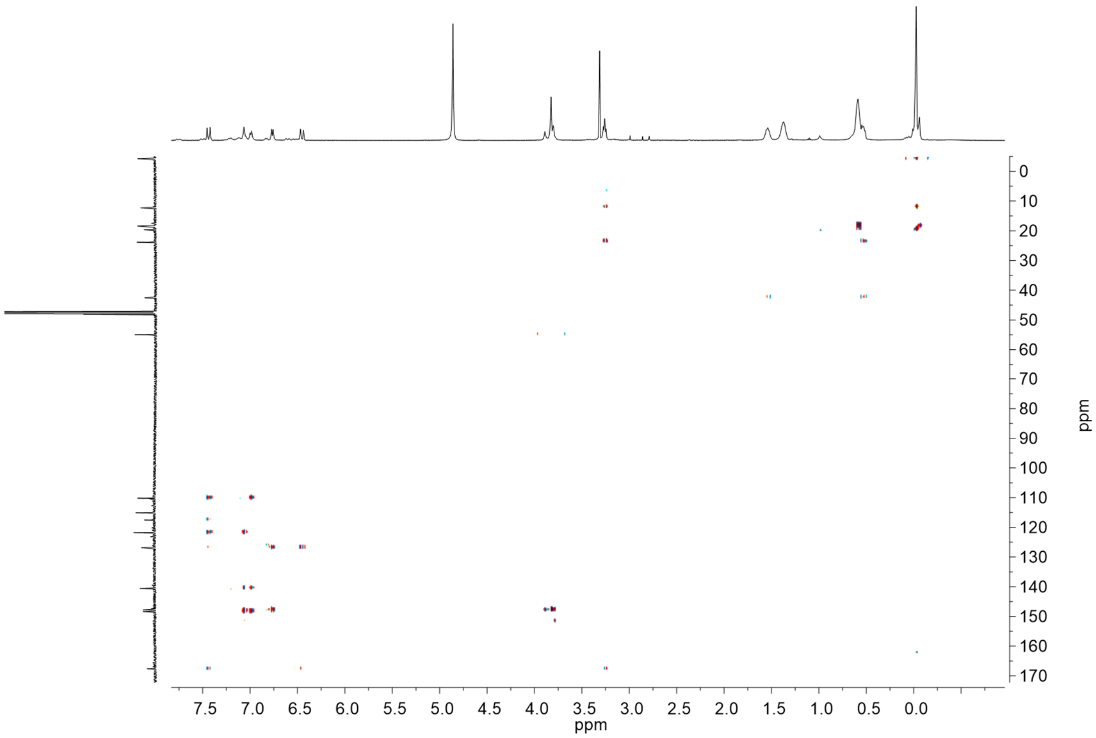

Figure S29. {^{1}H-^{13}C}-HMBC-2D-NMR (500 MHz, CD_3OD) of dendritic polyphenol **(4)**

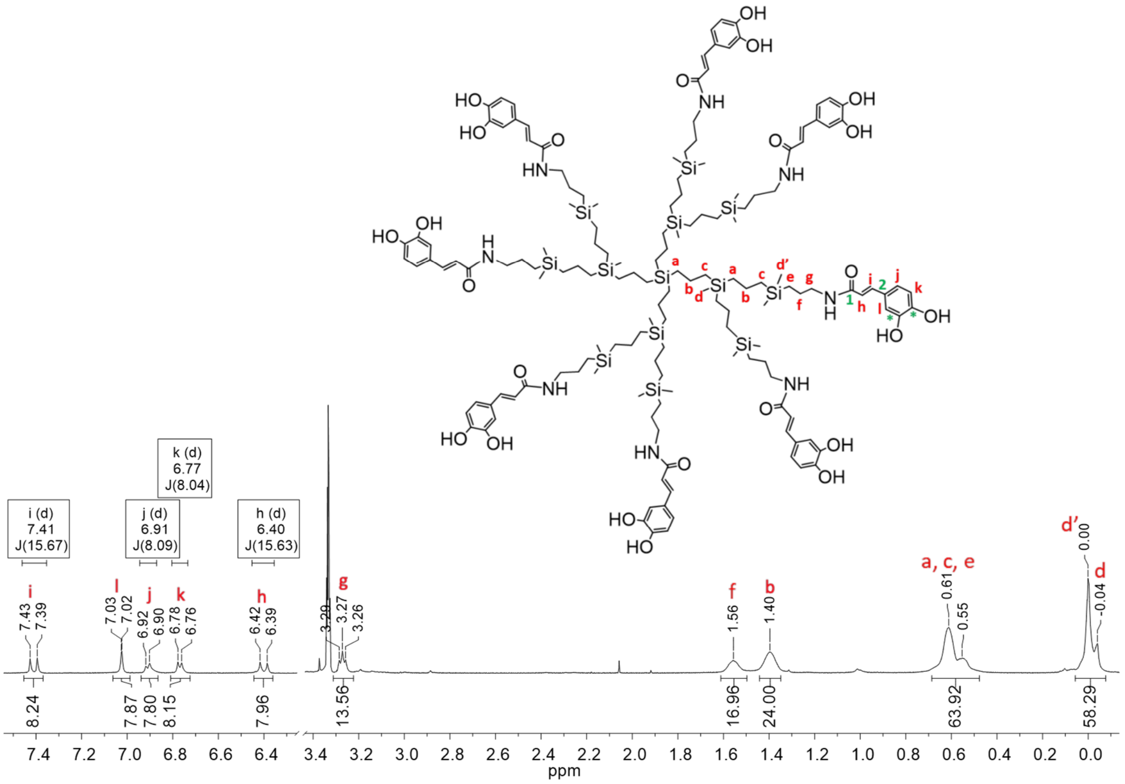

Figure S30. ^{1}H-NMR (500 MHz, CD_3OD) of dendritic polyphenol **(5)**

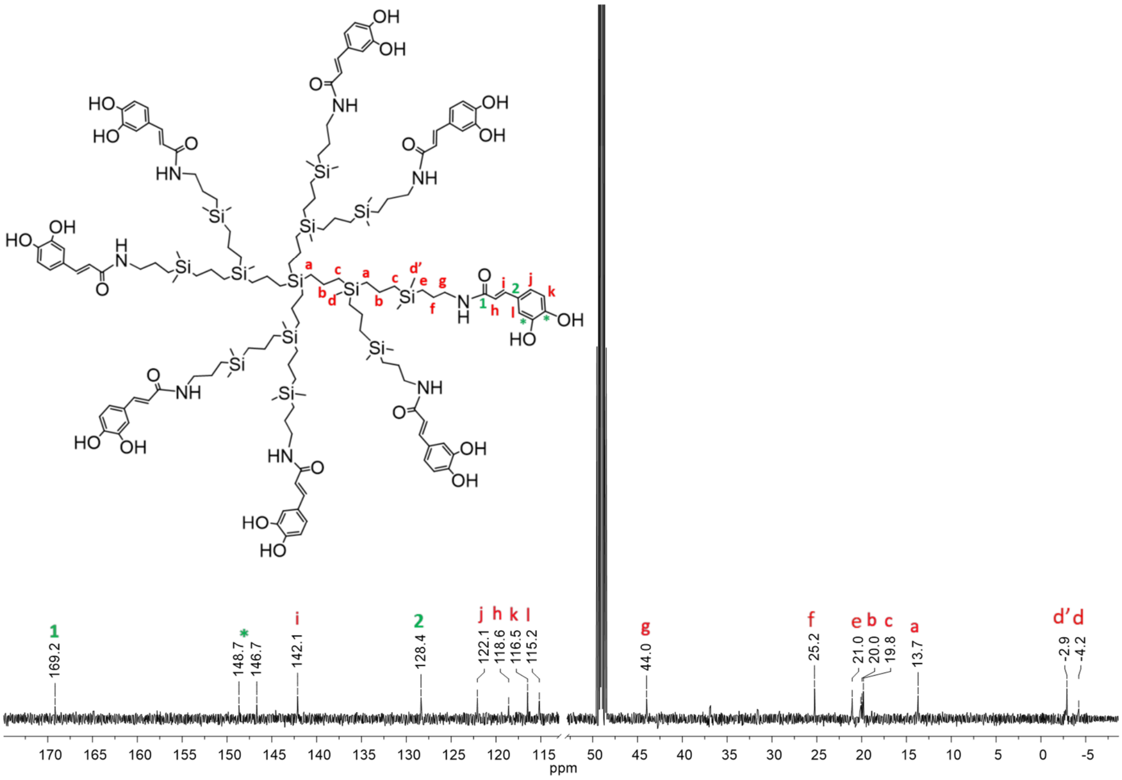

Figure S31. ^{13}C-NMR (500 MHz, CD_3OD) of dendritic polyphenol **(5)**

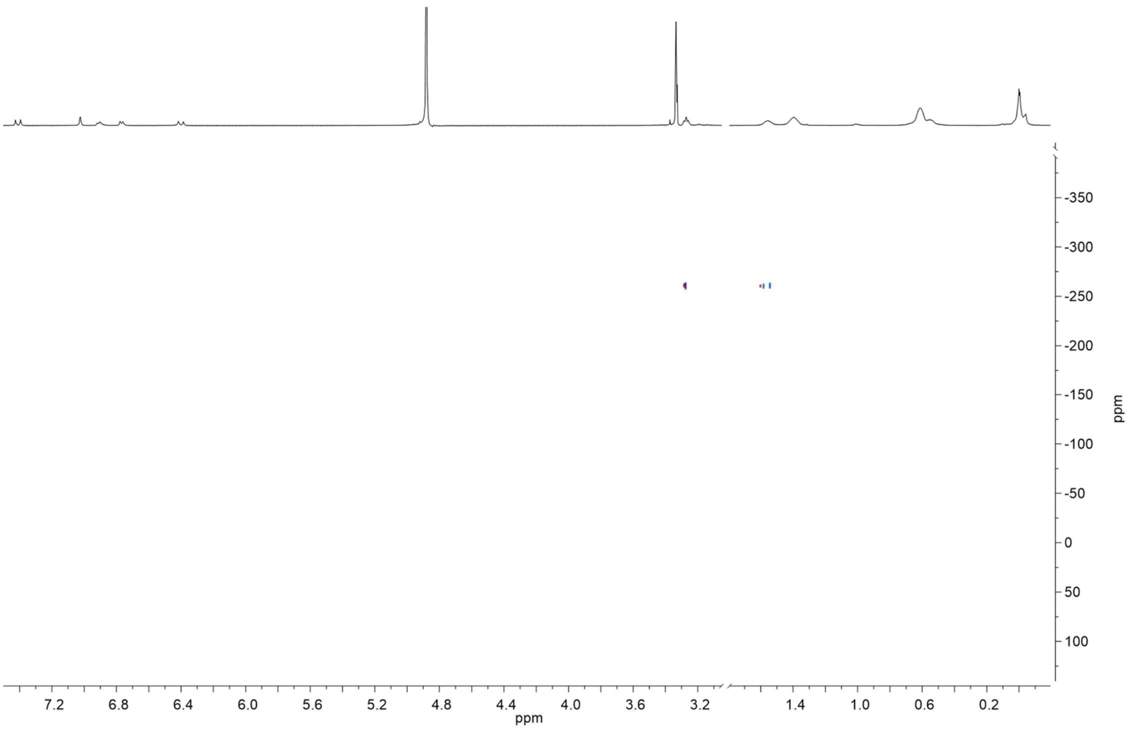

Figure S32. {^{1}H-^{15}N}-HMBC-NMR (500 MHz, CD_3OD) of dendritic polyphenol **(5)**

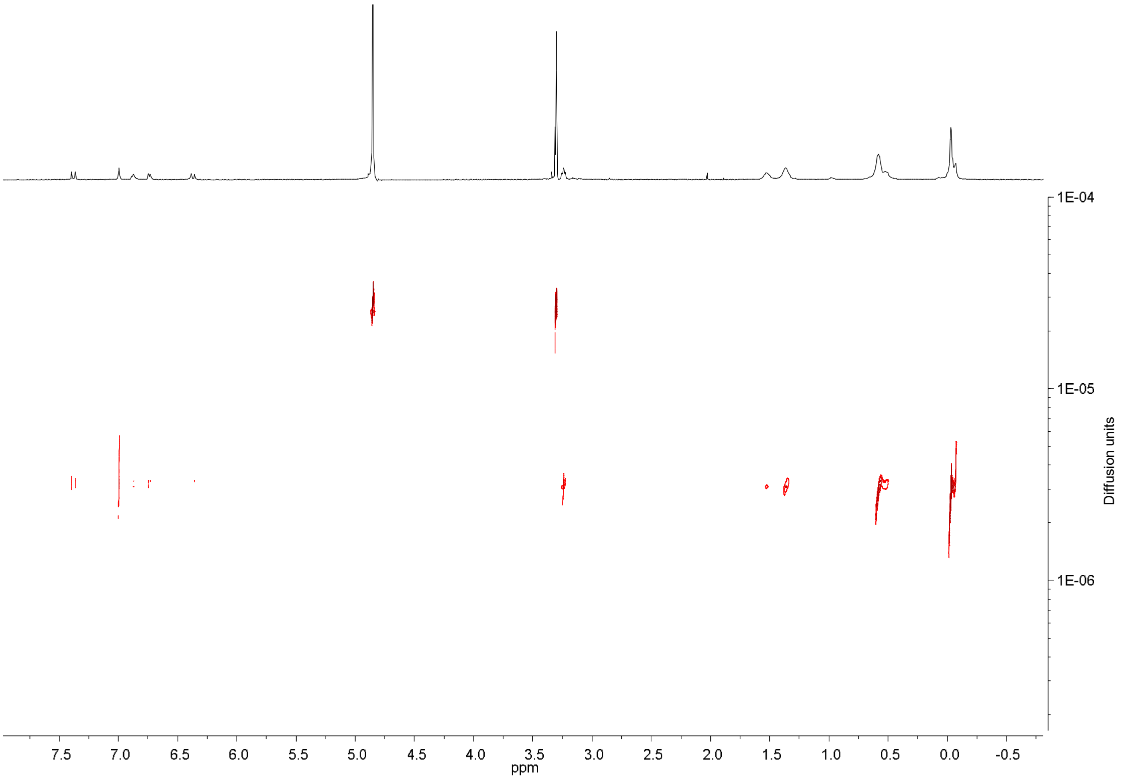

Figure S33. ^{1}H-DOSY-2D-NMR (500 MHz, CD_3OD) of dendritic polyphenol **(5)**

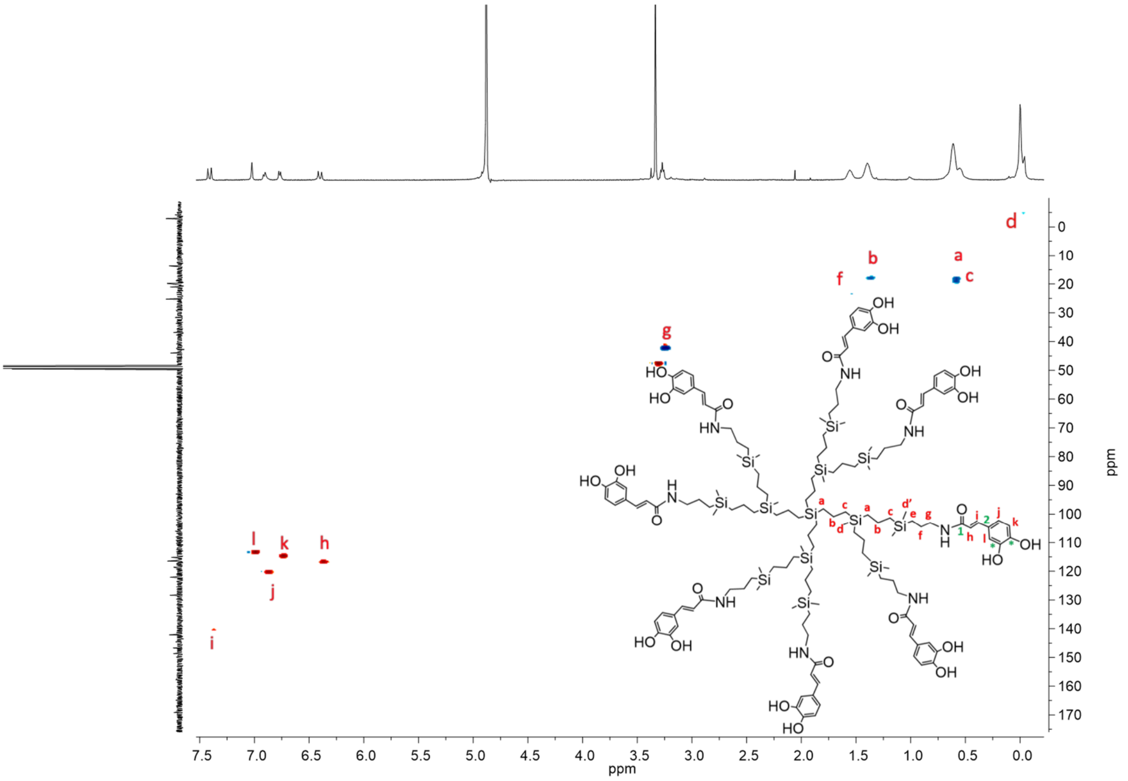

Figure S34. {^{1}H-^{13}C}-HSQC-2D-NMR (500 MHz, CD_3OD) of dendritic polyphenol **(5)**

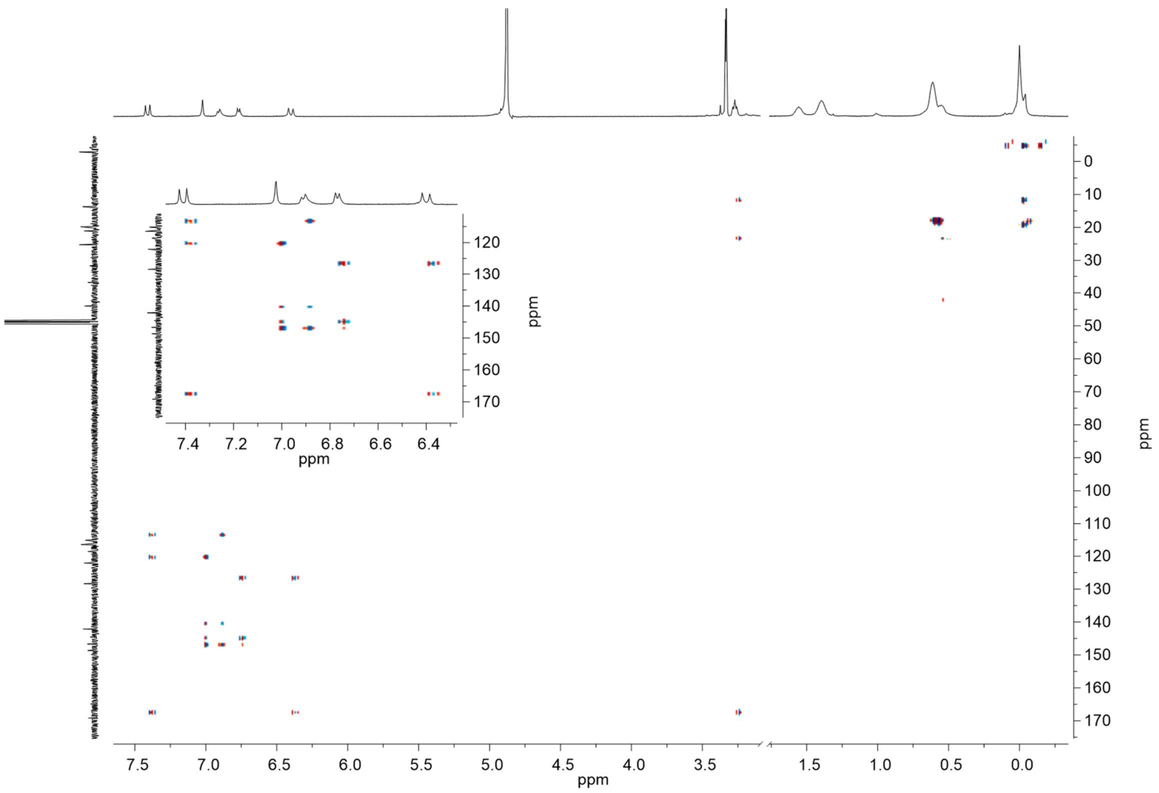

Figure S35. {^{1}H-^{13}C}-HMBC-2D-NMR (500 MHz, CD_3OD) of dendritic polyphenol **(5)**

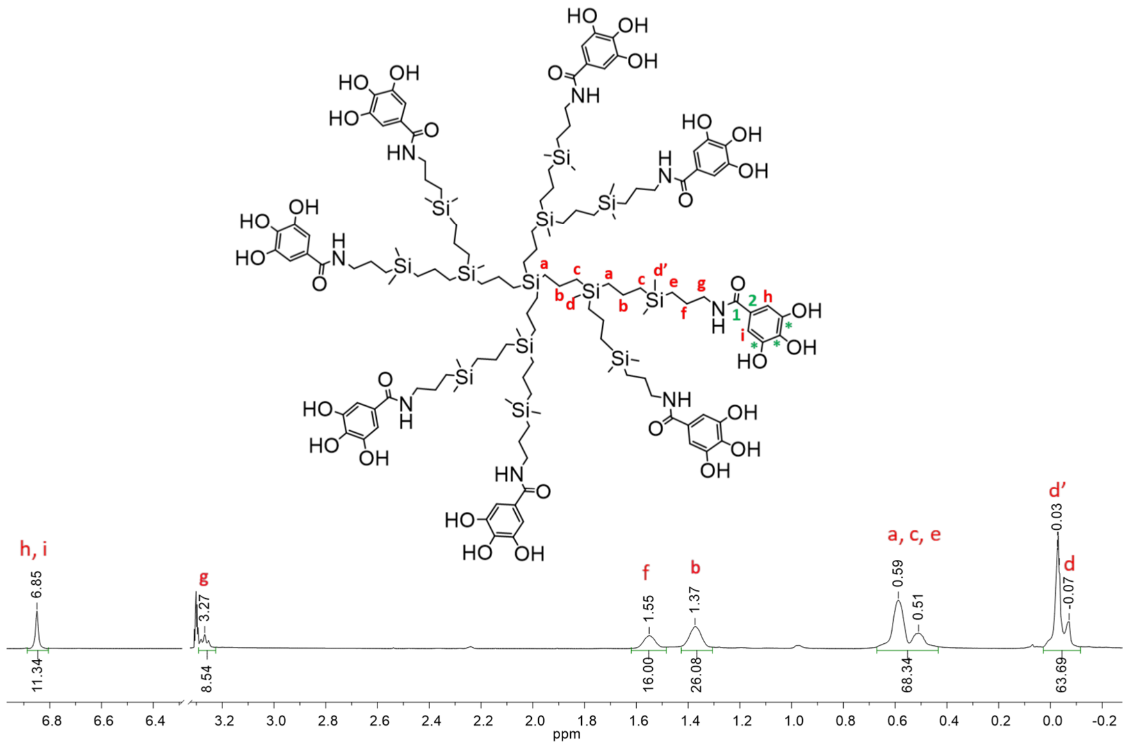

Figure S36. ^{1}H-NMR (500 MHz, CD_3OD) of dendritic polyphenol **(6)**

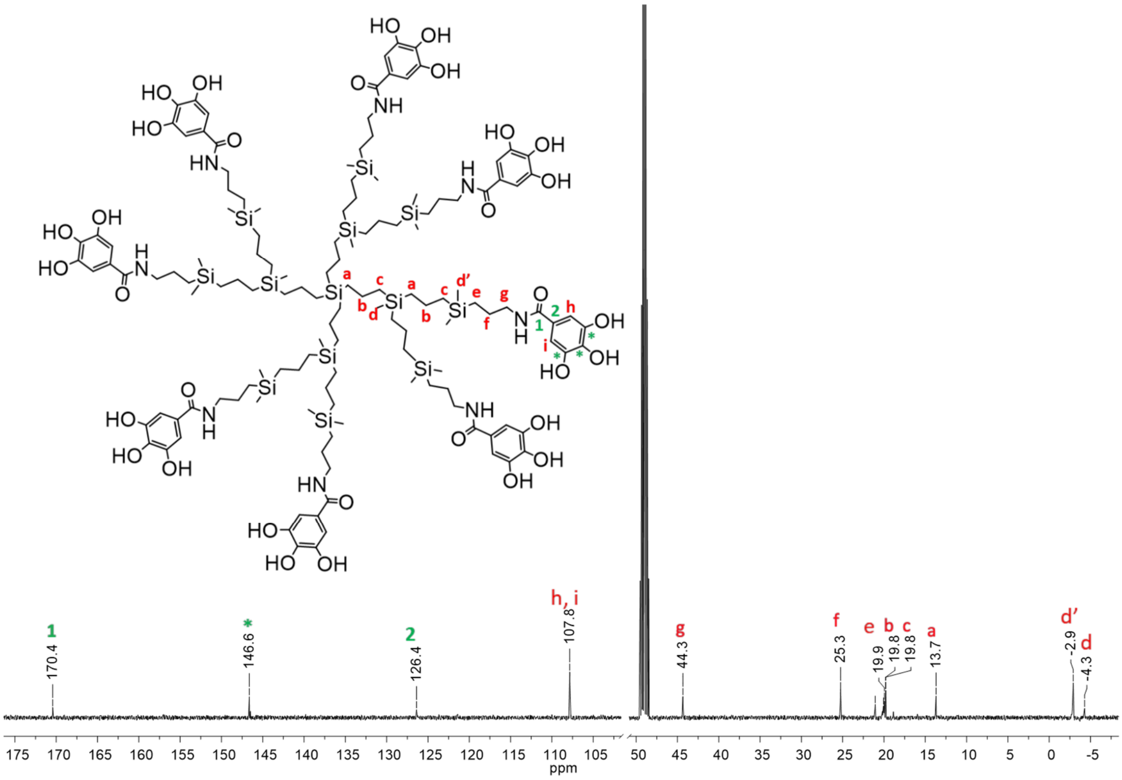

Figure S37. ^{13}C-NMR (500 MHz, CD_3OD) of dendritic polyphenol **(6)**

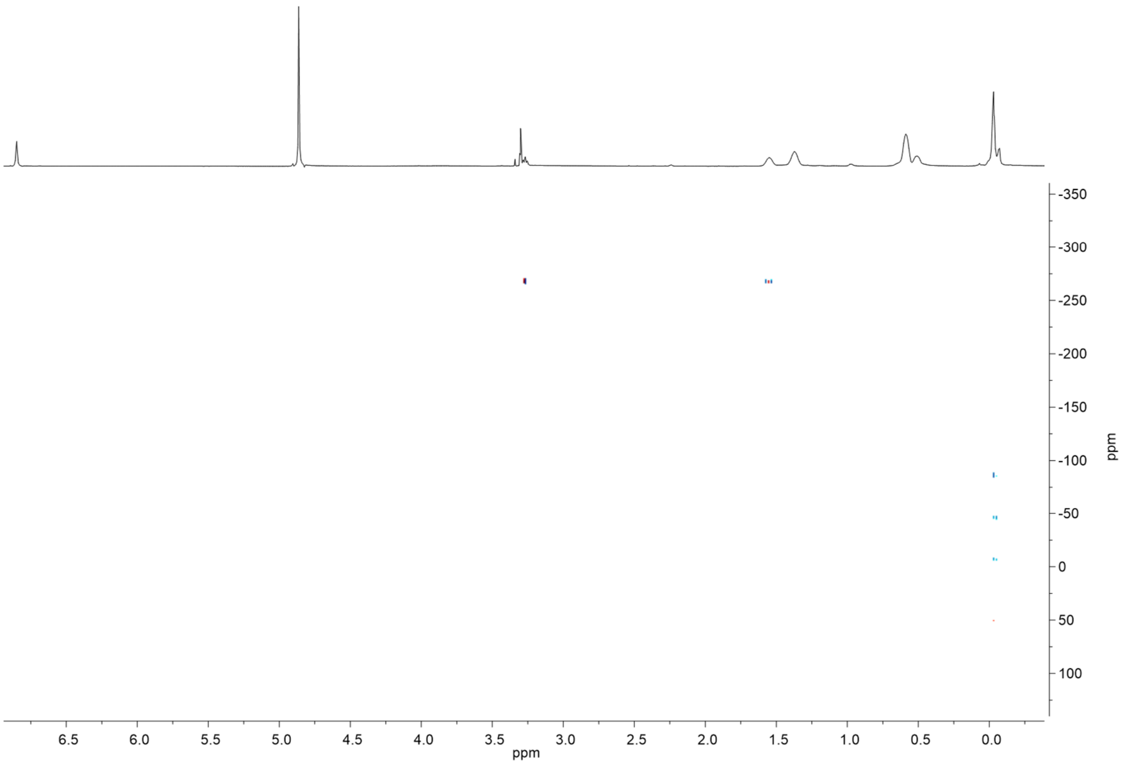

Figure S38. {^{1}H-^{15}N}-HMBC-NMR (500 MHz, CD_3OD) of dendritic polyphenol **(6)**

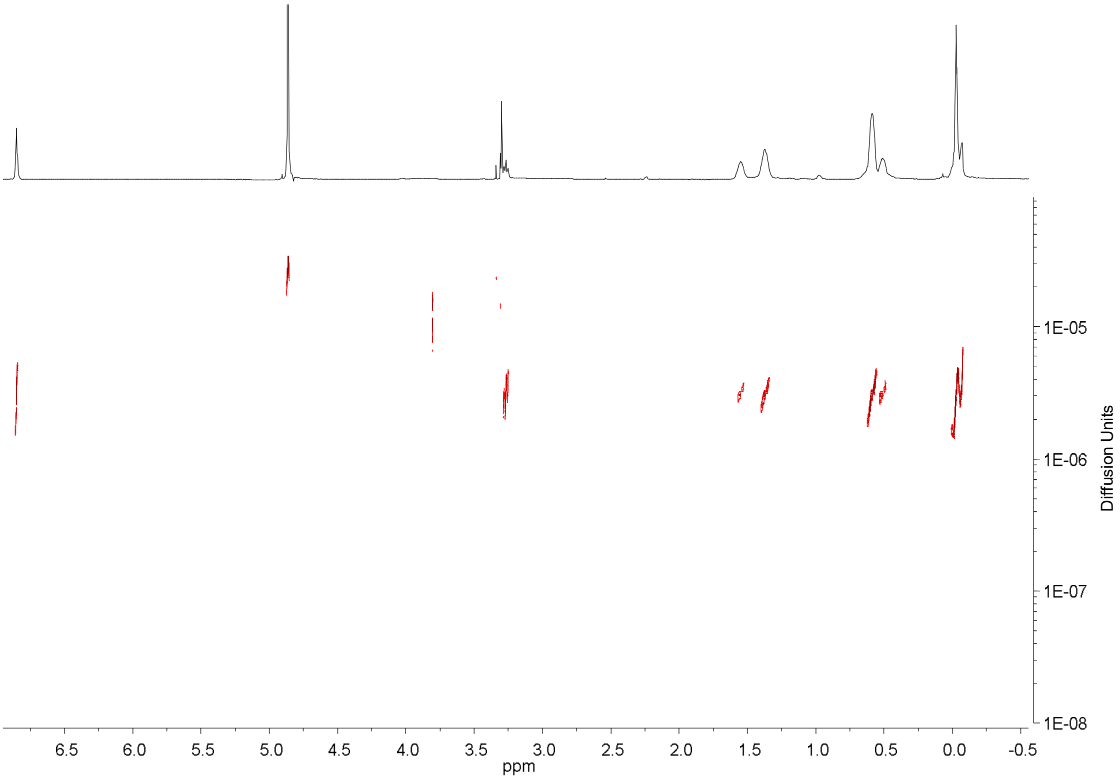

Figure S39. ^{1}H-DOSY-2D-NMR (500 MHz, CD_3OD) of dendritic polyphenol **(6)**

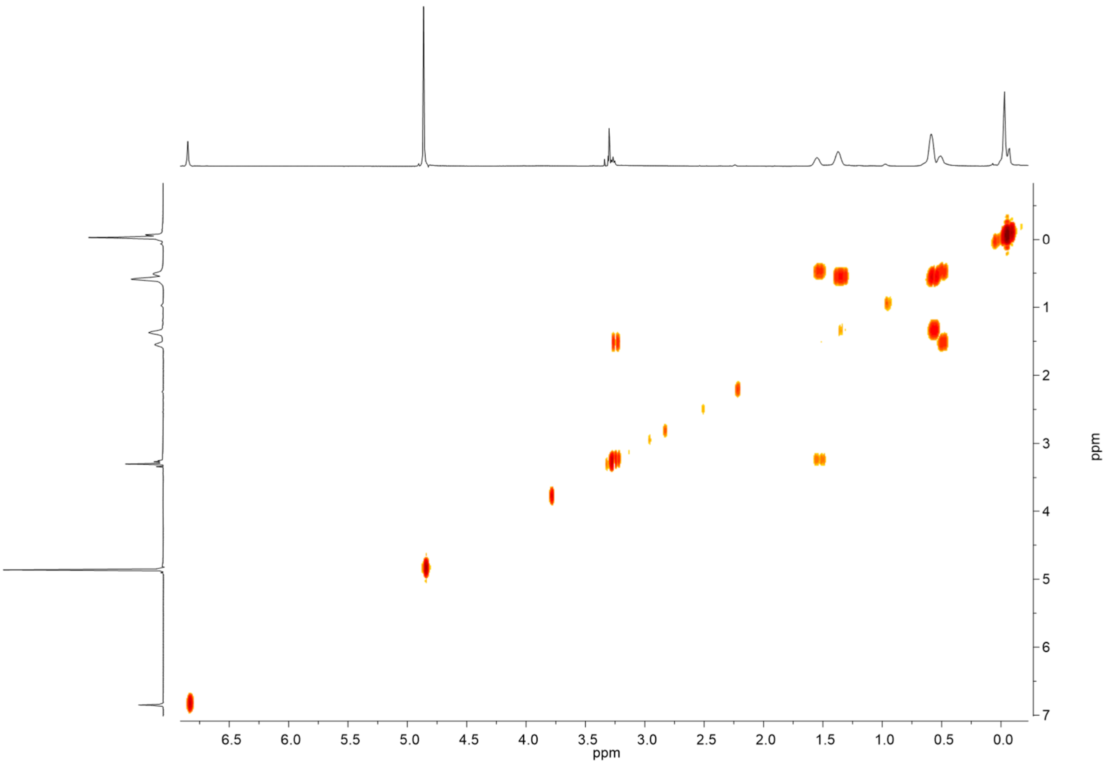

Figure S40. {^{1}H-^{1}H}-COSY-2D-NMR (500 MHz, CD_3OD) of dendritic polyphenol **(6)**

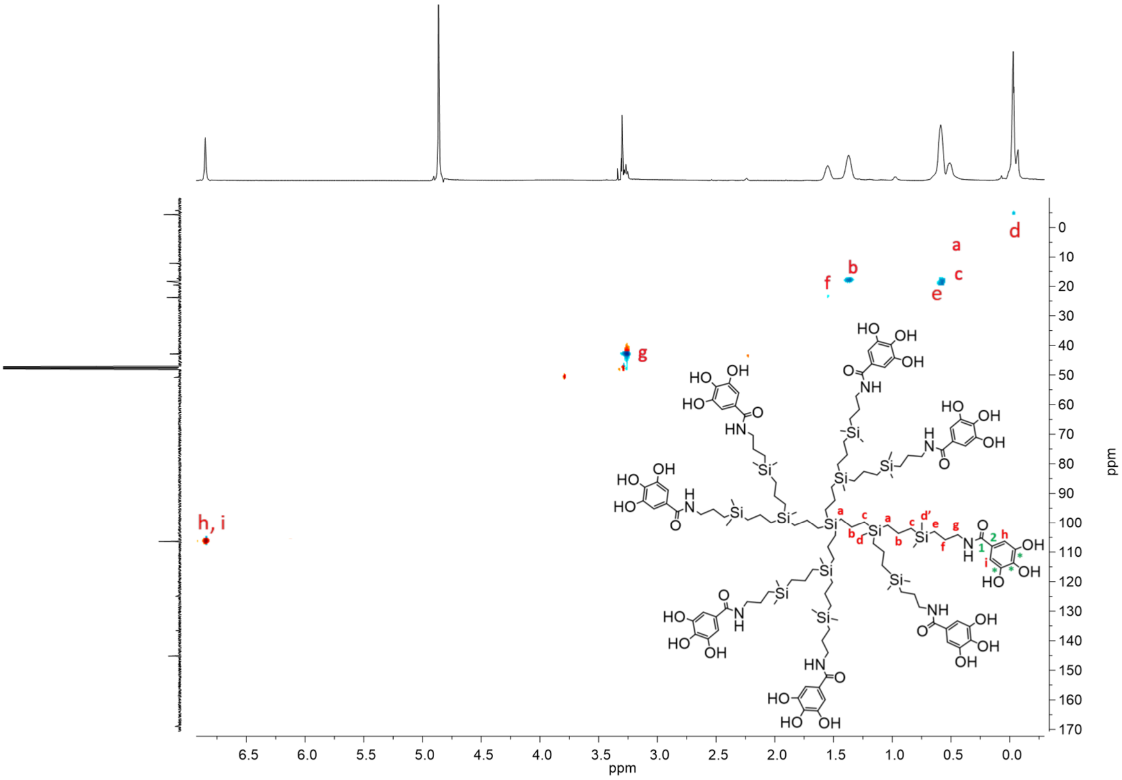

Figure S41. {^{1}H-^{13}C}-HSQC-2D-NMR (500 MHz, CD_3OD) of dendritic polyphenol **(6)**

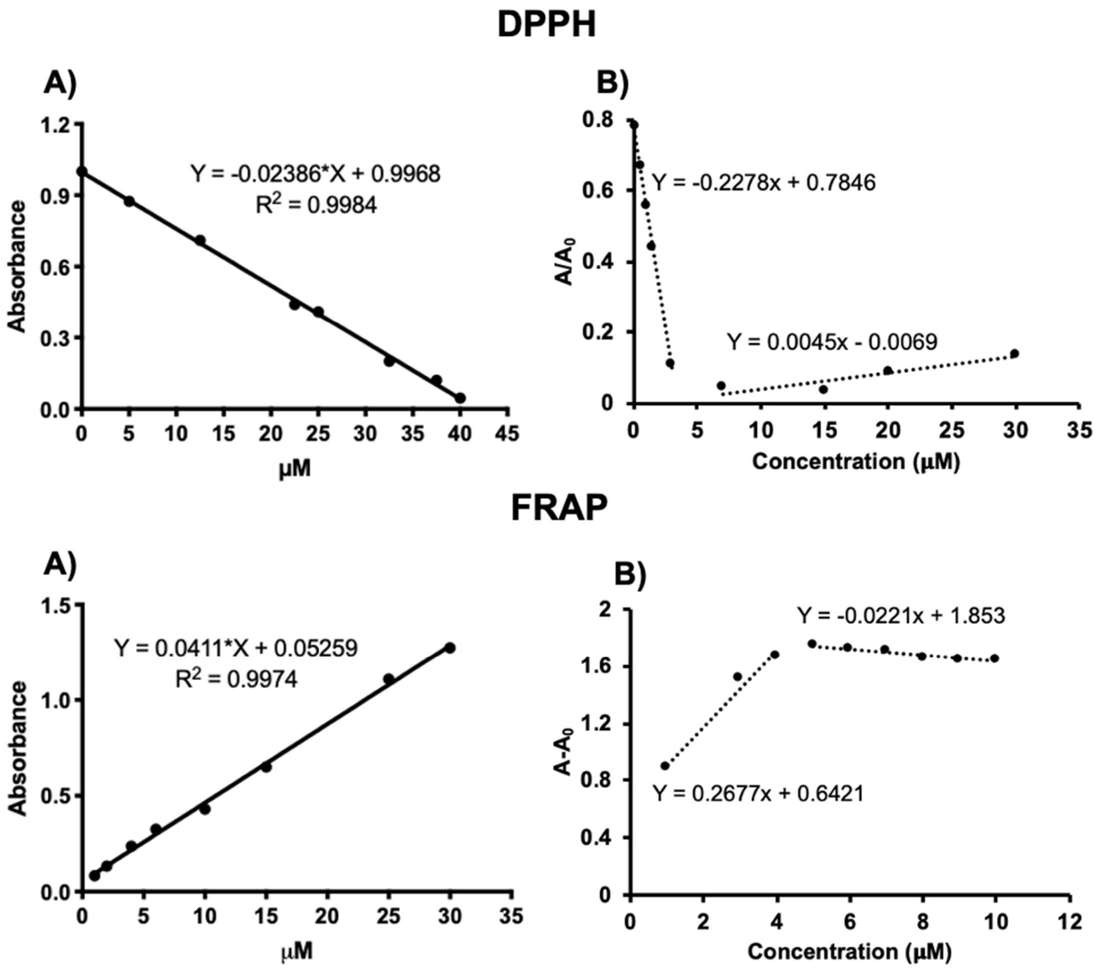

Figure S42. A) A representative calibration curve of inhibition of DPPH by Trolox standards. Representative results of at least three independent experiments are shown. B) Graphics with equations line for compound G_1-$[Si(CH_2)_3NH(CO)Ph(OH)_3]_4$ **(3)**.
