# Supplementary material for: Erratum: Sanz del Olmo, N.; et al. Antioxidant and Antibacterial Properties of Carbosilane Dendrimers Functionalized with Polyphenolic Moieties. Pharmaceutics 2020, 12, 698
Source: Pharmaceutics. 2021 Jan 19;13(1):121. doi: 10.3390/pharmaceutics13010121 (PMC7835782; doi:10.3390/pharmaceutics13010121)
Supplement: Supplementary file 1 [file pharmaceutics-13-00121-s001.pdf]

pharmaceutics

Supplementary Materials: Erratum: Sanz del Olmo, N.; et al. Antioxidant and Antibacterial Properties of Carbosilane Dendrimers Functionalized with Polyphenolic Moieties. *Pharmaceutics* 2020, *12*, 698

Natalia Sanz del Olmo, Cornelia E. Peña González, Daniel Rojas, Rafael Gómez, Paula Ortega *, Alberto Escarpa and Francisco Javier de la Mata *

Table of contents

Figure S1. Mass Spectrometry (ESI-TOF) of dendritic polyphenol (1)

Figure S2. ^{1}H-NMR (500 MHz, CD_3OD) of dendritic polyphenol (1)

Figure S3. ^{13}C-NMR (500 MHz, CD_3OD) of dendritic polyphenol (1)

Figure S4. {^{1}H-^{15}N}-HMBC-NMR (500 MHz, CD_3OD) of dendritic polyphenol (1)

Figure S5. ^{1}H-DOSY-2D-NMR (500 MHz, CD_3OD) of dendritic polyphenol (1)

Figure S6. {^{1}H-^{1}H}-COSY-2D-NMR (500 MHz, CD_3OD) of dendritic polyphenol (1)

Figure S7. {^{1}H-^{13}C}-HSQC-2D-NMR (500 MHz, CD_3OD) of dendritic polyphenol (1)

Figure S8. {^{1}H-^{13}C}-HMBC-2D-NMR (500 MHz, CD_3OD) of dendritic polyphenol (1)

Figure S9. Mass Spectrometry (ESI-TOF) of dendritic polyphenol (2)

Figure S10. ^{1}H-NMR (500 MHz, CD_3OD) of dendritic polyphenol (2)

Figure S11. ^{13}C-NMR (500 MHz, CD_3OD) of dendritic polyphenol (2)

Figure S12. {^{1}H-^{15}N}-HMBC-NMR (500 MHz, CD_3OD) of dendritic polyphenol (2)

Figure S13. ^{1}H-DOSY-2D-NMR (500 MHz, CD_3OD) of dendritic polyphenol (2)

Figure S14. {^{1}H-^{1}H}-COSY-2D-NMR (500 MHz, CD_3OD) of dendritic polyphenol (2)

Figure S15. {^{1}H-^{13}C}-HSQC-2D-NMR (500 MHz, CD_3OD) of dendritic polyphenol (2)

Figure S16. Mass Spectrometry (ESI-TOF) of dendritic polyphenol (3)

Figure S17. ^{1}H-NMR (500 MHz, CD_3OD) of dendritic polyphenol (3)

Figure S18. ^{13}C-NMR (500 MHz, CD_3OD) of dendritic polyphenol (3)

Figure S19. {^{1}H-^{15}N}-HMBC-NMR (500 MHz, CD_3OD) of dendritic polyphenol (3)

Figure S20. ^{1}H-DOSY-2D-NMR (500 MHz, CD_3OD) of dendritic polyphenol (3)

Figure S21. {^{1}H-^{1}H}-COSY-2D-NMR (500 MHz, CD_3OD) of dendritic polyphenol (3)

Figure S22. {^{1}H-^{13}C}-HSQC-2D-NMR (500 MHz, CD_3OD) of dendritic polyphenol (3)

Figure S23. ^{1}H-NMR (500 MHz, CD_3OD) of dendritic polyphenol (4)

Figure S24. ^{13}C-NMR (500 MHz, CD_3OD) of dendritic polyphenol (4)

Figure S25. {^{1}H-^{15}N}-HMBC-NMR (500 MHz, CD_3OD) of dendritic polyphenol (4)

Figure S26. ^{1}H-DOSY-2D-NMR (500 MHz, CD_3OD) of dendritic polyphenol (4)

Figure S27. {^{1}H-^{1}H}-COSY-2D-NMR (500 MHz, CD_3OD) of dendritic polyphenol (4)

Figure S28. {^{1}H-^{13}C}-HSQC-2D-NMR (500 MHz, CD_3OD) of dendritic polyphenol (4)

Figure S29. {^{1}H-^{13}C}-HMBC-2D-NMR (500 MHz, CD_3OD) of dendritic polyphenol (4)

Figure S30. ^{1}H-NMR (500 MHz, CD_3OD) of dendritic polyphenol (5)

Figure S31. ^{13}C-NMR (500 MHz, CD_3OD) of dendritic polyphenol (5)

Figure S32. {^{1}H-^{15}N}-HMBC-NMR (500 MHz, CD_3OD) of dendritic polyphenol (5)

Figure S33. ^{1}H-DOSY-2D-NMR (500 MHz, CD_3OD) of dendritic polyphenol (5)

Figure S34. {^{1}H-^{13}C}-HSQC-2D-NMR (500 MHz, CD_3OD) of dendritic polyphenol (5)

Figure S35. {^{1}H-^{13}C}-HMBC-2D-NMR (500 MHz, CD_3OD) of dendritic polyphenol (5)

Figure S36. ^{1}H-NMR (500 MHz, CD_3OD) of dendritic polyphenol (6)

Figure S37. ^{13}C-NMR (500 MHz, CD_3OD) of dendritic polyphenol (6)

Figure S38. {^{1}H-^{15}N}-HMBC-NMR (500 MHz, CD_3OD) of dendritic polyphenol (6)

Figure S39. ^{1}H-DOSY-2D-NMR (500 MHz, CD_3OD) of dendritic polyphenol (6)

Figure S40. {^{1}H-^{1}H}-COSY-2D-NMR (500 MHz, CD_3OD) of dendritic polyphenol (6)

Figure S41. {^{1}H-^{13}C}-HSQC-2D-NMR (500 MHz, CD_3OD) of dendritic polyphenol (6)

Figure S42. A) A representative calibration curve of inhibition of DPPH by Trolox standards. Representative results of at least three independent experiments are shown. B) Graphics with equations line for compound G_1-[Si$(CH_2)_3$NH(CO)Ph$(OH)_3$]$_4$ (3).

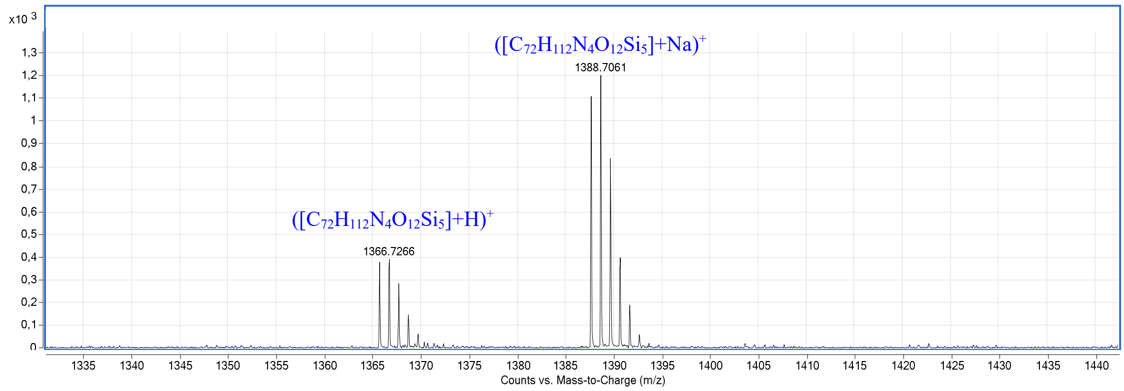

Figure S1. Mass Spectrometry (ESI-TOF) of dendritic polyphenol **(1)**.

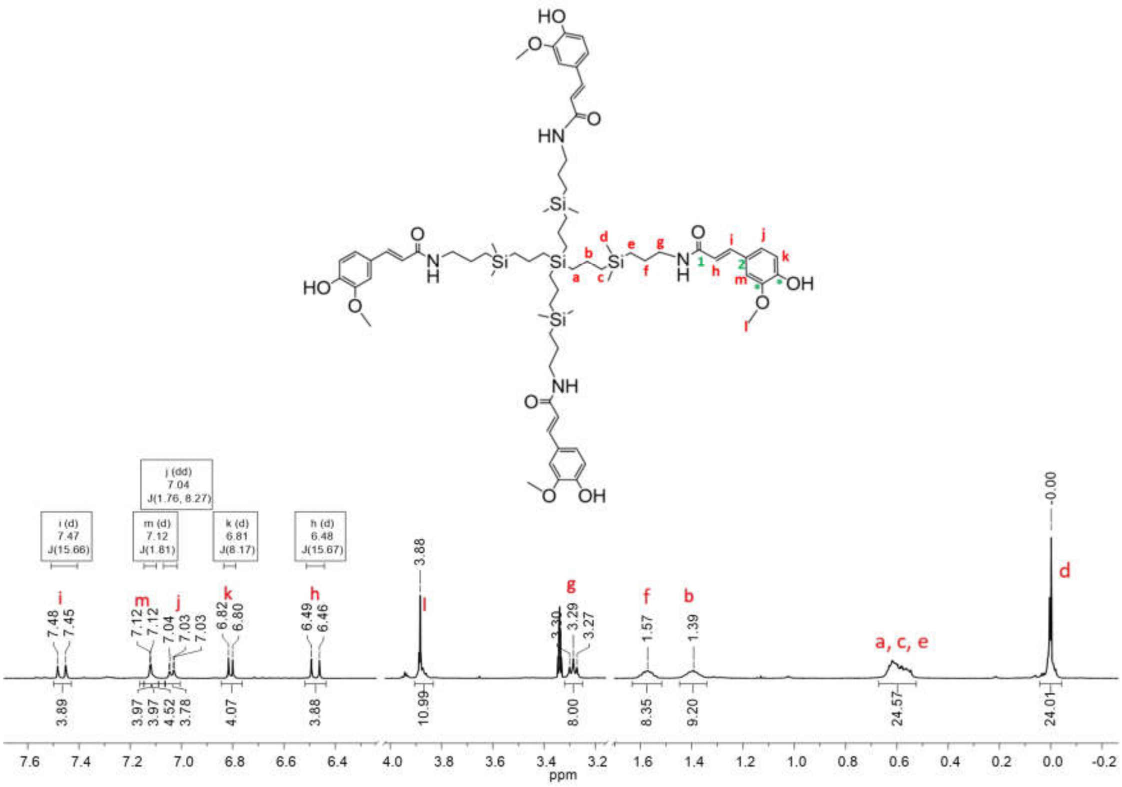

Figure S2. ^{1}H-NMR (500 MHz, CD_3OD) of dendritic polyphenol **(1)**.

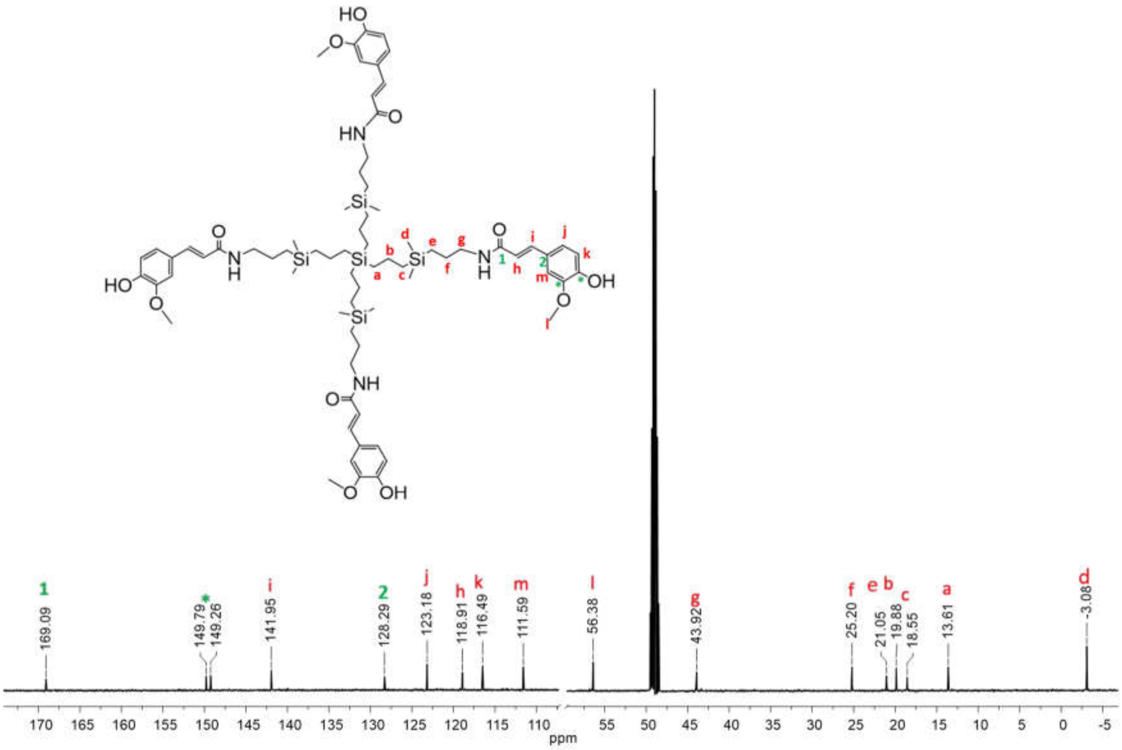

Figure S3. ^{13}C-NMR (500 MHz, CD_3OD) of dendritic polyphenol **(1)**.

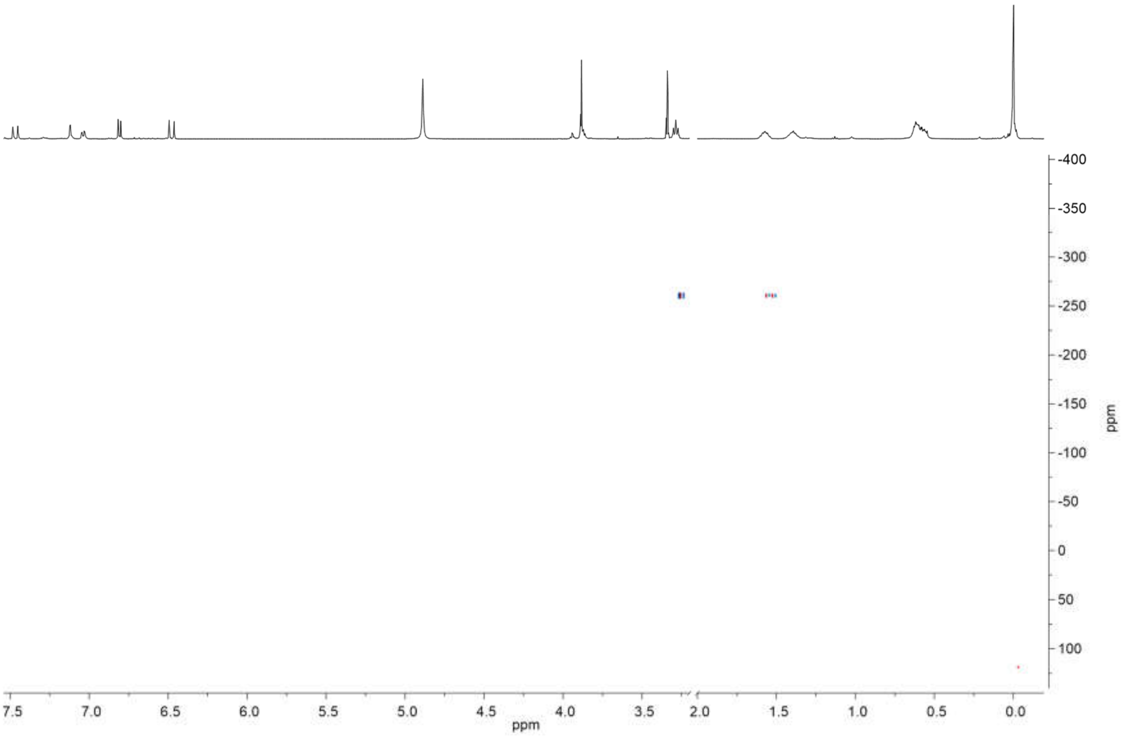

Figure S4. {^{1}H-^{15}N}-HMBC-NMR (500 MHz, CD_3OD) of dendritic polyphenol **(1)**.

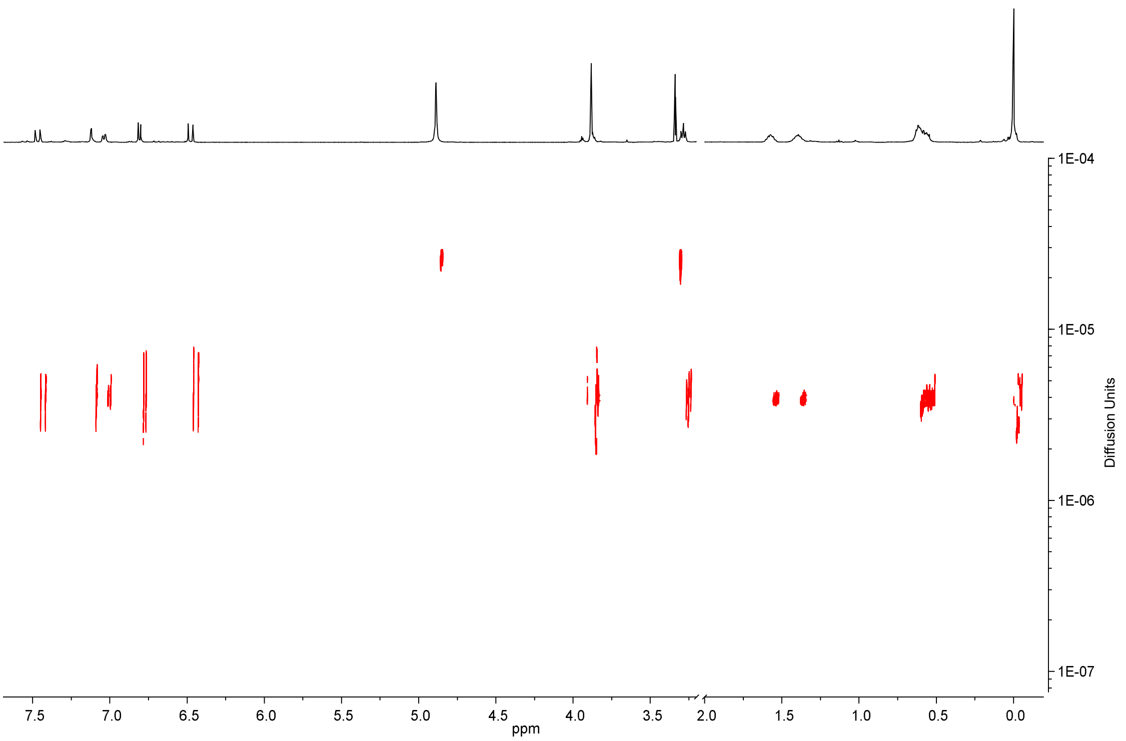

Figure S5. ^{1}H-DOSY-2D-NMR (500 MHz, CD_3OD) of dendritic polyphenol **(1)**.

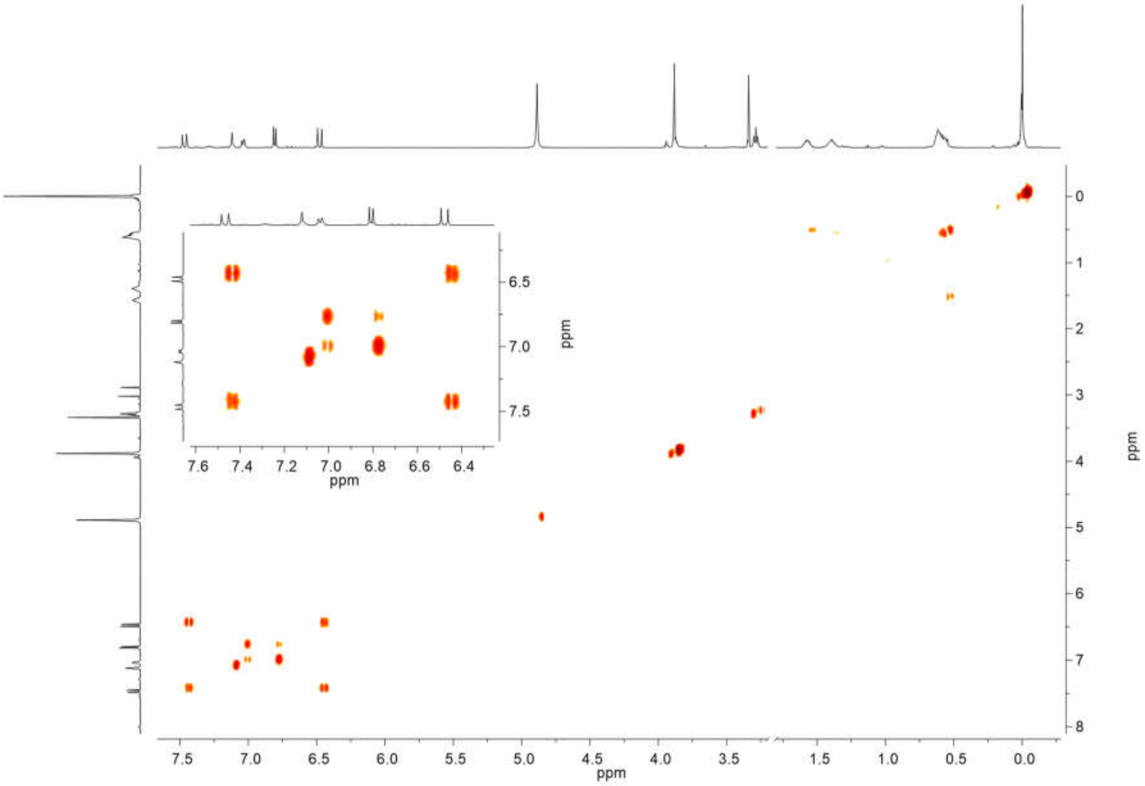

Figure S6. {^{1}H-^{1}H}-COSY-2D-NMR (500 MHz, CD_3OD) of dendritic polyphenol **(1)**.

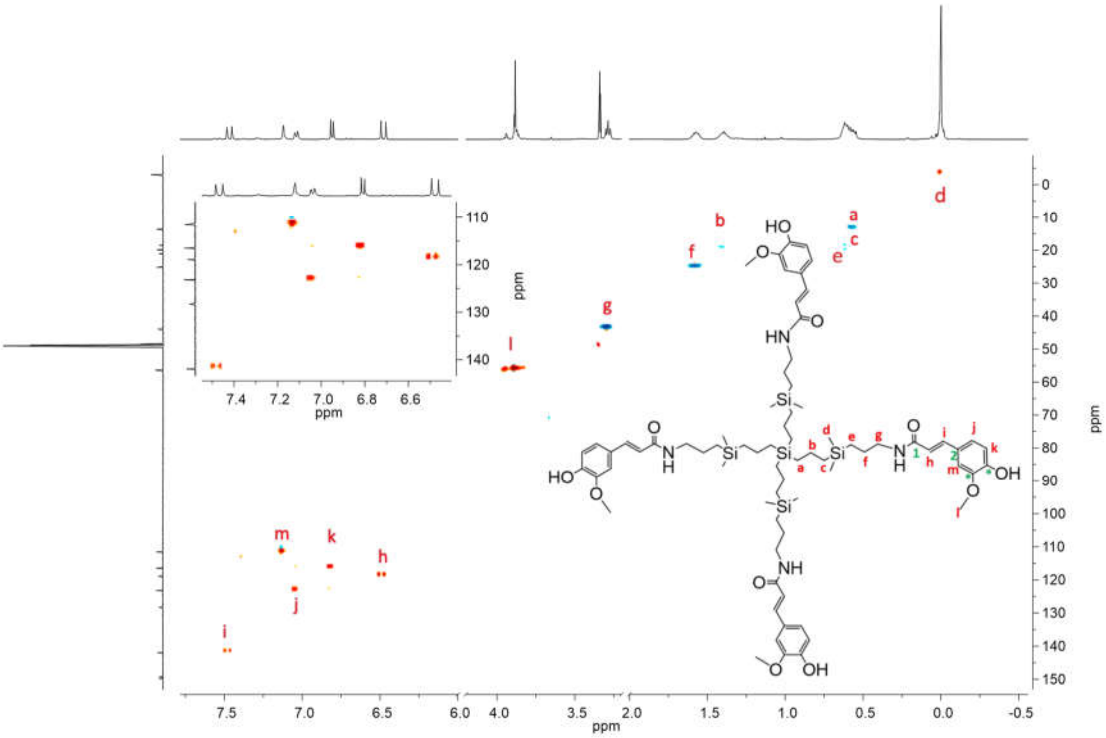

Figure S7. {^{1}H-^{13}C}-HSQC-2D-NMR (500 MHz, CD_3OD) of dendritic polyphenol **(1)**.

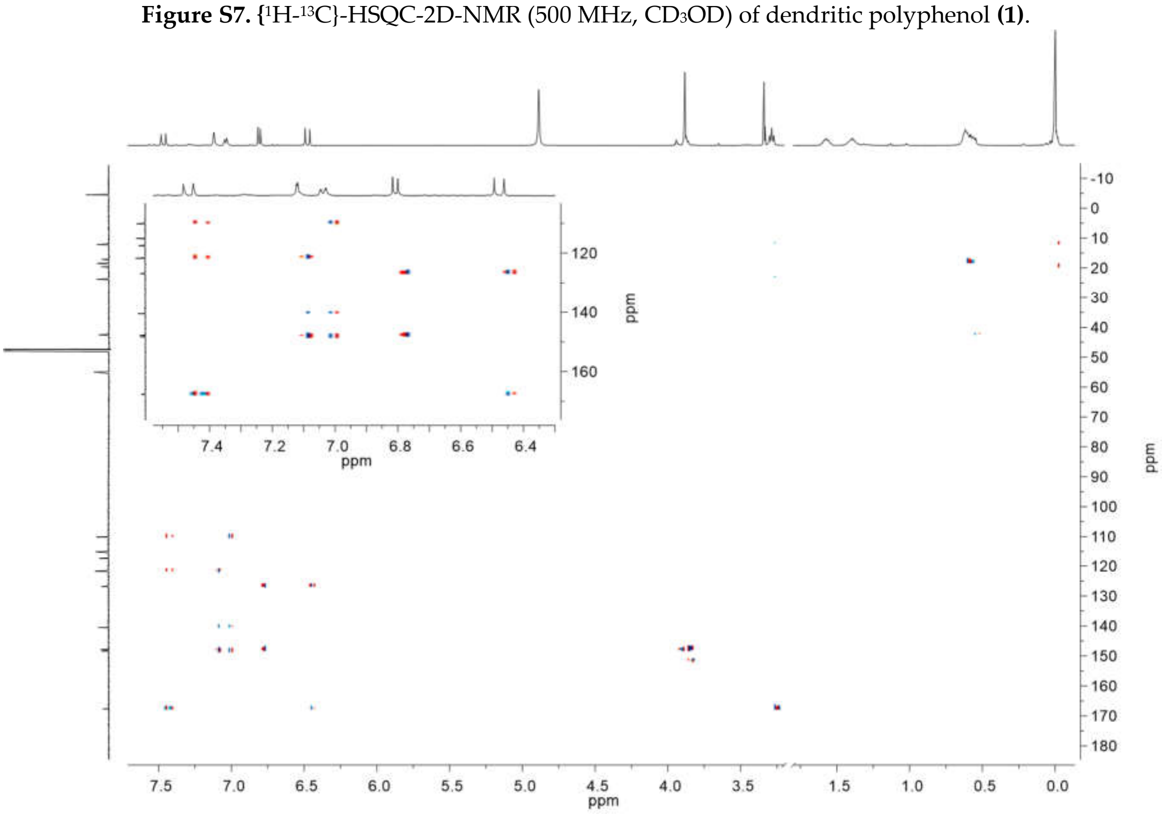

Figure S8. {^{1}H-^{13}C}-HMBC-2D-NMR (500 MHz, CD_3OD) of dendritic polyphenol **(1)**.

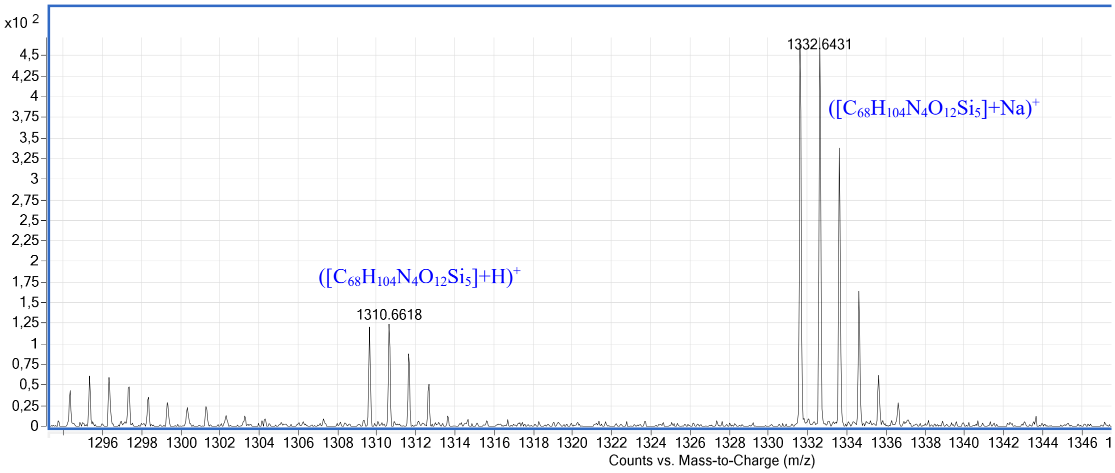

Figure S9. Mass Spectrometry (ESI-TOF) of dendritic polyphenol **(2)**.

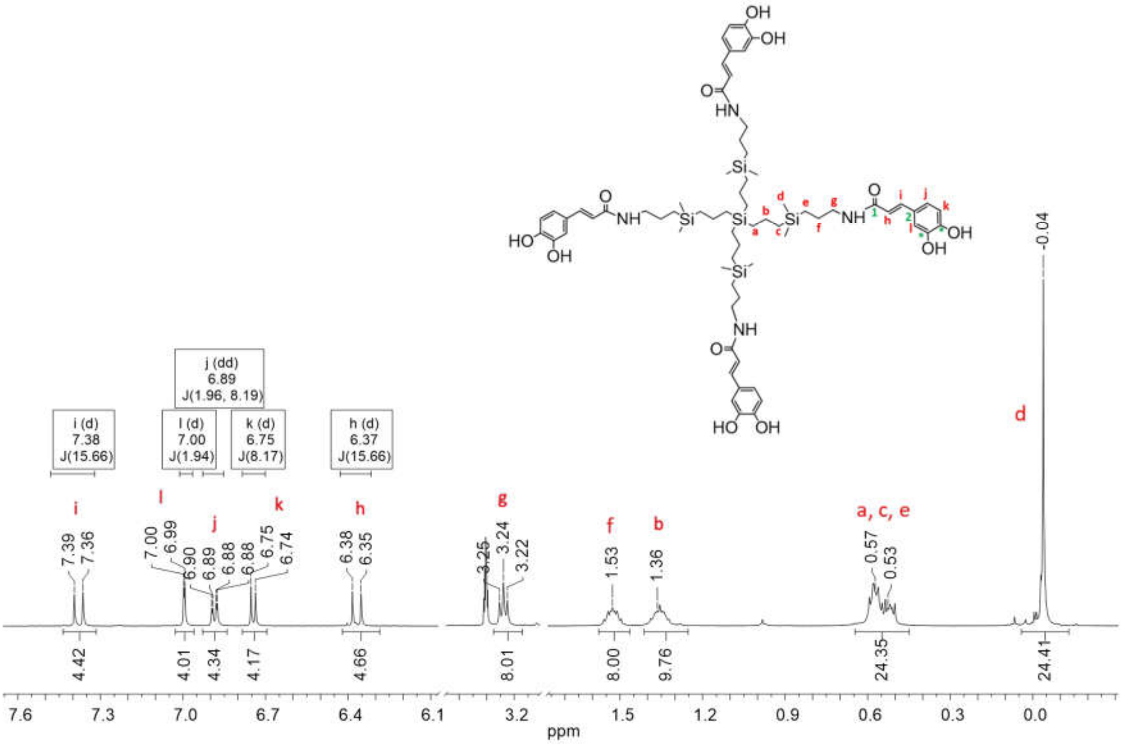

Figure S10. ^{1}H-NMR (500 MHz, CD_3OD) of dendritic polyphenol **(2)**.

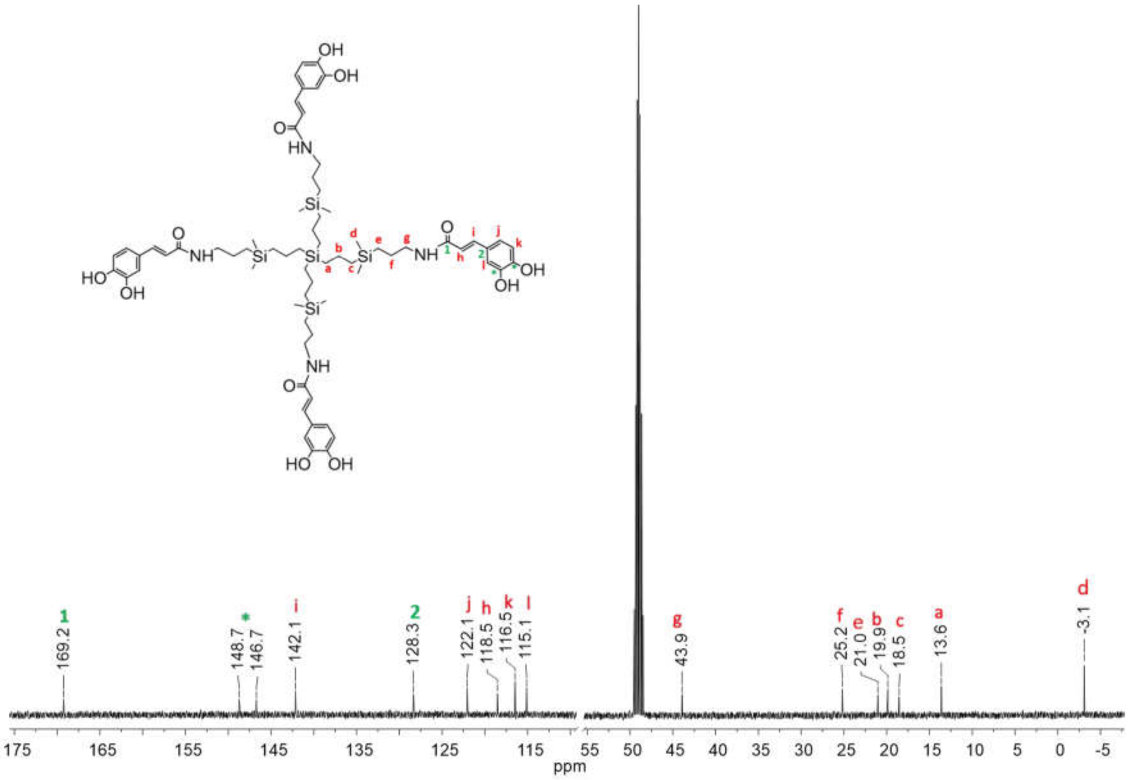

Figure S11. ^{13}C-NMR (500 MHz, CD_3OD) of dendritic polyphenol **(2)**.

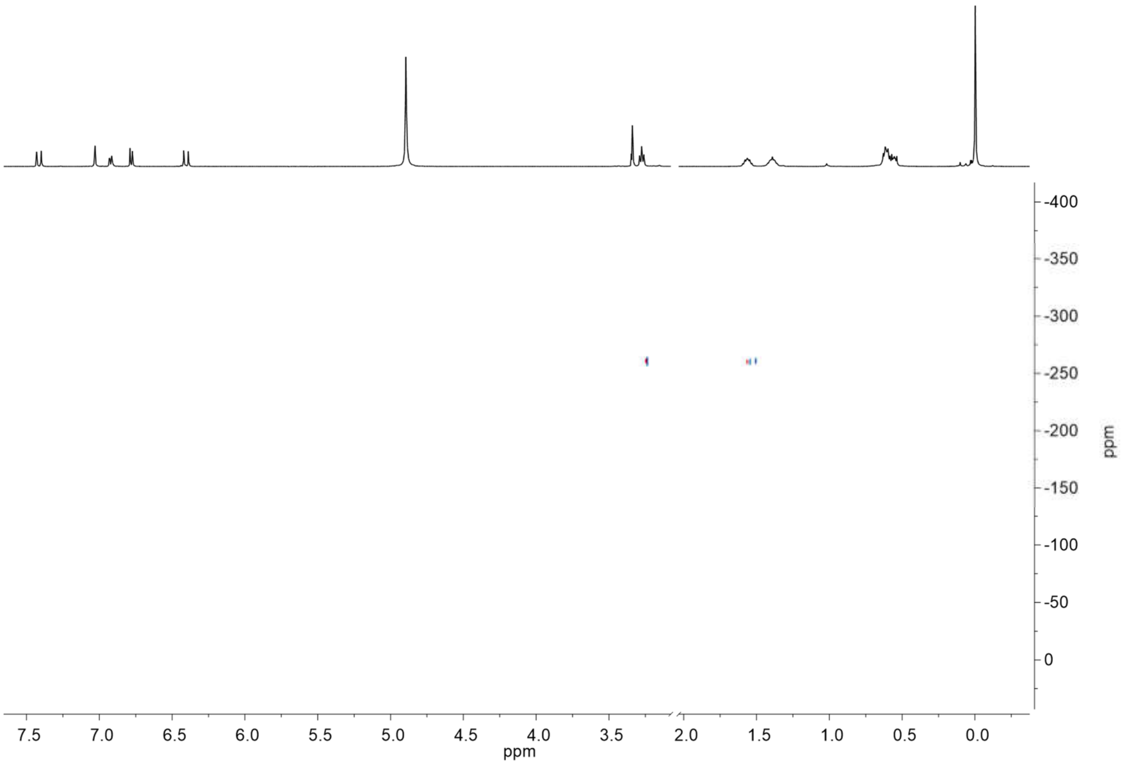

Figure S12. {^{1}H-^{15}N}-HMBC-NMR (500 MHz, CD_3OD) of dendritic polyphenol **(2)**.

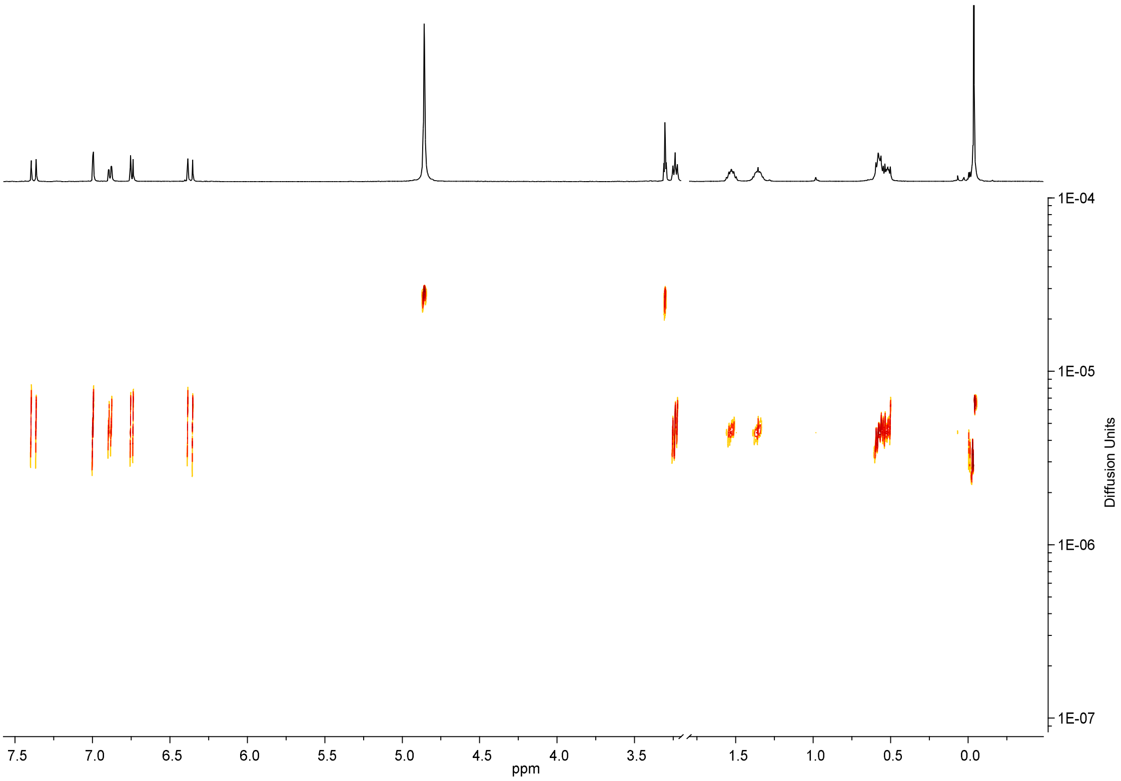

Figure S13. ^{1}H-DOSY-2D-NMR (500 MHz, CD_3OD) of dendritic polyphenol **(2)**.

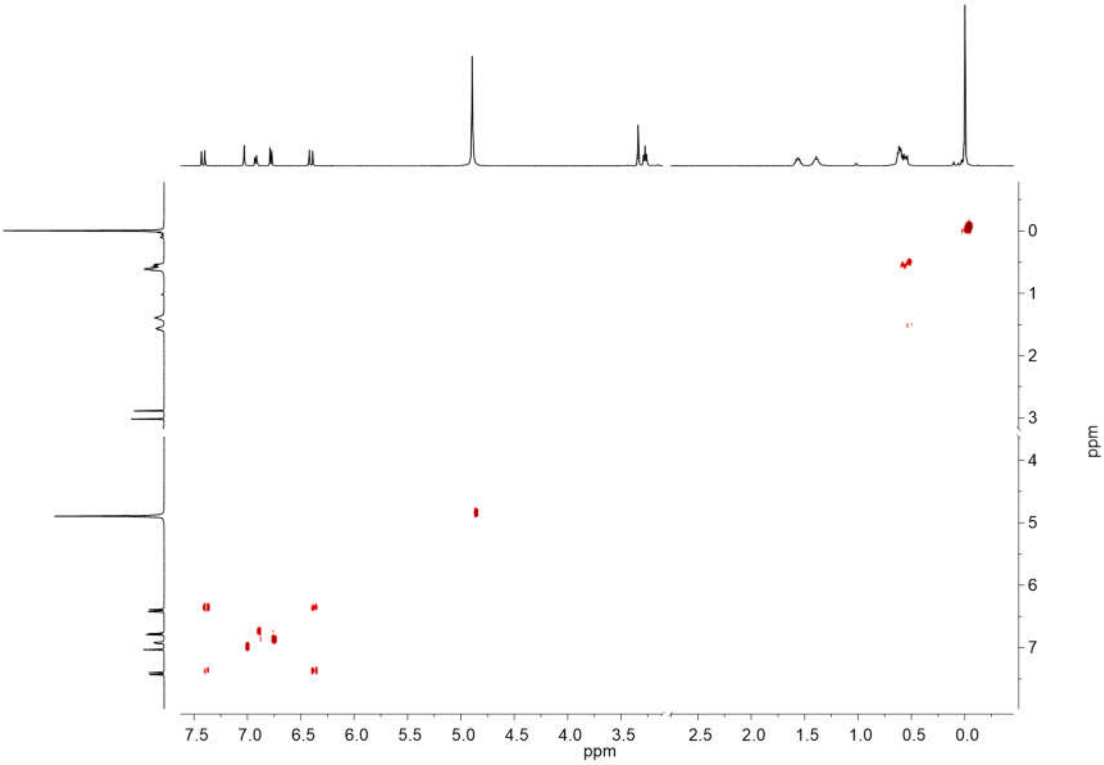

Figure S14. {^{1}H-^{1}H}-COSY-2D-NMR (500 MHz, CD_3OD) of dendritic polyphenol **(2)**.

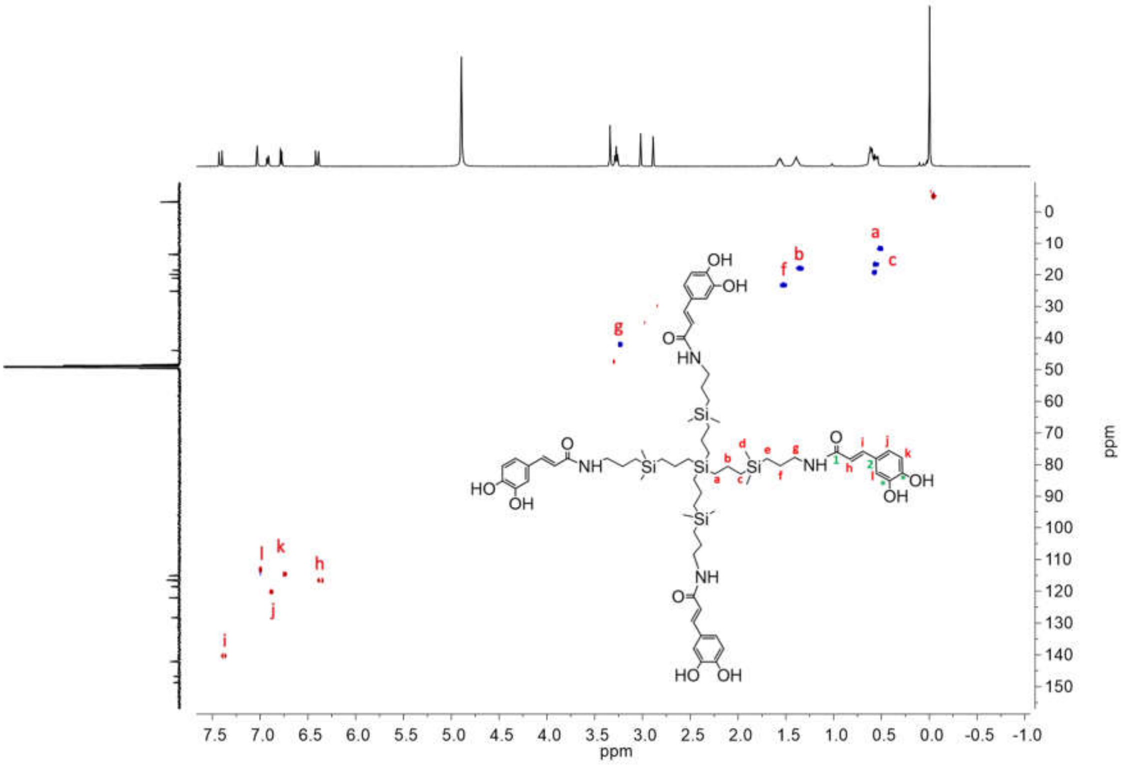

Figure S15. {^{1}H-^{13}C}-HSQC-2D-NMR (500 MHz, CD_3OD) of dendritic polyphenol **(2).**

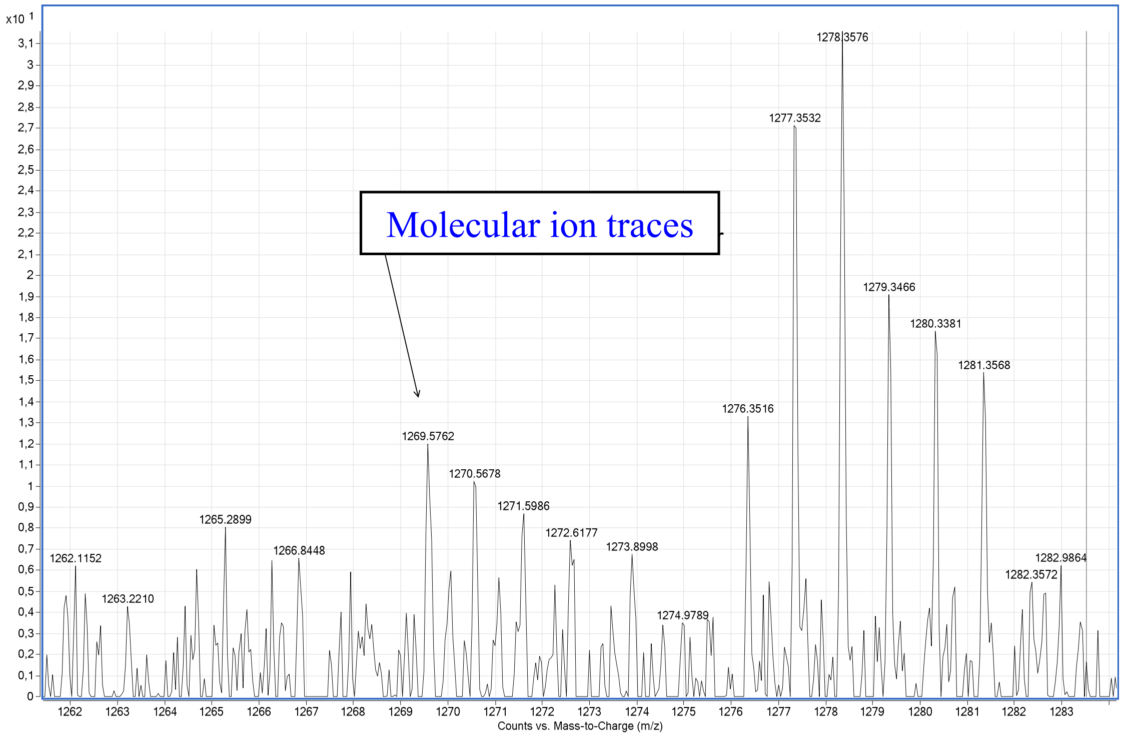

Figure S16. Mass Spectrometry (ESI-TOF) of dendritic polyphenol **(3)**.

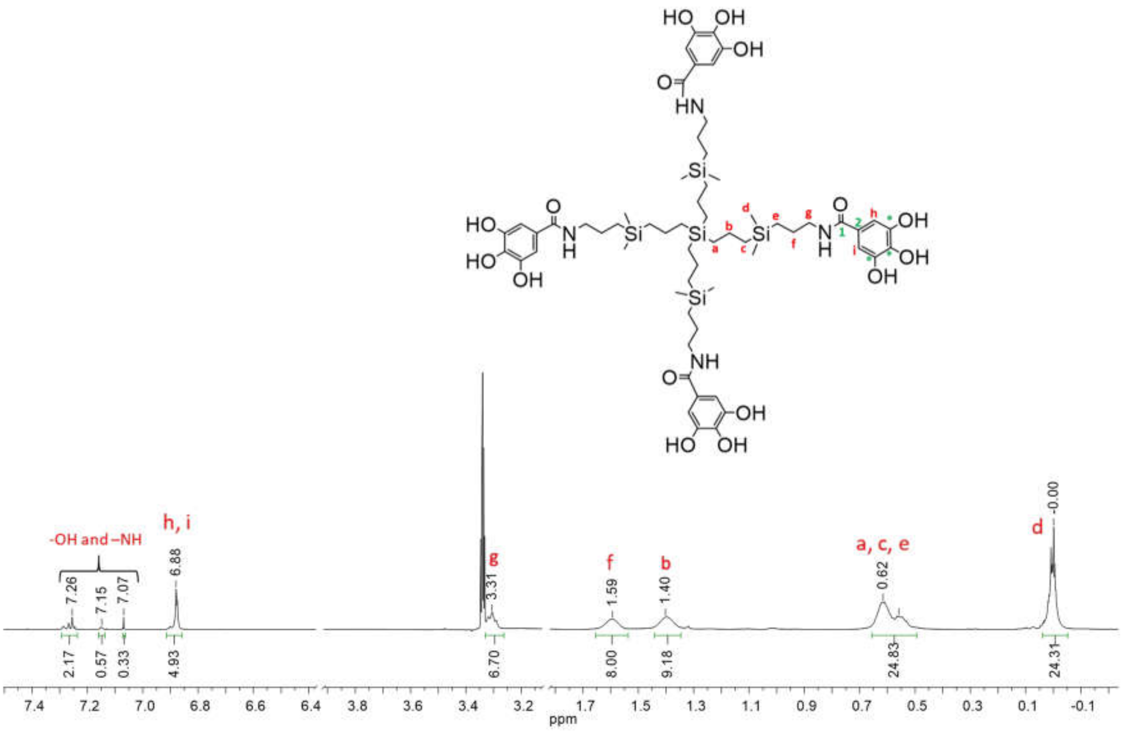

Figure S17. ^{1}H-NMR (500 MHz, CD_3OD) of dendritic polyphenol **(3)**.

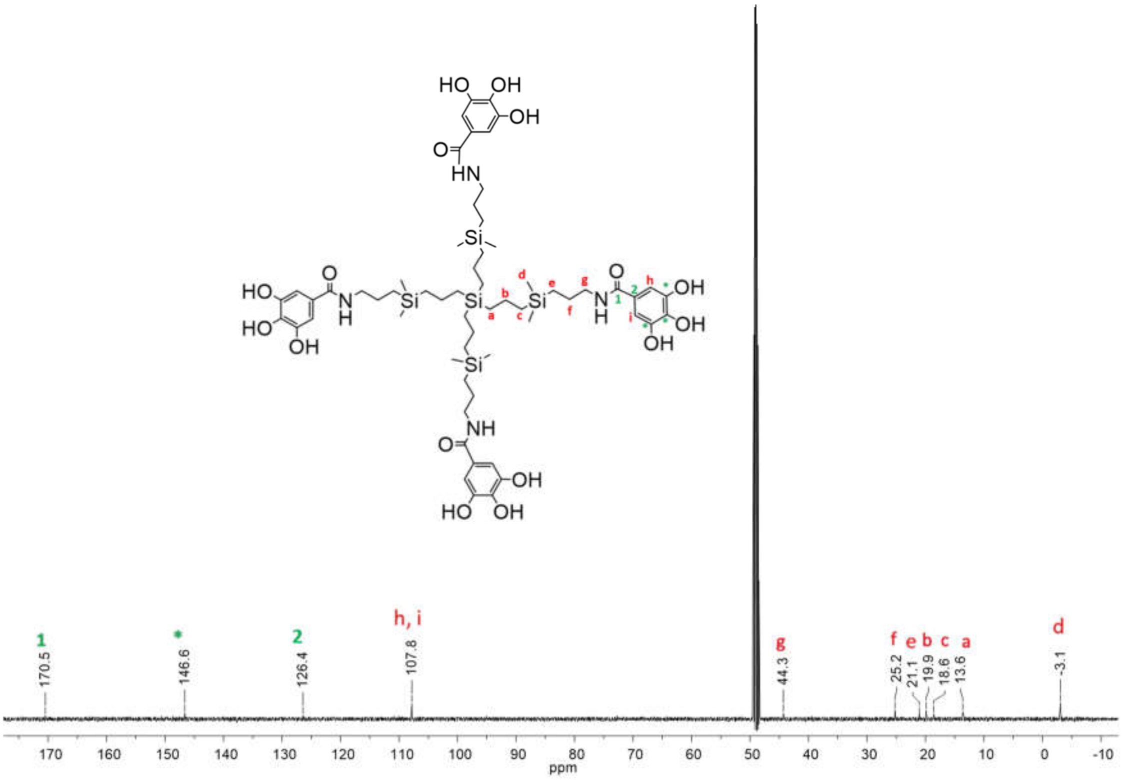

Figure S18. ^{13}C-NMR (500 MHz, CD_3OD) of dendritic polyphenol **(3)**.

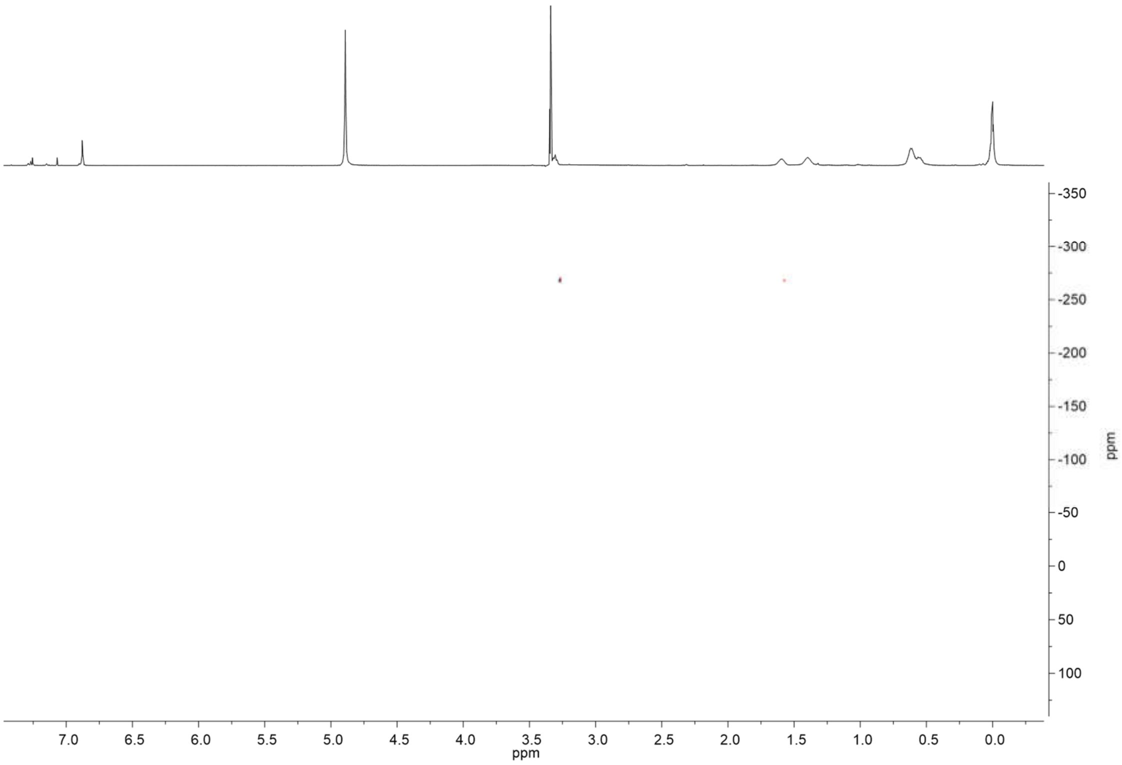

Figure S19. {^{1}H-^{15}N}-HMBC-NMR (500 MHz, CD_3OD) of dendritic polyphenol **(3)**.

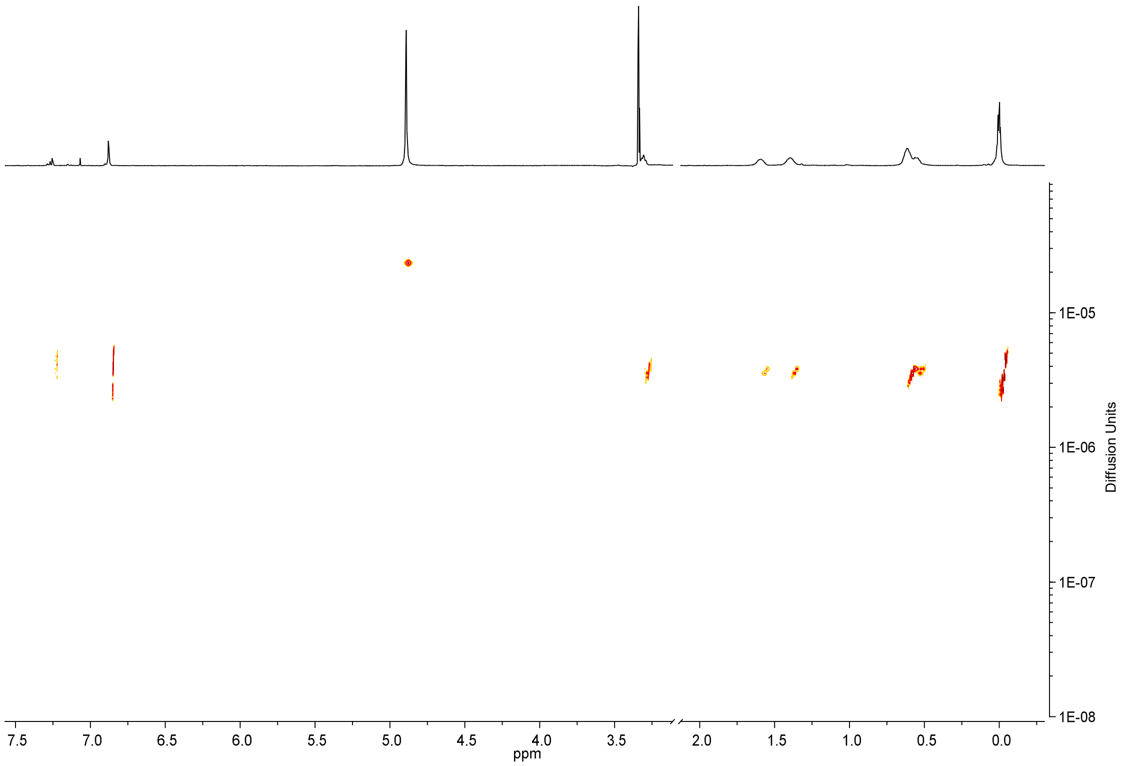

Figure S20. ^{1}H-DOSY-2D-NMR (500 MHz, CD_3OD) of dendritic polyphenol **(3)**.

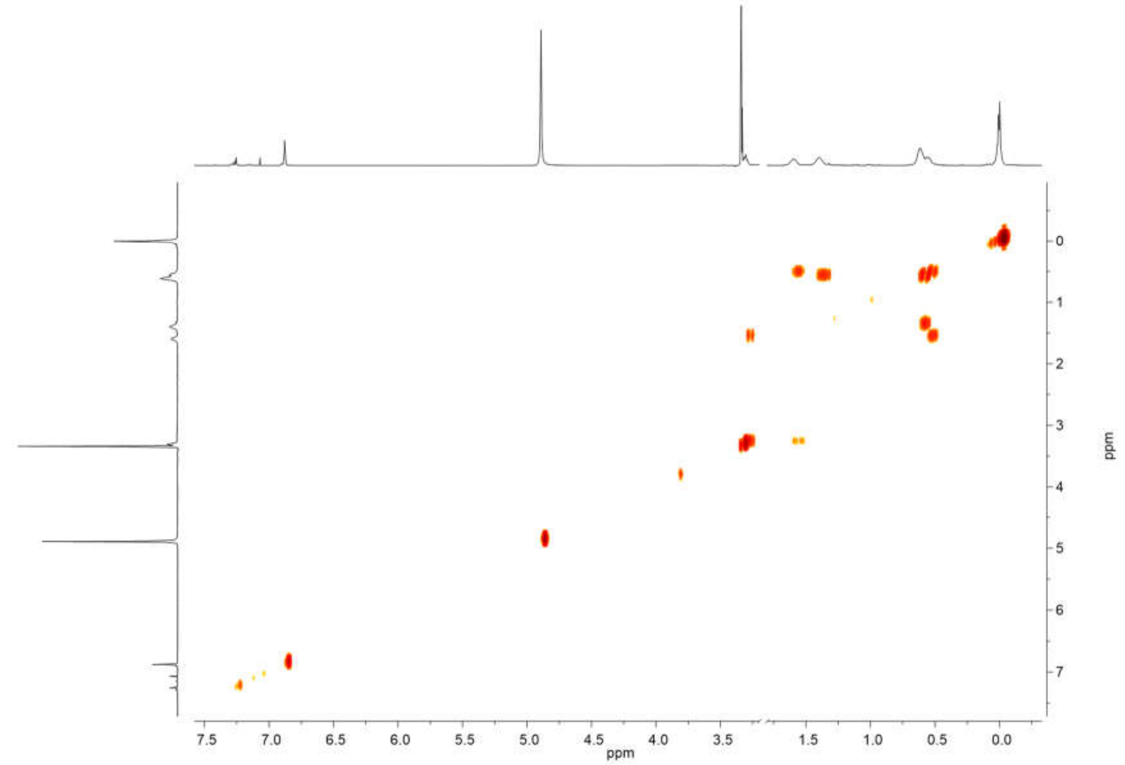

Figure S21. {^{1}H-^{1}H}-COSY-2D-NMR (500 MHz, CD_3OD) of dendritic polyphenol **(3)**.

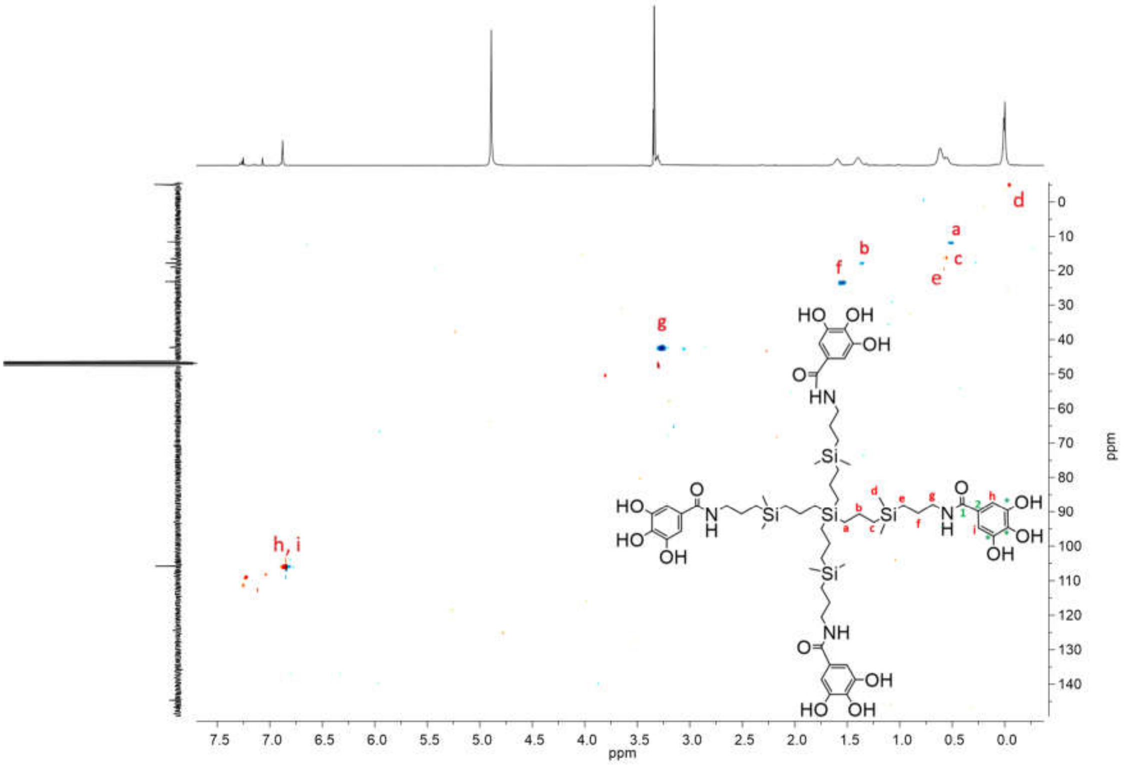

Figure S22. {^{1}H-^{13}C}-HSQC-2D-NMR (500 MHz, CD_3OD) of dendritic polyphenol **(3)**.

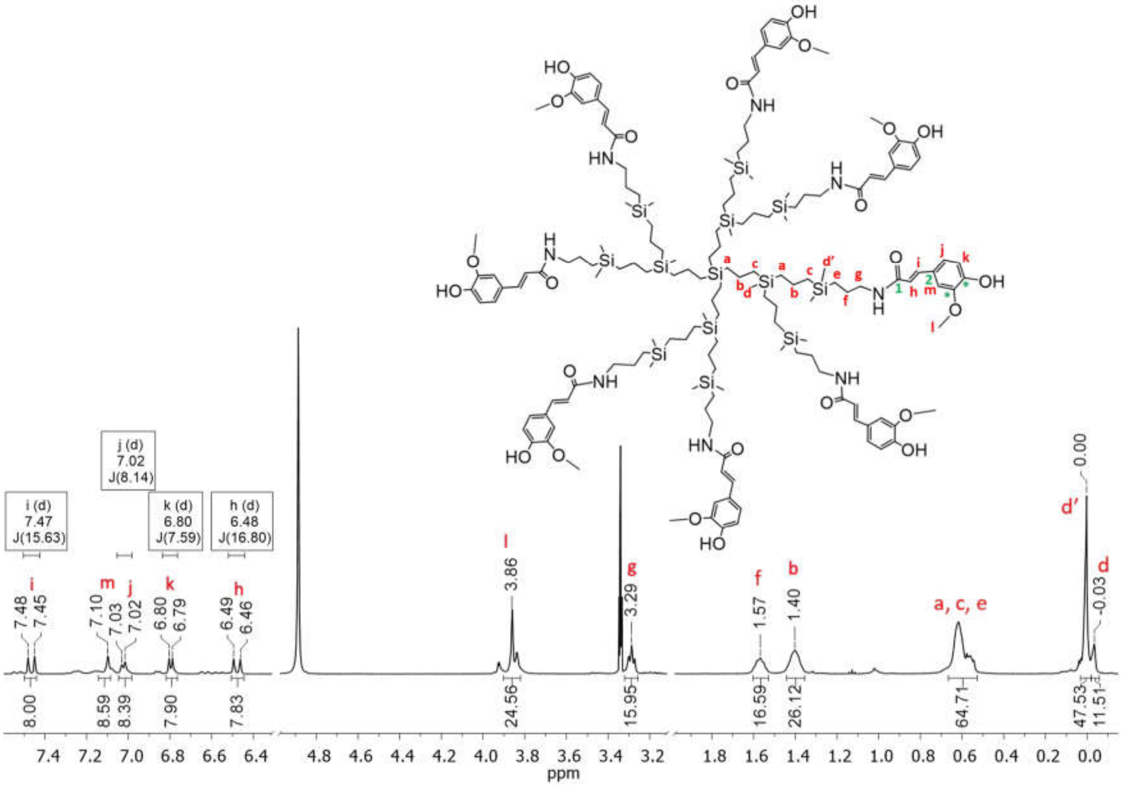

Figure S23. ^{1}H-NMR (500 MHz, CD_3OD) of dendritic polyphenol **(4)**.

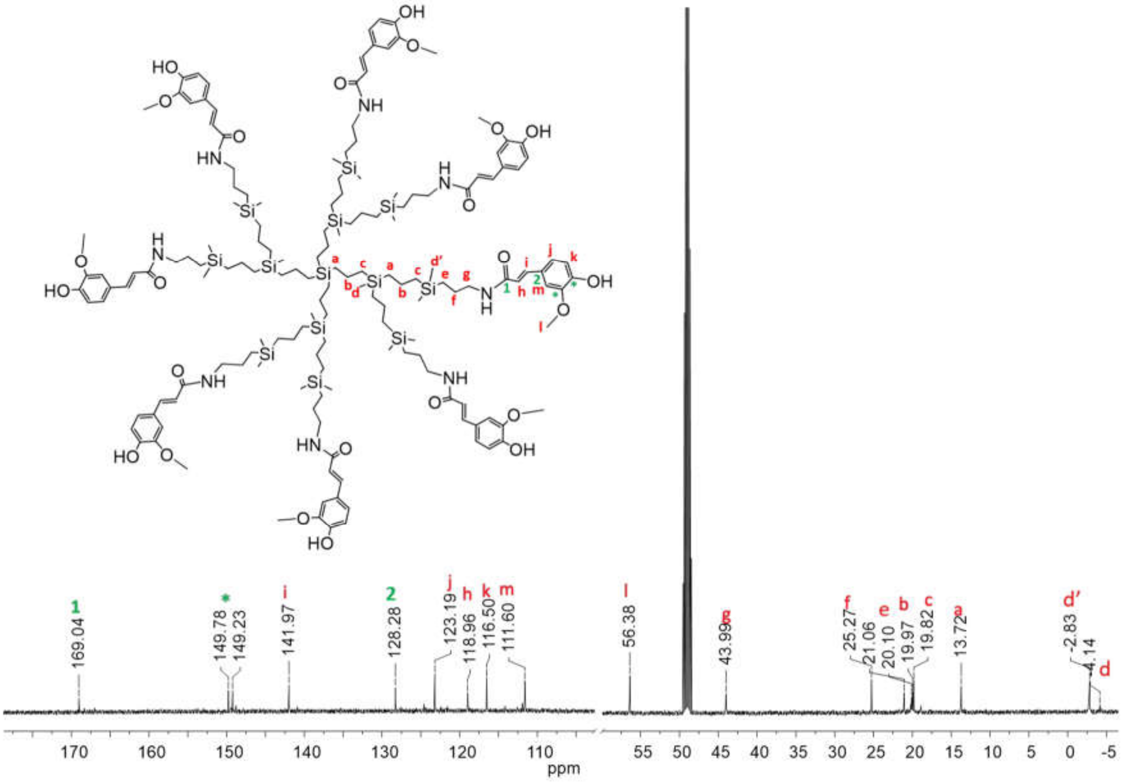

Figure S24. ^{13}C-NMR (500 MHz, CD_3OD) of dendritic polyphenol **(4)**.

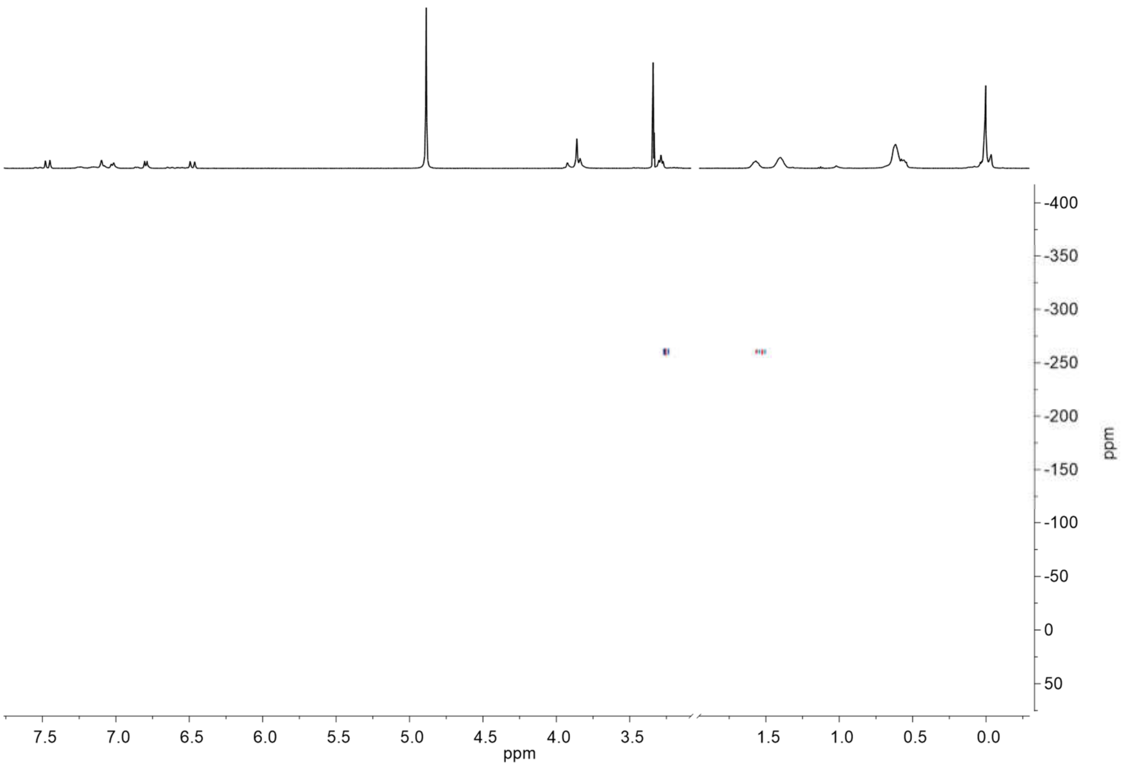

Figure S25. {^{1}H-^{15}N}-HMBC-NMR (500 MHz, CD_3OD) of dendritic polyphenol **(4)**.

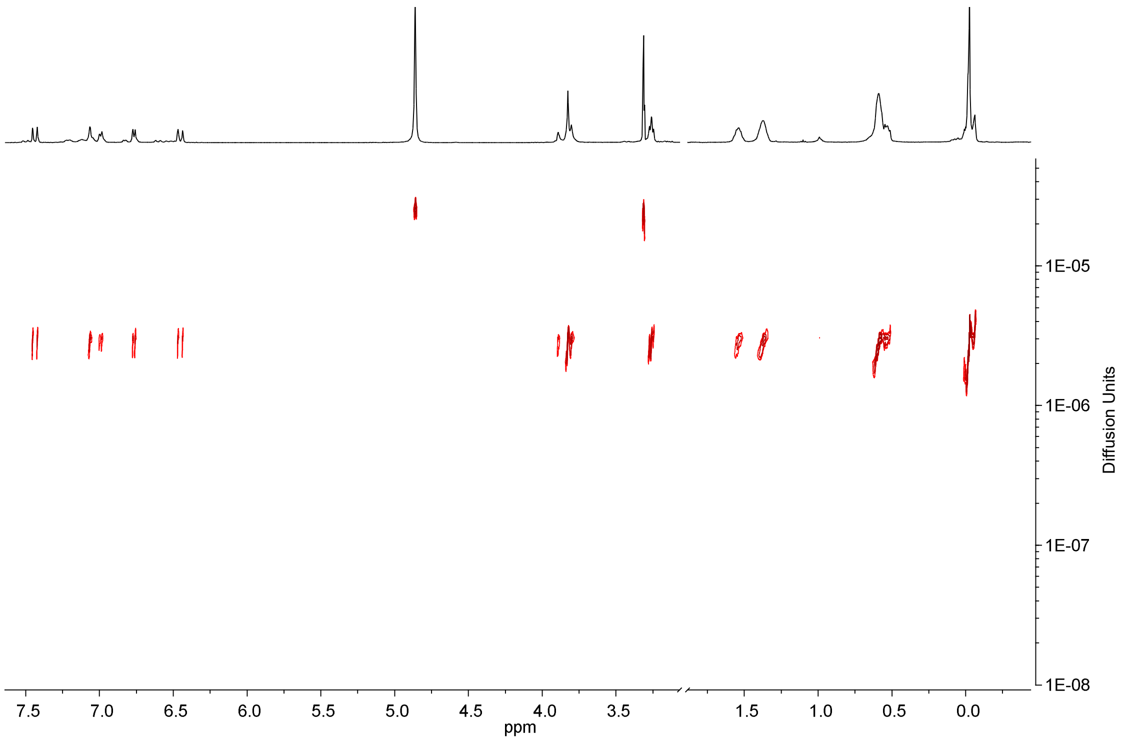

Figure S26. ^{1}H-DOSY-2D-NMR (500 MHz, CD_3OD) of dendritic polyphenol **(4)**.

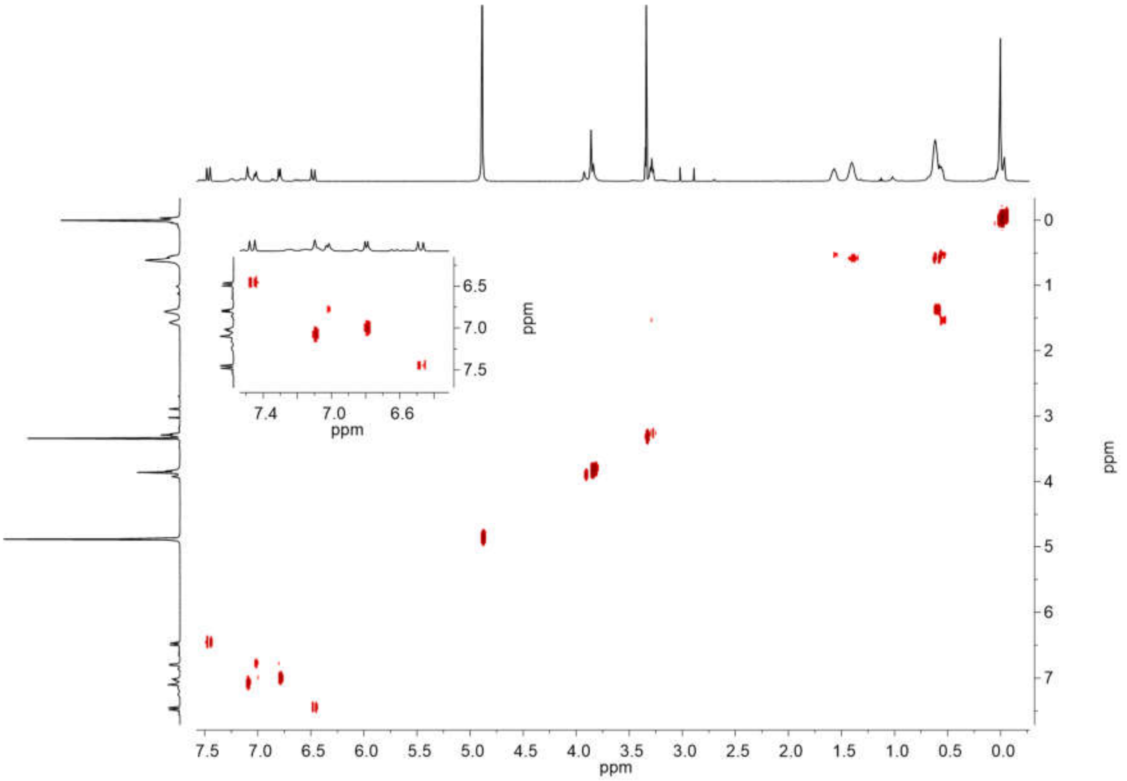

Figure S27. {^{1}H-^{1}H}-COSY-2D-NMR (500 MHz, CD_3OD) of dendritic polyphenol **(4)**.

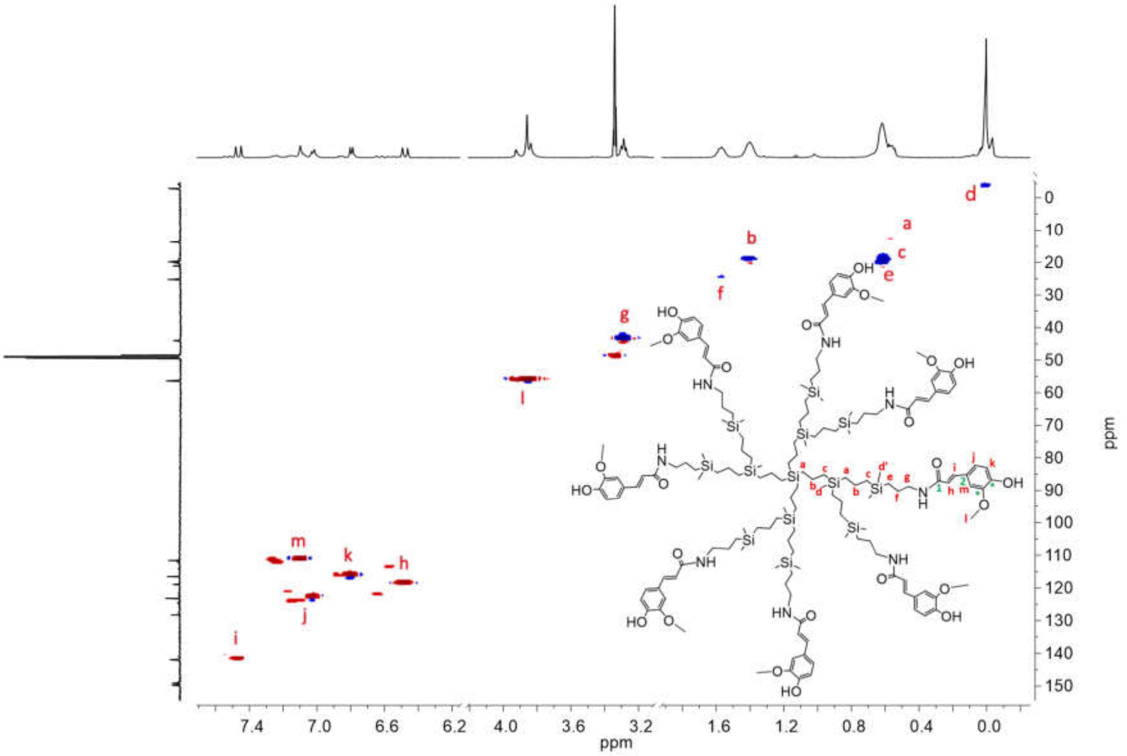

Figure S28. {^{1}H-^{13}C}-HSQC-2D-NMR (500 MHz, CD_3OD) of dendritic polyphenol **(4)**.

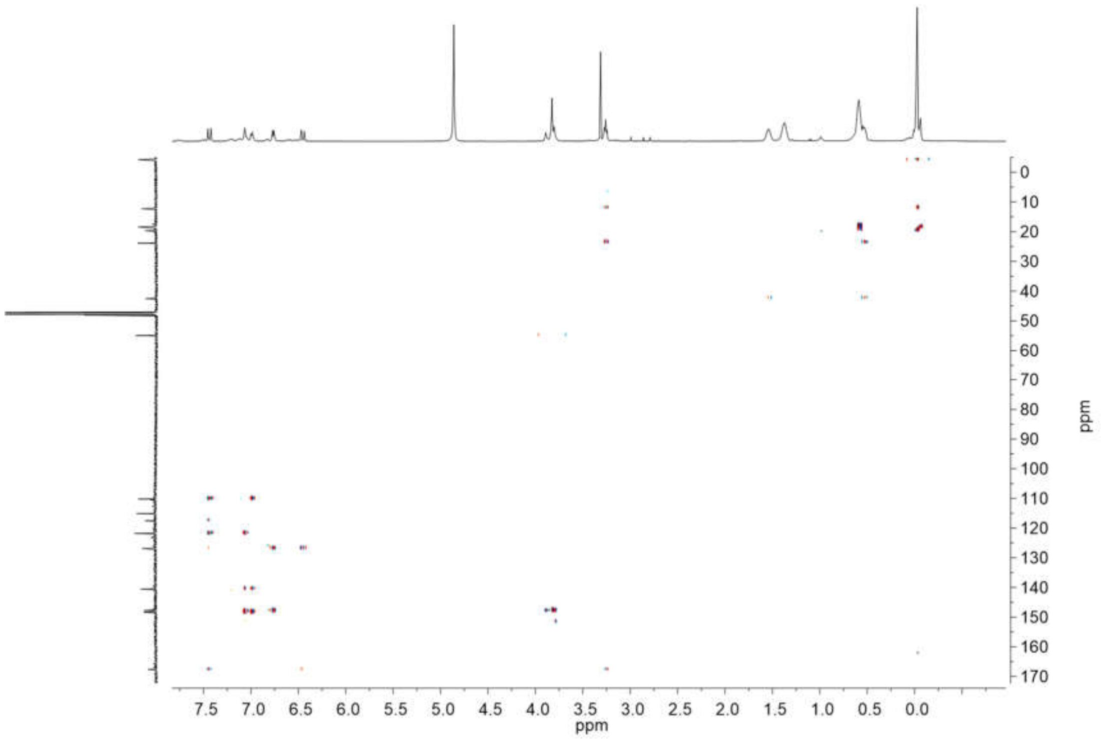

Figure S29. {^{1}H-^{13}C}-HMBC-2D-NMR (500 MHz, CD_3OD) of dendritic polyphenol **(4)**.

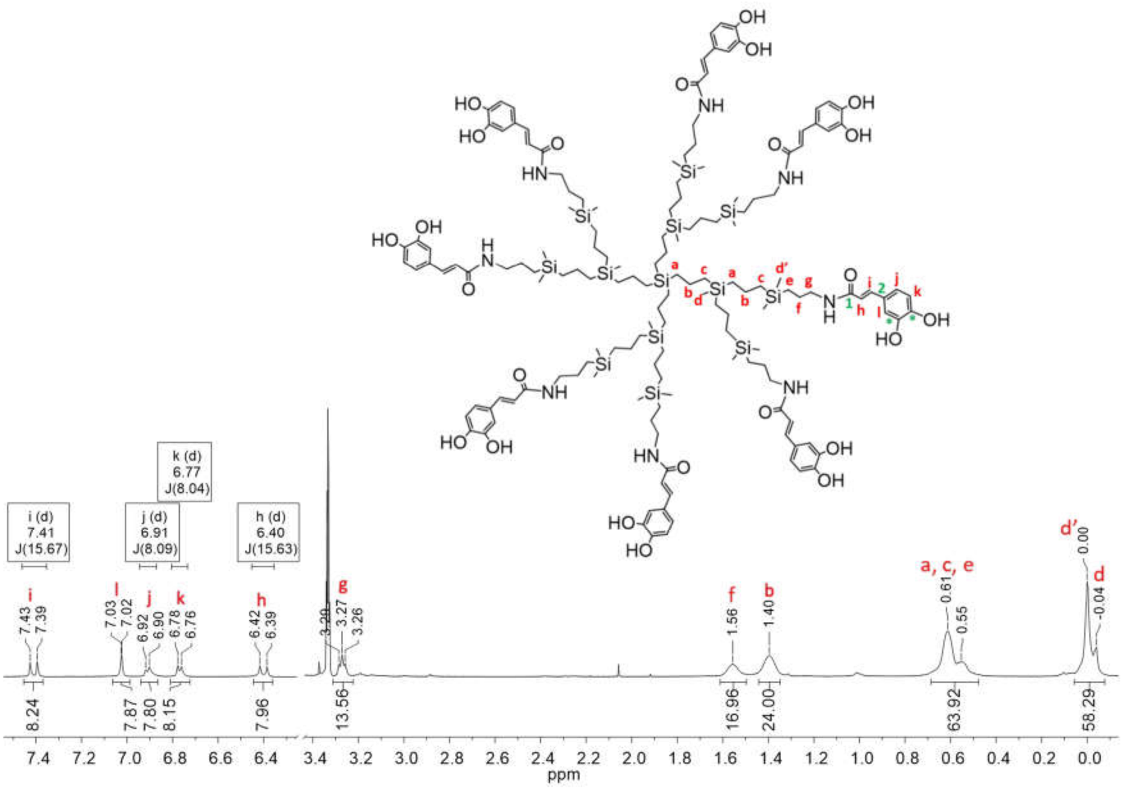

Figure S30. ^{1}H-NMR (500 MHz, CD_3OD) of dendritic polyphenol **(5)**.

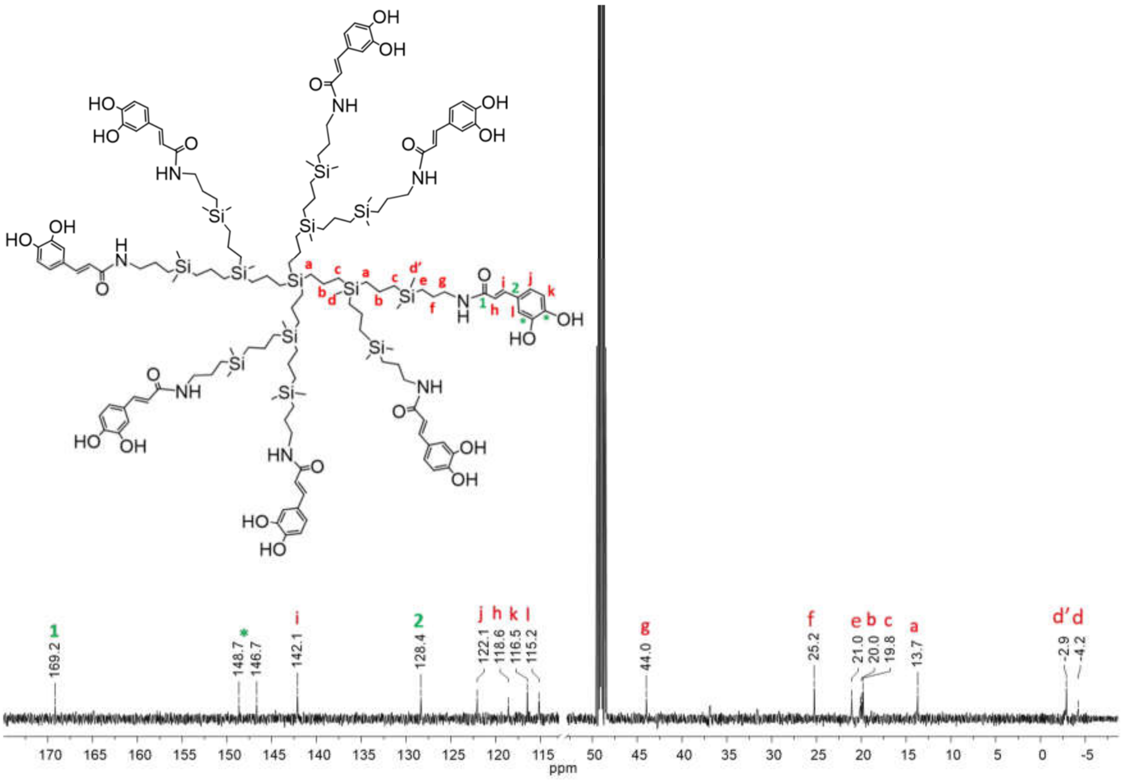

Figure S31. ^{13}C-NMR (500 MHz, CD_3OD) of dendritic polyphenol **(5)**.

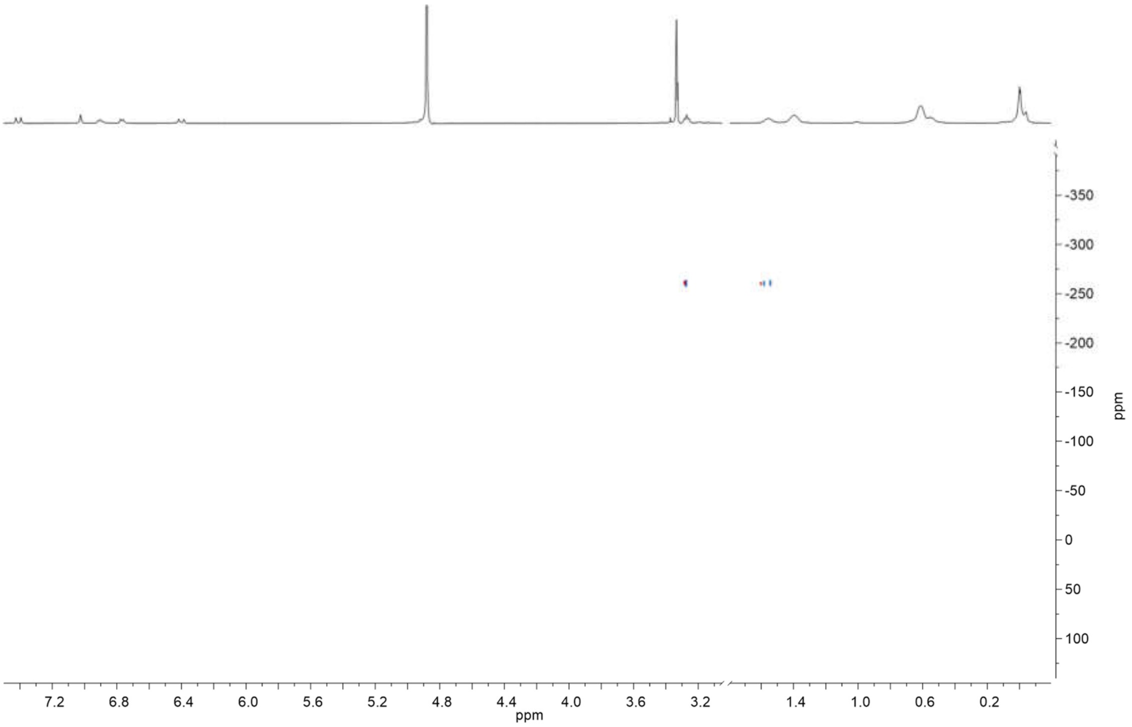

Figure S32. {^{1}H-^{15}N}-HMBC-NMR (500 MHz, CD_3OD) of dendritic polyphenol **(5)**.

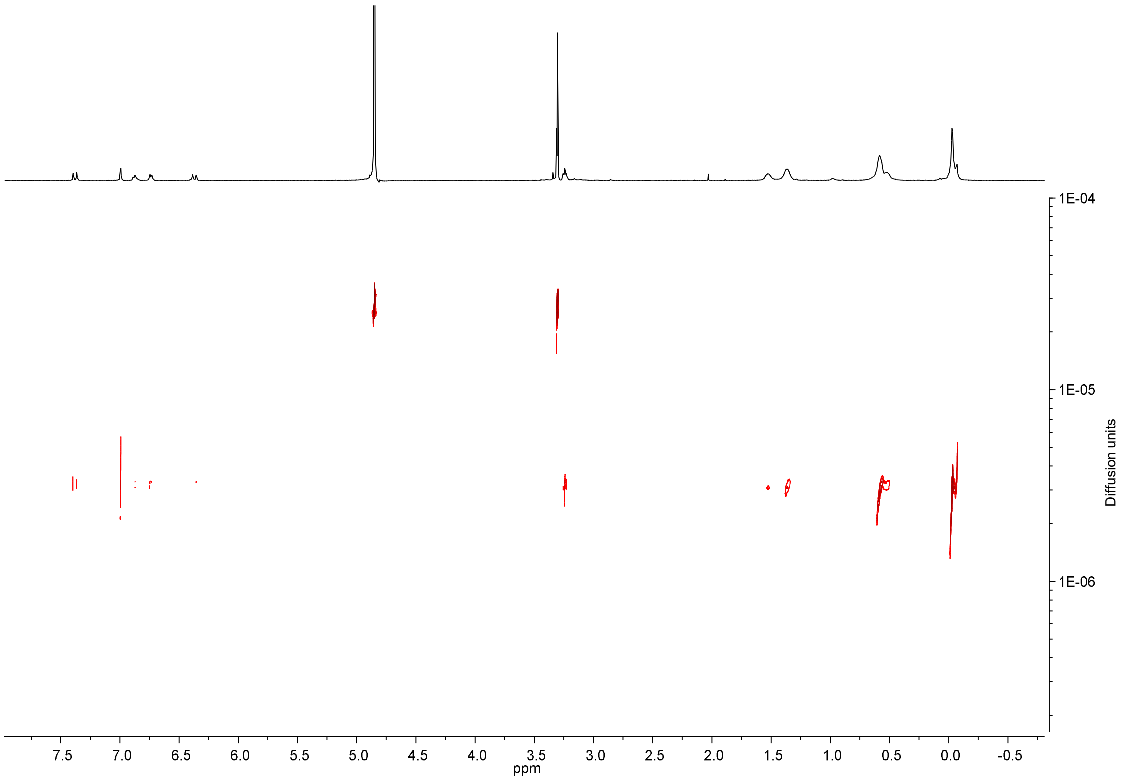

Figure S33. ^{1}H-DOSY-2D-NMR (500 MHz, CD_3OD) of dendritic polyphenol **(5)**.

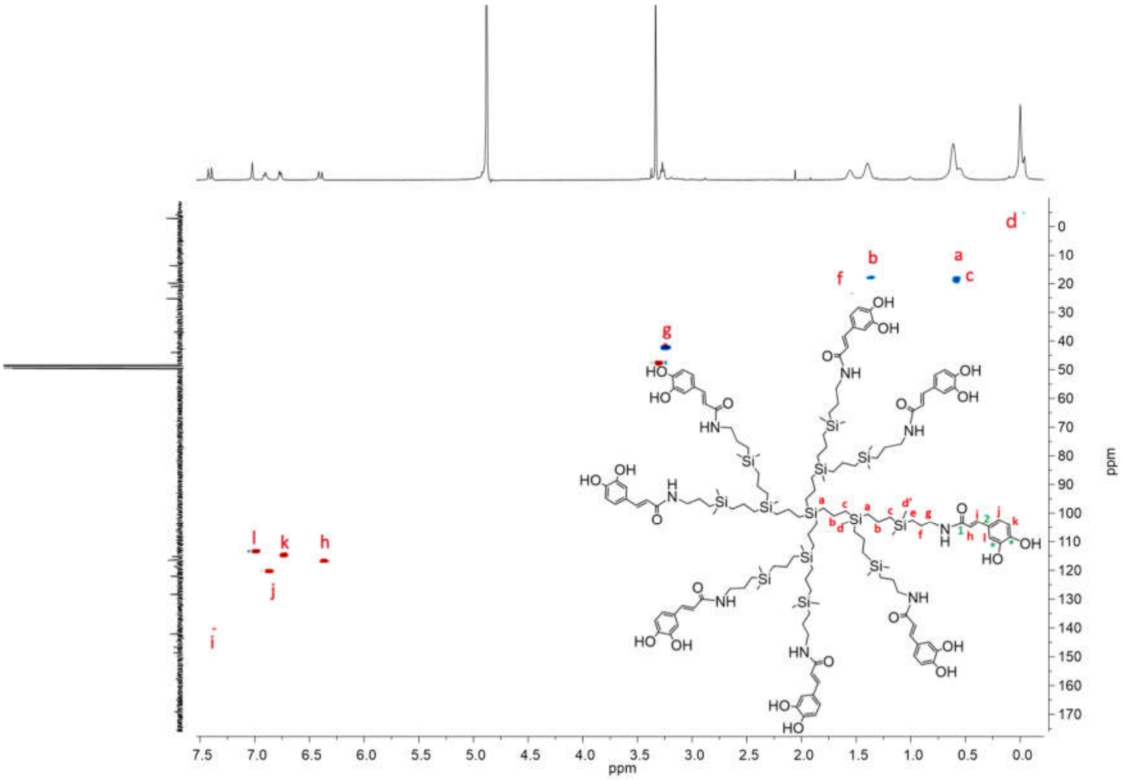

Figure S34. {^{1}H-^{13}C}-HSQC-2D-NMR (500 MHz, CD_3OD) of dendritic polyphenol **(5)**.

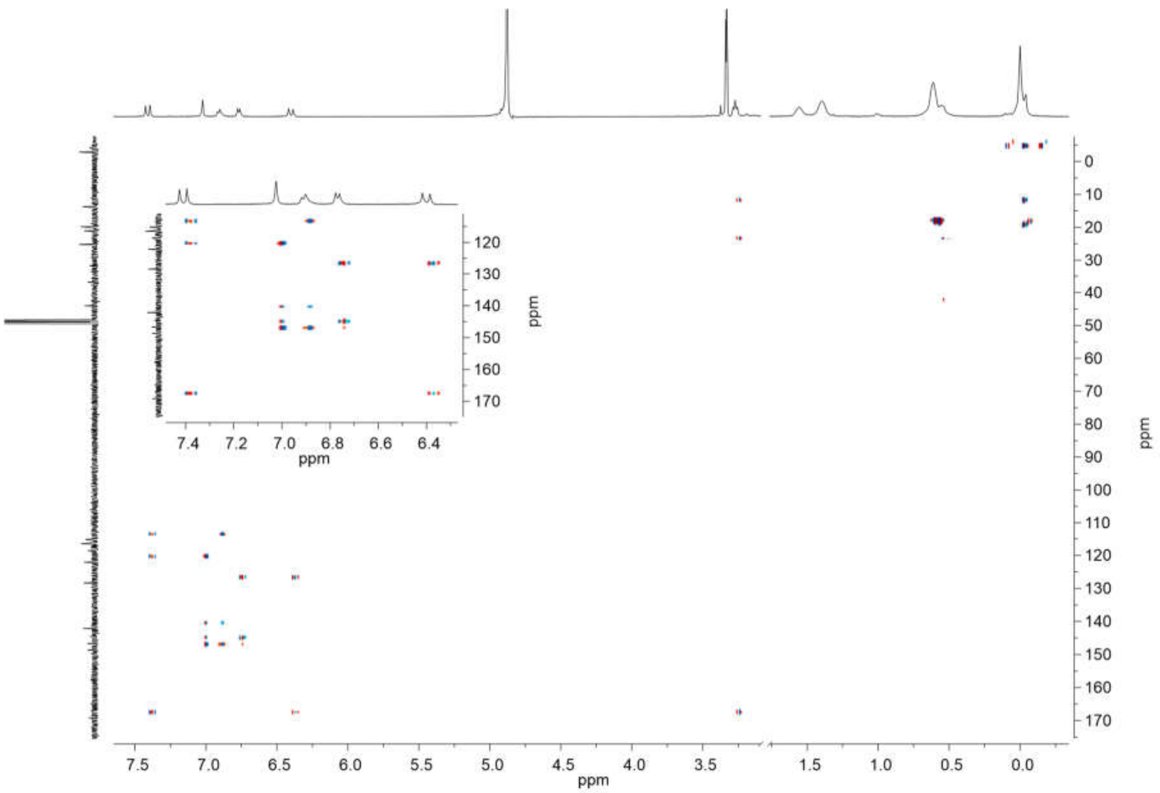

Figure S35. {^{1}H-^{13}C}-HMBC-2D-NMR (500 MHz, CD_3OD) of dendritic polyphenol **(5)**.

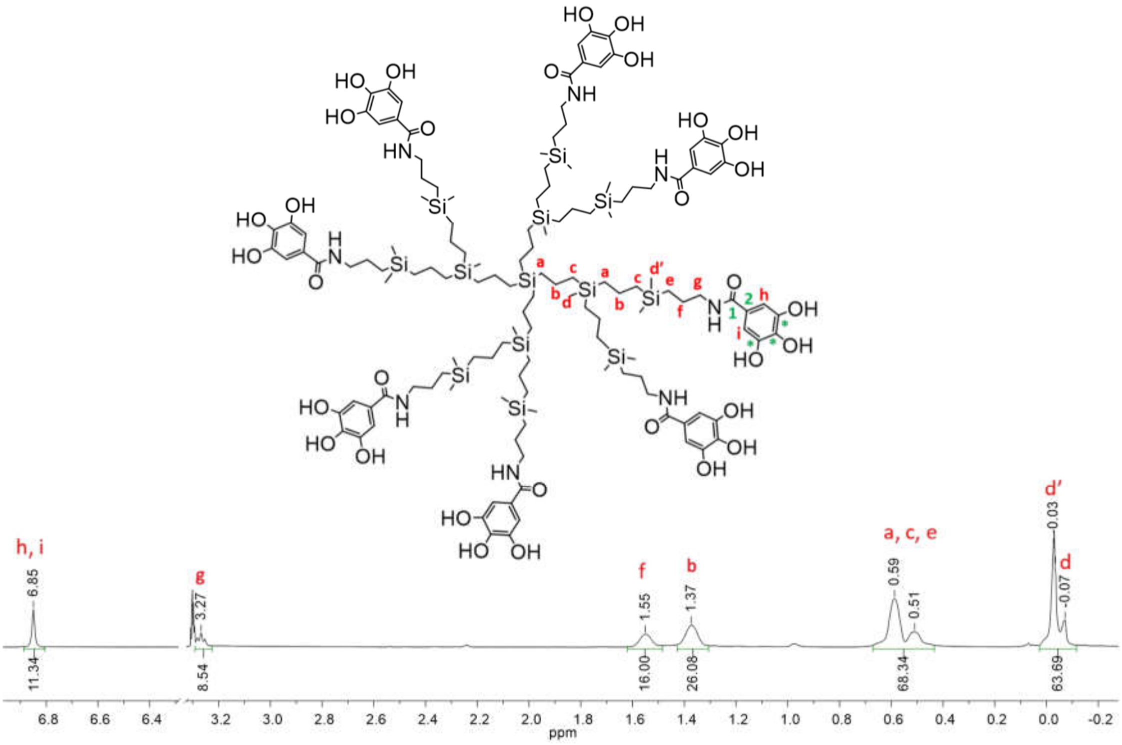

Figure S36. ^{1}H-NMR (500 MHz, CD_3OD) of dendritic polyphenol **(6)**.

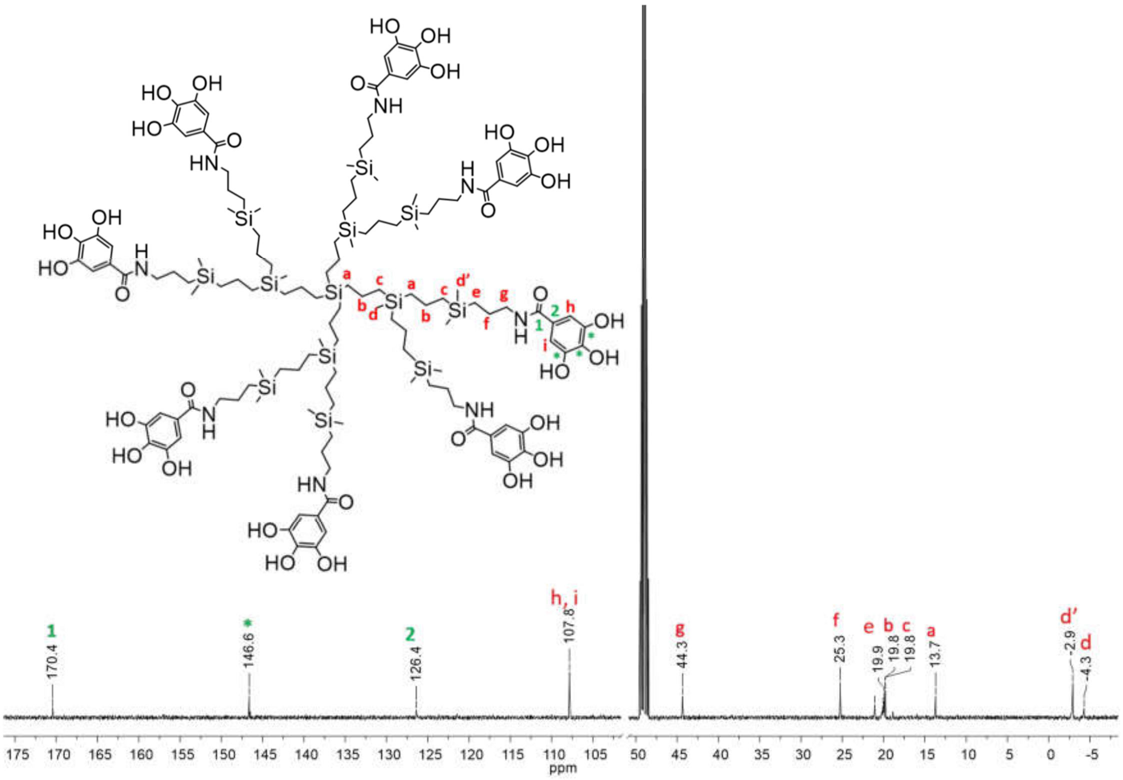

Figure S37. ^{13}C-NMR (500 MHz, CD_3OD) of dendritic polyphenol **(6)**.

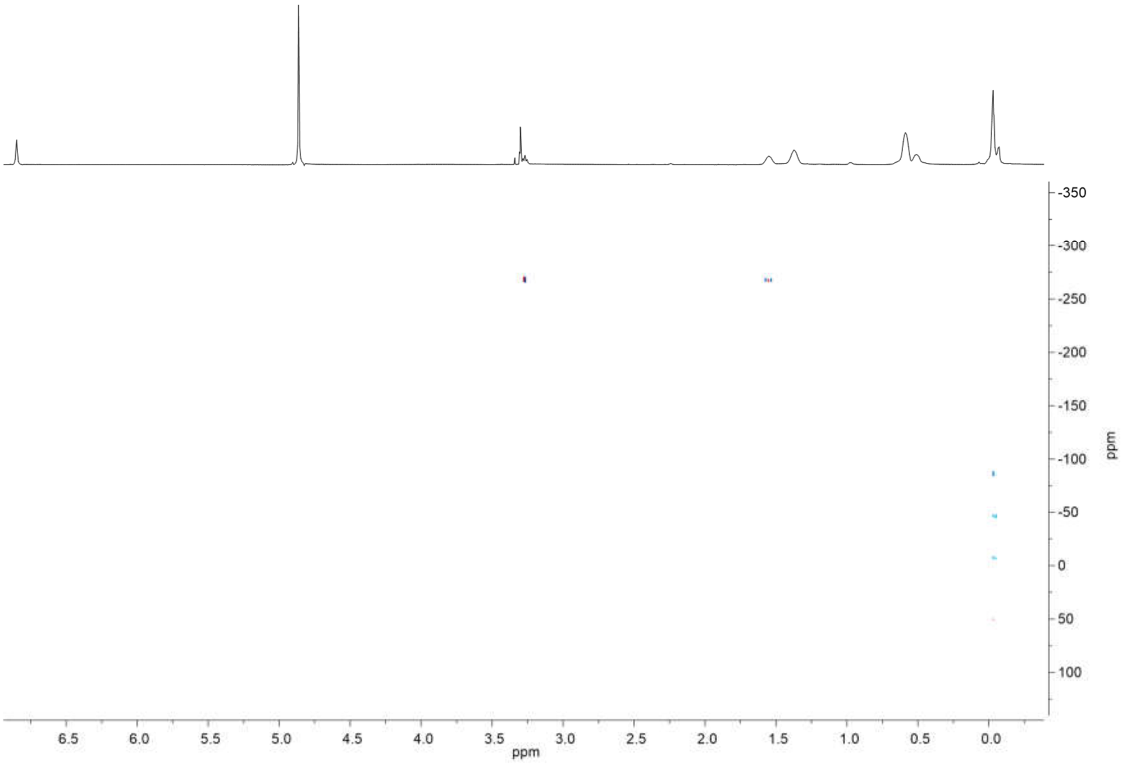

Figure S38. {^{1}H-^{15}N}-HMBC-NMR (500 MHz, CD_3OD) of dendritic polyphenol **(6)**.

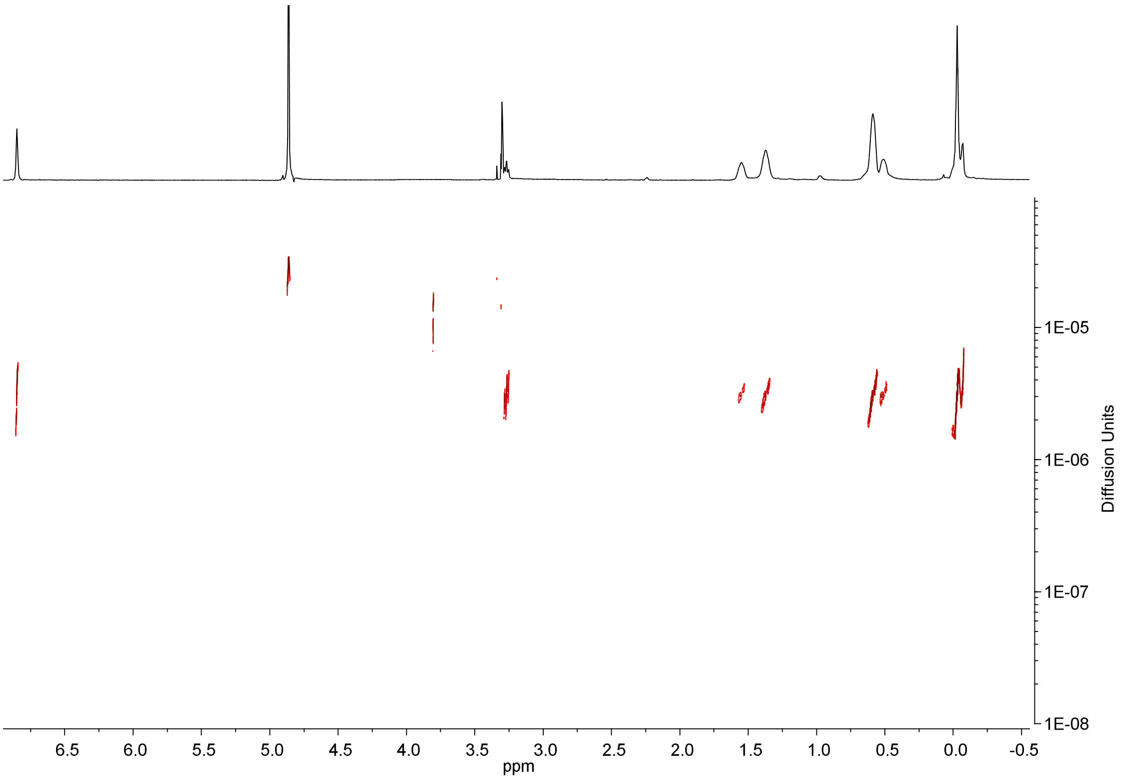

Figure S39. ^{1}H-DOSY-2D-NMR (500 MHz, CD_3OD) of dendritic polyphenol **(6)**.

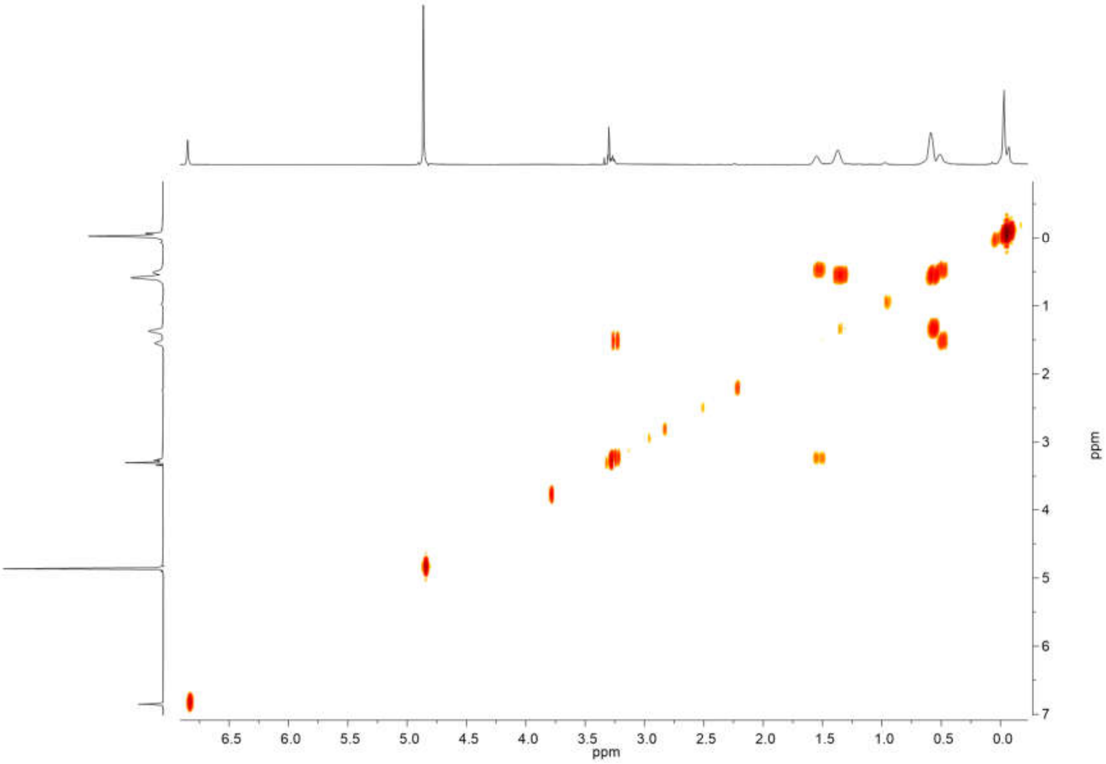

Figure S40. {^{1}H-^{1}H}-COSY-2D-NMR (500 MHz, CD_3OD) of dendritic polyphenol **(6)**.

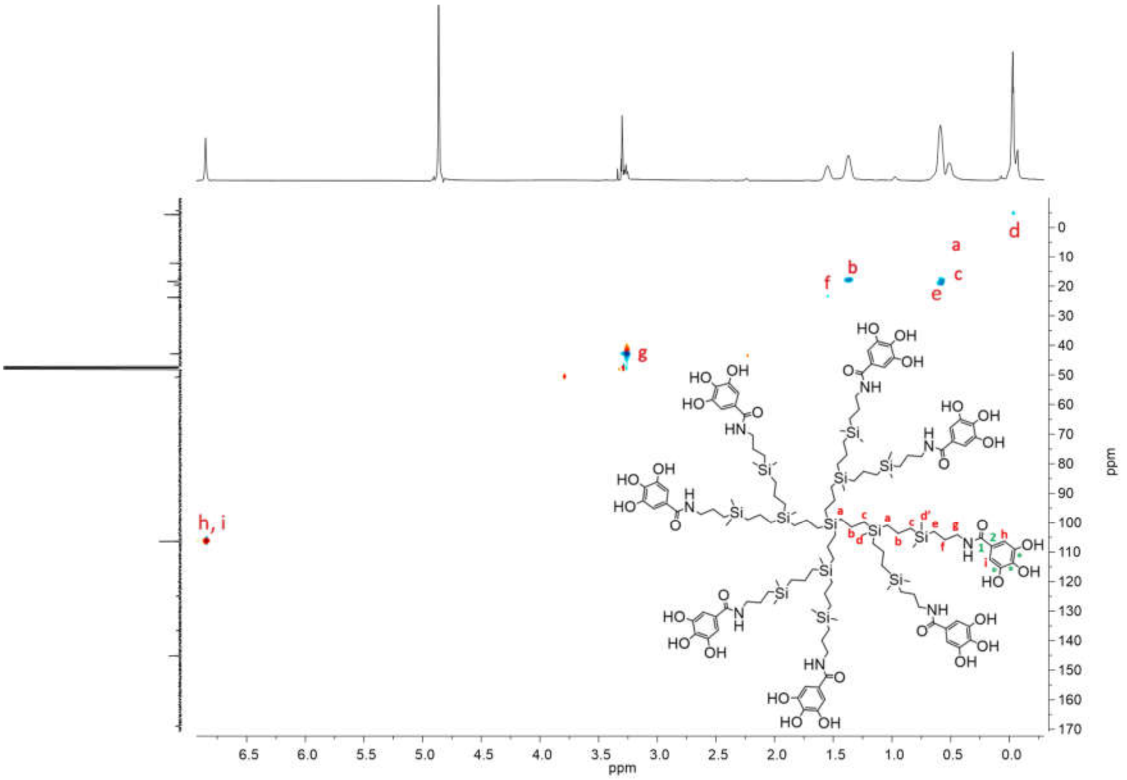

Figure S41. {^{1}H-^{13}C}-HSQC-2D-NMR (500 MHz, CD_3OD) of dendritic polyphenol **(6)**.

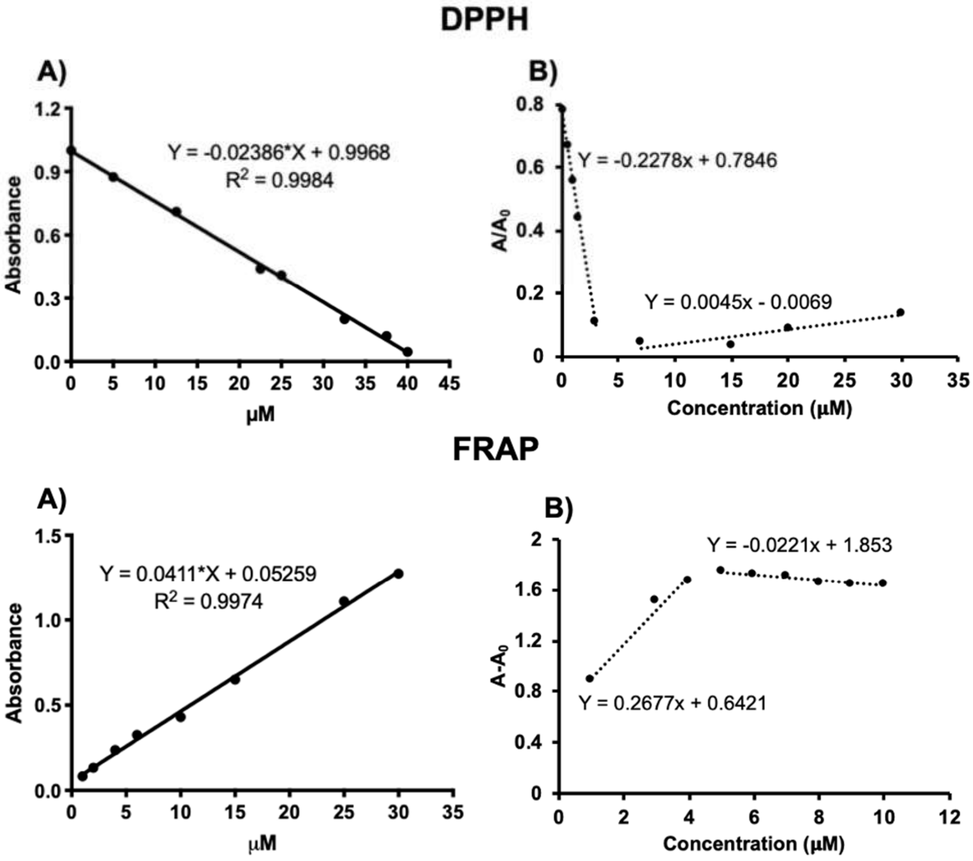

Figure S42. A) A representative calibration curve of inhibition of DPPH by Trolox standards. Representative results of at least three independent experiments are shown. B) Graphics with equations line for compound G_1-$[Si(CH_2)_3NH(CO)Ph(OH)_3]_4$ **(3)**.
